# High-frequency oscillations and sequence generation in two-population models of hippocampal region CA1

**DOI:** 10.1371/journal.pcbi.1009891

**Published:** 2022-02-17

**Authors:** Wilhelm Braun, Raoul-Martin Memmesheimer

**Affiliations:** 1 Neural Network Dynamics and Computation, Institute of Genetics, University of Bonn, Bonn, Germany; 2 Institute of Computational Neuroscience, University Medical Center Hamburg-Eppendorf, Hamburg, Germany; University College London, UNITED KINGDOM

## Abstract

Hippocampal sharp wave/ripple oscillations are a prominent pattern of collective activity, which consists of a strong overall increase of activity with superimposed (140 − 200 Hz) ripple oscillations. Despite its prominence and its experimentally demonstrated importance for memory consolidation, the mechanisms underlying its generation are to date not understood. Several models assume that recurrent networks of inhibitory cells alone can explain the generation and main characteristics of the ripple oscillations. Recent experiments, however, indicate that in addition to inhibitory basket cells, the pattern requires *in vivo* the activity of the local population of excitatory pyramidal cells. Here, we study a model for networks in the hippocampal region CA1 incorporating such a local excitatory population of pyramidal neurons. We start by investigating its ability to generate ripple oscillations using extensive simulations. Using biologically plausible parameters, we find that short pulses of external excitation triggering excitatory cell spiking are required for sharp/wave ripple generation with oscillation patterns similar to *in vivo* observations. Our model has plausible values for single neuron, synapse and connectivity parameters, random connectivity and no strong feedforward drive to the inhibitory population. Specifically, whereas temporally broad excitation can lead to high-frequency oscillations in the ripple range, sparse pyramidal cell activity is only obtained with pulse-like external CA3 excitation. Further simulations indicate that such short pulses could originate from dendritic spikes in the apical or basal dendrites of CA1 pyramidal cells, which are triggered by coincident spike arrivals from hippocampal region CA3. Finally we show that replay of sequences by pyramidal neurons and ripple oscillations can arise intrinsically in CA1 due to structured connectivity that gives rise to alternating excitatory pulse and inhibitory gap coding; the latter denotes phases of silence in specific basket cell groups, which induce selective disinhibition of groups of pyramidal neurons. This general mechanism for sequence generation leads to sparse pyramidal cell and dense basket cell spiking, does not rely on synfire chain-like feedforward excitation and may be relevant for other brain regions as well.

## Author summary

During certain phases of sleep, rest and consummatory behavior the hippocampus brain area generates strong high frequency oscillations. These oscillations are important for memory formation and consolidation. To date, the mechanisms underlying their generation remain incompletely understood. We find that in unstructured networks, carefully designing how excitation is transmitted within the hippocampus is required for the generation of robust fast oscillations. Broad, temporally extended excitation of cells results in unrealistic single cell activity, whereas temporally narrow input that differs from cell to cell gives rise to oscillations with realistic single cell and network activity. We show that the biophysical mechanism to generate the required temporally narrow excitation may be related to spiking events in the dendrites, which are triggered by coincident input. Our results in structured networks suggest that the interplay of hippocampal excitation and inhibition can serve as a means to generate robust sequential activity, which is thought to be crucial for memory formation and recall. The sequence generation mechanism also leads to strong high frequency oscillations with sparse excitatory cell and frequent inhibitory cell spiking, as observed in the hippocampus.

## Introduction

Hippocampal sharp wave/ripples (SPW/Rs) are remarkable both from a neurophysiological viewpoint and in their behavioral impact: On the one hand, they consist of strong increases of spiking activity in large parts of local neuron populations (the sharp wave) together with oscillatory, extraordinarily coherent neuronal discharges (ripples). On the other hand, SPW/Rs have directly been shown to be important for memory consolidation [1–6] and might be involved in planning of future actions [1, 7]. Perhaps the most striking signature of memory consolidation is the phenomenon of hippocampal replay (see [8] for a recent review). During replay events co-occurring with SPW/R complexes, sequences of pyramidal cell action potentials encoding location, so-called place cells [9], are repeated on a faster timescale.

SPW/Rs are most prominently observed in the hippocampal region CA1. The main source of external excitation arriving in CA1 during a SPW/R event is a sharp, wave-like depolarization from area CA3 delivered via the Schaffer collaterals (SCs) to the basal and proximal apical dendrites of CA1 pyramidal cells [1, 10–12]. This input likely generates the sharp wave of spiking activity in CA1. We note that in *in vitro* experiments, SPW/Rs can also be generated in the isolated region CA1 [13–15]. A typical sharp wave has a duration of 40 − 100 ms [1]. The co-occurring ripple oscillations were first observed as oscillations in the local field potential (LFP) recorded in the CA1 stratum pyramidale [10, 11, 16]. Direct measurements of spiking activity and modeling studies show that these LFP ripples are mainly caused by the temporally precise, sparse firing of excitatory pyramidal cells [17–19], possibly with a smaller contribution from interneuronal activity [1, 19].

The onmodulated CA1 ripples are uncorrelated with weaker, lower frequency ripples occurring in CA3 and are thus considered to be generated by CA1 networks [11, 20]. How CA1 generates the fast ripple oscillation is to date not clear; the focus of this article is to design minimal CA1 network models that incorporate plausible CA1 connectivity and produce SPW/Rs with realistic network and single-cell activity.

Several classes of distinct models exist for ripple generation: interneuron ripples, pyramidal interneuron ripples and pyramidal ripples (see also Discussion for an overview figure). Each model class puts a different emphasis on the contributions of excitatory (E) and inhibitory (I) neurons. The first class, which we term *interneuron ripples*, (narrowing down the definition of inhibition-first ripples given in [21]) are models where mainly the inhibitory neurons generate the rhythm and entrain the excitatory ones. The standard, network model of this class (*interneuron network ripples*) posits that the ripples in CA1 are generated by tonic excitation of a recurrent network of fast-spiking basket cells (BCs) of the parvalbumin-positive (PV+) immunoreactive type [21–28]. The oscillation unfolds according to a mechanism also thought to underlie certain gamma oscillations (interneuron network gamma, ING) [29–33]. In support of this mechanism, theoretical studies have shown that fast oscillations in the ripple range can be generated easily in the presence of short synaptic time constants for the recurrent GABAergic inhibitory currents [23, 34], both in integrate-and-fire (IF) [34] and more detailed Hodgkin-Huxley conductance-based neuron models [23]. Such models have recently been successfully applied to explain phenomena such as intraripple frequency accommodation and the dependency of the network frequency on GABA modulators [21]. Another model that falls within the interneuron ripple class proposes that a single neuron mechanism underlies the ripple rhythm: calcium spikes in the dendrites of PV+BCs generate high-frequency oscillations in the membrane potential and output action potentials occur preferentially at the oscillation peaks [35].

Recently it has become clear, however, that an interneuron ripple model may be an insufficient description of the ripple mechanism. *In vivo* experiments [19, 36] suggest a crucial involvement of the local pyramidal E cell population in ripple generation. It was shown that optogenetic excitation of a small number of E cells in CA1 can generate high-frequency (ripple-like) oscillations (HFOs) and prolongs SPW/R events. Simultaneous optogenetic activation of I neurons was not necessary. Further, optogenetic activation of inhibitory interneurons did not generate LFP ripples and stopped them during SPW/Rs events; some coherent spiking activity in the ripple range could, however, be generated. I cell spiking caused by local excitatory-to-inhibitory connections was necessary for the ripple oscillations. It was thus proposed that interactions between E and I cell populations after external excitation of E cells are both necessary and sufficient to generate ripple oscillations *in vivo* [19]. (In contrast, ref. [25] found in CA3 slices that GABAergic interneurons are both necessary and sufficient to generate ripple oscillations in the LFP, even when excitation is blocked.) These results suggest for CA1 a second class of models, which are the focus of the present article and which may be called *pyramidal interneuron ripples*. In them, both populations are similarly important for rhythm generation. In the standard, network model of this class (*pyramidal interneuron network ripples*), the ripple oscillation is generated by an interplay of the two recurrently connected E and I cell populations, where the E population receives external drive. The excitatory-inhibitory loop then generates an oscillation according to a mechanism that is called pyramidal interneuron network gamma (PING) in the context of gamma oscillations [30–33]: The excitatory population excites the inhibitory population, which in turn transiently reduces E cell activity. After enough excitation has built up in the E cell network, the cycle restarts. If the interneuron network alone can already oscillate due to its recurrent inhibition, adding an excitatory population yields, for weak synchrony, a compromise between the oscillations that emerge due to the two mechanisms [23, 34] or, for strong synchrony, competition between them [33, 37]. Networks of the former, weakly synchronized type have been suggested as model for ripple oscillations [34, 38]. Adding the excitatory population usually decreases the oscillation frequency of the network, but can also increase it [23, 34].

In the last class of models, which we term *pyramidal ripple* models (equivalent to the excitation-first class in [21]), the excitatory neuron population mainly governs the ripple rhythm [39–42] and entrains the inhibitory neurons by local excitatory-to-inhibitory synaptic connections. The presence of inhibition can nevertheless be crucial, as it may sharpen the ripples and prevent unrhythmic pathological spiking activity [41].

The historically first subclass of pyramidal ripple models posits that gap junctions between the axons of pyramidal cells enable spike propagation in the axonal plexus. Through antidromic invasion into the somata, these generate pulses of somatic spikes at ripple frequency [13, 15, 39, 40, 43, 44]. While there is no concluding, ultrastructural evidence for axo-axonic gap junctions between CA1 pyramidal cells [45], ref. [43] showed that axons of CA1 pyramidal neurons are dye coupled. Further, gap junctions have been ultrastructurally demonstrated between dendritic and somatic locations in CA1 pyramidal neurons [46] and between axons of granule cells of the dentate gyrus [47]. Experiments, however, indicate that somatic spikes are generated by orthodromic excitation during SPW/Rs *in vivo* [48].

The second subclass of pyramidal ripple models proposes that synchrony propagation supported by nonlinear dendrites underlies SPW/Rs [41, 42, 49–53]. Specifically, it assumes that spikes in the basal dendrites of CA1 pyramidal neurons are generated by sufficiently synchronous recurrent excitation [41, 42]. Whether the recurrent excitatory connectivity in CA1, which is very sparse (typical values for coupling probabilities are between 1 and 2% [54, 55]), suffices to generate these dendritic spikes, is currently unclear. Models incorporating such non-linearities [41, 42] have successfully reproduced many experimental findings, including sparse firing of pyramidal cells, different CA1 and CA3 ripple frequencies and the phase relation of the excitatory and inhibitory cell population activity.

A successful model for CA1 ripple generation must reproduce the main properties of ripple oscillations in CA1. The most important criterion is that the activity of E and I cell populations should be modulated at a frequency in the ripple range, between 140 and 200 Hz. *In vivo* CA1 ripples lie in the lower (slow wave sleep ripples) and middle (awake ripples) part of this range [1, 17, 19, 56], *in vitro* ripples in the upper part. A model for *in vivo* and *in vitro* ripples must therefore be able to cover the entire frequency range [1, 13, 15].

The second main property of ripple oscillations is that pyramidal neurons fire one to two orders of magnitude less frequently than interneurons [17, 57]. During SPW/Rs, pyramidal cells have firing rates between 0 and 10 Hz, with a bias towards smaller frequencies, so that the mean firing rate during ripples is between 1 and 2 Hz [58]. More concretely, pyramidal cells tend to fire only once during a sharp wave/ripple event [17, 19, 36], which is composed of multiple ripple waves (or: ripple cycles). This entails that different sets of pyramidal neurons are active on each ripple wave. Fast-spiking PV+BCs [59] have much higher average firing rates and often contribute a spike to every ripple wave [10, 17, 36, 60, 61], or even a doublet of spikes [62]. Their firing rates during SPW/Rs typically lie between 10 and 200 Hz [17, 57, 63]. 10 − 20% of pyramidal cells typically discharge during an SPW/R event, whereas 80% and more of interneurons fire [17, 19, 64, 65].

In this paper, we first observe that simply augmenting previous inhibition-first models by adding pyramidal neurons is not sufficient to generate realistic single-cell and network dynamics. We show that nevertheless the generation of high-frequency oscillations in the right frequency range with realistic single-neuron dynamics is possible with random two-population models if excitation to CA1 pyramidal cells is delivered in an inhomogeneous, temporally narrow, fashion. Such inhomogeneity could be brought about by dendritic spikes depolarizing the soma of CA1 pyramidal cells, which are mediated by coincidence detection from incoming CA3 spikes. Finally, we show that in structured two-population models robust sequences of pyramidal cell firing activity and ripple oscillations, as observed during hippocampal replay, can be generated intrinsically in CA1 by a mechanism involving disinhibition of selected groups of pyramidal cells.

## Materials and methods

**Fig 1:**
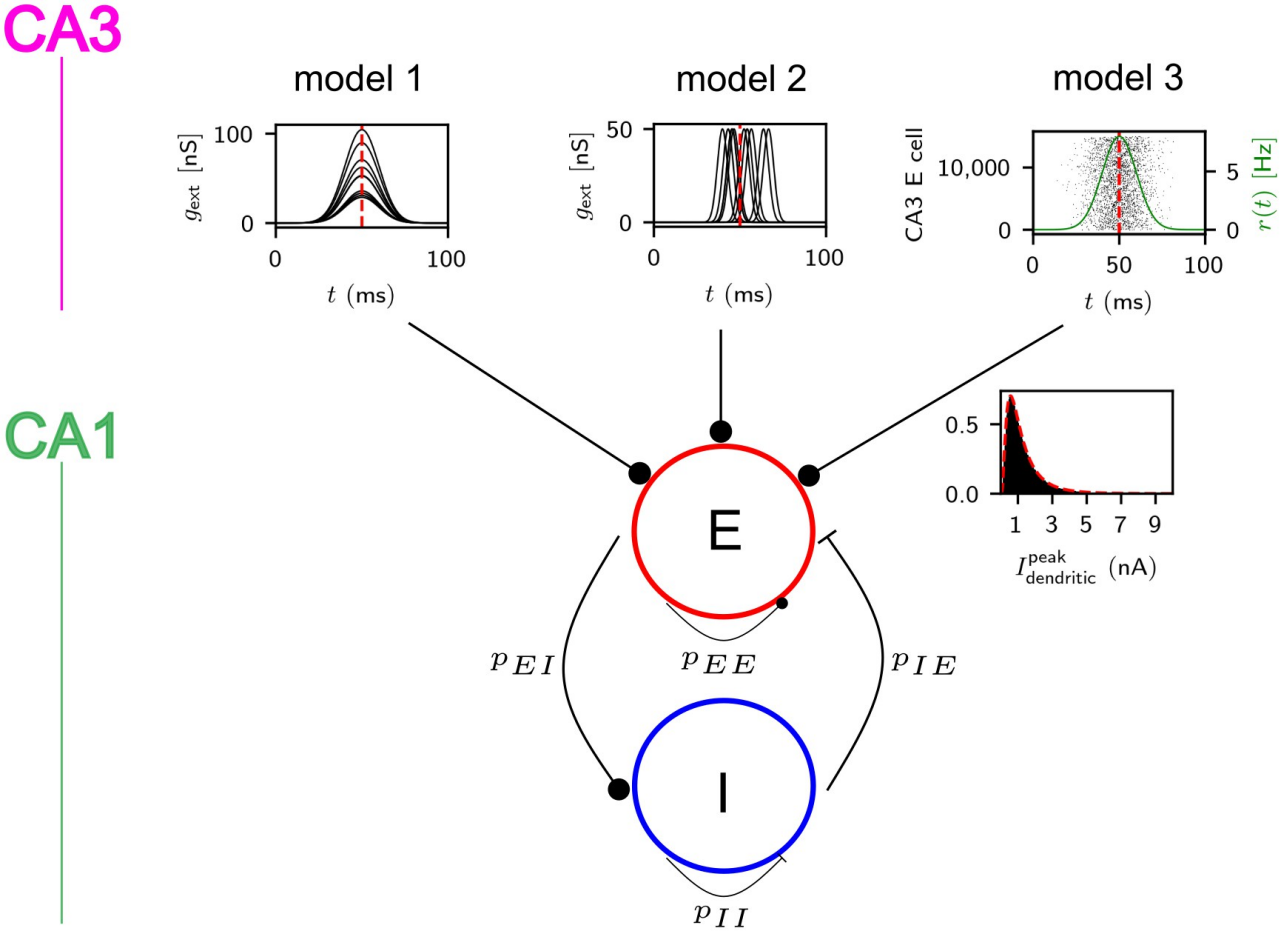
Model overview. The models consist of a population of *N*_*E*_ excitatory and a population of *N*_*I*_ inhibitory CA1 neurons, which are connected with probabilities *p*_*αβ*_, *α*, *β* ∈ {*E*, *I*} (lower panel) and receive different external input from CA3 (upper panel): In model 1 and 2, it is represented by the excitatory conductances *g*_ext_ (Eq 6) that it evokes in *n*_*E*_ CA1 neurons. In model 1, the time courses of these conductances are broad and for each neuron centered around the same time (upper left panel). In model 2, the time courses are short, pulse-like and centered around different times (upper middle panel). In model 3, CA3 is represented by a population of input neurons. Each such neuron fires according to an inhomogeneous Poisson process with Gaussian rate profile (upper right panel, rate time course (Eq 7, green) is overlaid on spike rastergram). Spike transmission from CA3 to CA1 is then filtered by a strong apical or basal dendrite of the receiving CA1 neuron: Besides the linear input transmission, sufficiently coincident input evokes dendritic spikes. These generate somatic currents whose peak strengths follow a lognormal distribution (the inset shows such a distribution together with its density given by Eq 2 (red dashed)) across neurons.

We consider three variants, model 1, 2 and 3, of a two-population model consisting of *N*_*E*_ excitatory pyramidal cells (PCs), the principal cells of hippocampal region CA1, and *N*_*I*_ inhibitory parvalbumin-positive BCs (Fig 1). For their implementation we use custom scripts in brian2 [66].

We model single neurons as conductance-based leaky integrate-and-fire neurons, since we assume that the SPW/Rs are a network dynamical pattern that does not crucially depend on the details of subthreshold dynamics and spike generation. The dynamics of a single neuron in the excitatory population are governed by the equation

$$\begin{array}{c} C_{E}\frac{dV(t)}{dt}=I_{\text{leak}}^{E}+I_{\text{exc}}^{E}+I_{\text{inh}}^{E}+I_{\text{dendritic}}\left(t\right)+I_{\text{noise}}^{E}\left(t\right)\;. \end{array}$$ (1)

The leak current is given by $I_{\text{leak}}^{E}=g_{l}^{E}(E_{\text{rest}}^{E}-V\left(t\right))$ and the excitatory and inhibitory currents are given by $I_{\text{exc}}^{E}=g_{\text{tot}}^{E}\left(t\right)(E_{\text{exc}}^{E}-V\left(t\right))$ and $I_{\text{inh}}^{E}=g_{\text{inh}}^{E}\left(t\right)\left(E_{\text{inh}}^{E}-V\left(t\right)\right)$, respectively. The total excitatory conductance consists of two parts: $g_{\text{tot}}^{E}\left(t\right)=g_{\text{exc}}^{E}\left(t\right)+\gamma g_{\text{ext}}\left(t\right)$, representing input from recurrent excitatory synapses and external input. The external input is a direct, not dendritically amplified, input present in model 1 and 2. It models input from CA3 or an optogenetic stimulation delivered in experiments [19]. *γ* ∈ {0, 1} determines whether this drive is present in the model, i.e. *γ* = 1 in models 1 and 2, whereas *γ* = 0 in model 3. The noise current $I_{\text{noise}}^{E}=\sigma_{n}\sqrt{2g_{l}^{E}C_{E}}\xi \left(t\right)$ is a Gaussian white noise input, independent between neurons and with strength *σ*_*n*_ = 1 mV.

*I*_dendritic_ is a somatic current triggered by apical or basal dendritic spikes and only present in model 3. We implement the generation of dendritic spikes via a temporal coincidence detection mechanism [41, 42]: As soon as the dendrite receives sufficiently many spikes from its presynaptic CA3 neurons within the dendritic integration window *w*_*D*_ = 2 ms (< 3 ms [67, 68]), a fast and often strong, but not necessarily suprathreshold excitatory current *I*_dendritic_ is generated. It depolarizes the somatic membrane potential a delay *τ*_*D*_ = 2 ms after the coincidence was detected. There is a refractory period *t*_*r*,*D*_ = 5.0 ms during which no further dendritic spikes are triggered—this means that after a dendritic spike, there cannot be another one for a duration of *t*_*r*,*D*_. We checked that our results do not depend critically on the exact value of *t*_*r*,*D*_ by performing simulations with smaller (*t*_*r*,*D*_ = 2 ms) and larger (*t*_*r*,*D*_ = 10 ms) values. The results that we will report below for model 3 remained qualitatively unchanged, only the parameters of the lognormal distribution (Eq 2) where HFOs in the ripple range occur shifted such that a larger (smaller) *t*_*r*,*D*_ was compensated for by an increase (decrease) in *μ* and/or *σ*.

We assume that every pyramidal neuron has one dendritic compartment where it receives the relevant input from CA3. This models the single main apical dendrite in CA1 pyramidal neurons [12, 67, 69] and its several oblique ones emanating from it. We do not explicitly model the compartment in this study, but instead focus on the impact a dendritic spike has on the somatic membrane voltage. All incorporated inputs from CA3 contribute in the same way to dendritic spike generation. In particular we do not separately model local effects such as the generation of weak dendritic spikes in the oblique dendrites [70]. Because innervation by CA3 Schaffer collaterals occurs on both basal and apical dendrites of CA1 pyramidal neurons [12], the modeled dendrite may alternatively be interpreted as an influential, privileged basal one [71] (neglecting the contribution of recurrent CA1 excitatory inputs to dendritic spike generation in such a dendrite—dendritic spikes in our model 3 are generated by feedforward Schaffer collateral input). Experiments show that the impact of dendritic spikes on the soma can be different depending on the generating dendrite, but also on the depolarization and the somatic firing history [67, 72, 73]. If not mentioned otherwise we thus assume that the strengths of dendritic spikes across neurons have a lognormal distribution as experimentally found for several other neuronal properties [58, 74]. Specifically the peaks of the currents induced in the soma have a lognormal distribution across neurons,

$$\begin{array}{c} \frac{I_{\text{dendritic}}^{\text{peak}}}{\text{nA}}∼\text{lognormal}\left(\mu ,\sigma \right)\;. \end{array}$$ (2)

**Fig 2:**
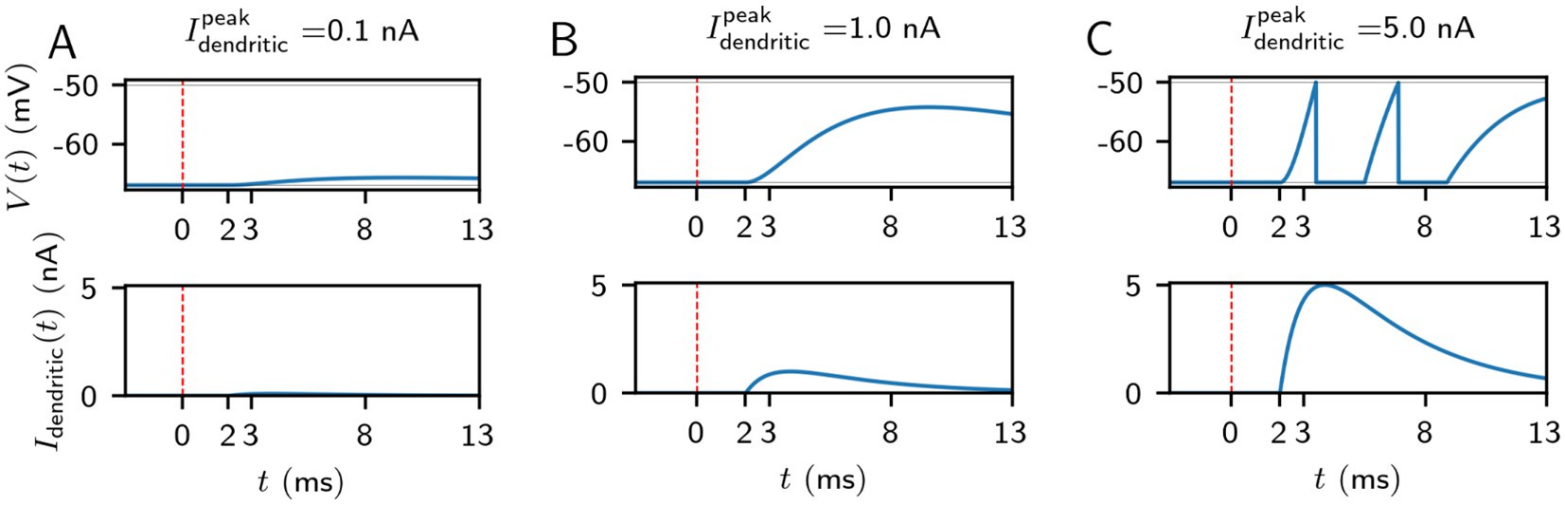
The impact of dendritic spikes on the soma. A dendritic spike is generated at *t* = 0 ms, marked by the red dashed vertical line. It is modeled by the dendritic current *I*_dendritic_ (lower subpanels in A,B,C) arriving at the soma *τ*_*D*_ = 2 ms later (here at 2 ms). For small peak dendritic currents ($I_{\text{dendritic}}^{\text{peak}}=0.1\;\text{nA}$, panel A), the somatic depolarization (upper subpanel) is small (here: 1.28 mV) when starting at rest. For larger peak dendritic currents, the somatic depolarization increases (12.78 mV for $I_{\text{dendritic}}^{\text{peak}}=1\;\text{nA}$, panel B), but remains subthreshold. For $I_{\text{dendritic}}^{\text{peak}}\geq 1.34\;\text{nA}$, at least one somatic spike is generated; for $I_{\text{dendritic}}^{\text{peak}}=5\;\text{nA}$ (panel C), two spikes are generated and there is significant depolarization visible in the membrane voltage even after the second spike. The scale for the bottom panels is fixed to facilitate comparison between the different values for the dendritic peak current.

*μ* and *σ* are dimensionless as the samples are drawn from a lognormal distribution. The current in the soma induced by the dendritic spike has a rise time of 1 ms and a decay time of 4 ms [67]. If *V* starts at its resting potential and noise and further input are absent (*σ*_*n*_ = 0 in Eq 1), the minimal peak current to generate a somatic action potential is approximately 1.34 nA. Stronger dendritic spikes can generate multiple somatic spikes: without further inputs and if its peak current is at 10 nA, a dendritic spike generates four somatic ones. However, more than 99.8% of the dendrites in our model 3 simulations without replay have a smaller impact for representative values of *μ* = 0 and *σ* = 0.75 (see Results). This follows from the cumulative density function $\text{CDF}\left(x;\mu ,\sigma \right)=\frac{1+\text{erf}(\frac{\text{ln}x-\mu }{\sqrt{2}\sigma })}{2}$ of the lognormal distribution, which yields CDF(*x*; *μ*, *σ*) = 0.9983 with *x* = 10, *μ* = 0 and *σ* = 0.75. We note that somatic action potential bursts in CA1 pyramidal cells are usually generated by a more involved mechanism, where calcium spikes in the apical tuft are generated with the support of backpropagating action potentials [75, 76]. Fig 2 shows examples of dendritic currents and the associated somatic membrane voltage time courses in our model.

The dynamics of the inhibitory neurons are given by

$$\begin{array}{c} C_{I}\frac{dV(t)}{dt}=I_{\text{leak}}^{I}+I_{\text{exc}}^{I}+I_{\text{inh}}^{I}+I_{\text{noise}}^{I}\left(t\right), \end{array}$$ (3)

similar to Eq 1. The leak, excitatory and inhibitory currents are $I_{\text{leak}}^{I}=g_{l}^{I}(E_{\text{rest}}^{I}-V\left(t\right)),I_{\text{exc}}^{I}=g_{\text{exc}}^{I}\left(t\right)(E_{\text{exc}}^{I}-V\left(t\right))$ and $I_{\text{inh}}^{I}=g_{\text{inh}}^{I}\left(t\right)\left(E_{\text{inh}}^{I}-V\left(t\right)\right)$, respectively. $I_{\text{noise}}^{I}=\sigma_{n}\sqrt{2C_{I}g_{l}^{I}}\zeta \left(t\right)$ is a Gaussian white noise input, independent between neurons and with strength *σ*_*n*_ = 1 mV.

In both the E and I populations, once a neuron reaches its firing threshold $V_{\text{thresh}}^{\alpha }$ (*α* ∈ {E, I}), it is reset to $V_{\text{reset}}^{\alpha }$ and remains at this potential during an absolute refractory period $t_{\text{ref}}^{\alpha }$. The neurons are connected by chemical synapses, see next section for details. We do not consider gap junction coupling.

We have chosen the single neuron and network parameters in agreement with neuroanatomical and neurophysiological experimental knowledge on the hippocampal area CA1. The data we refer to come mostly from rats, but also from mice [55]. These parameters (together with model-specific parameters and a justification for some single cell parameters) can be found in S1 Appendix.

### Synaptic dynamics and connectivity

#### Dynamics

The excitatory and inhibitory synaptic conductances induced by a presynaptic spike at time *t* = 0 are given by a bi-exponential function,

$$\begin{array}{c} g_{\beta }^{\alpha }\left(t\right)=g_{\beta ,\text{peak}}^{\alpha }s_{\beta }^{\alpha }\;(\text{exp}\;(-\frac{t-\tau_{l}}{\tau_{\beta ,d}^{\alpha }})-\text{exp}\;(-\frac{t-\tau_{l}}{\tau_{\beta ,r}^{\alpha }}))\;, \end{array}$$ (4)

for *t* ≥ *τ*_*l*_, where *τ*_*l*_ is the transmission delay, and $g_{\beta }^{\alpha }\left(t\right)=0$ otherwise. $s_{\beta }^{\alpha }$ is a constant ensuring that $g_{\beta }^{\alpha }$ has its maximum at $g_{\beta ,\text{peak}}^{\alpha }$. Suppressing the indices *α* and *β* we have

$$\begin{array}{c} \frac{1}{s}={\left(\frac{\tau_{r}}{\tau_{d}}\right)}^{\frac{\tau_{r}}{\tau_{d}-\tau_{r}}}-{\left(\frac{\tau_{r}}{\tau_{d}}\right)}^{\frac{\tau_{d}}{\tau_{d}-\tau_{r}}}\;. \end{array}$$ (5)

This setup is similar to that used in [42] and [21]. We show time courses for postsynaptic potentials, conductances and postsynaptic currents in S1 Fig.

#### Connectivity

In our unstructured networks, the two neuronal populations are coupled uniformly at random, with probabilities *p*_*αβ*_, where *α* ∈ {E, I} denotes the presynaptic population and *β* ∈ {E, I} denotes the postsynaptic population. If not mentioned otherwise, we set *p*_*EE*_ = 0.0164, *p*_*II*_ = 0.2, *p*_*IE*_ = 0.1 and *p*_*EI*_ = 0.1. In the following, we explain how we arrive at these specific values. To start, we motivate our choice of *N*_*E*_ and *N*_*I*_, the number of E and I neurons in our models, which we usually choose to be *N*_*E*_ = 12000 and *N*_*I*_ = 200. This approximately agrees with the neuron numbers in a typical CA1 slice, of thickness 0.4 mm and a volume of 0.057 mm^3^ [21]. The numbers respect the ratio of pyramidal cells to PV+BCs in rat CA1, which is approximately 60:1 [55]. To compute the connection probabilities *p*_*αβ*_, we assume that all connections between E and I cells are realized in the subset of CA1 neurons we consider. This means that we first determine how many inputs each E or I cell receives from the other cells in the population and then compute the connection probabilities using the numbers for *N*_*E*_ and *N*_*I*_ given above.

A hallmark of CA1 is its very sparse recurrent excitatory connectivity [54, 55]. Every pyramidal cell receives input from approximately 197 other pyramidal cells [55]. We set *p*_*EE*_ = 1.64% so that for *N*_*E*_ = 12000 we have *p*_*EE*_*N*_*E*_ = 197.

For the I-to-I connectivity, we set *p*_*II*_ = 0.2 [55, 77, 78], which, for *N*_*I*_ = 200, means that each basket cell on average receives *p*_*II*_*N*_*I*_ = 40 synapses from other PV+BCs in the network. This value is obtained as follows: a single PV+BC contacts on average 64 other PV+ cells [77], of which 60% are basket cells [55], such that a single PV+BC contacts 38, approximately 40, other PV+BCs on average [21].

For the I-to-E synapses, we obtain several estimates, (i) based on the experimentally observed number of PCs that are postsynaptic to a single PV+BC and (ii) based on the experimentally observed number of PV+BCs that are presynaptic to a single PC. We first consider ref. [77]: (i) The conducted experiments in CA1 found that basket cells innervate between *D* = 1500 and *D* = 2000 pyramidal cells each. For *D* = 1500 we obtain, using the assumption of homogeneous and random connectivity, 1500 = *p*_*IE*_*N*_*E*_ and thus *p*_*IE*_ = 0.125. Analogously, *D* = 2000 yields *p*_*IE*_ = 0.167. (ii) Ref. [77] further showed that 30 − 40 PV+ cells, of which 60% are PV+BCs [55], converge on a single CA1 PC. Thus, a single PC should be contacted by 18 ≤ *C* ≤ 24 PV+BCs. Under the assumption of homogeneous random connectivity, the mean number of PV+BCs contacting a single PC is given by *C* = *p*_*IE*_*N*_*I*_, which, with the values for *p*_*IE*_ obtained using estimate (i) above (0.125 ≤ *p*_*IE*_ ≤ 0.167), would result in 25 ≤ *C* ≤ 34. This is slightly too large in comparison to the experimental values from estimate (ii). Therefore, a lower value of *p*_*IE*_ = 0.1 seems also plausible. The meta-study [55] allows to obtain similar estimates: (i) It was found that each PV+BC contacts on average 943 PCs. This again fixes *p*_*IE*_ because 943 = *p*_*IE*_*N*_*E*_, yielding *p*_*IE*_ = 0.079. (ii) Ref. [55] further found that each pyramidal cell is innervated by 17 PV+BCs, such that 17 = *p*_*IE*_*N*_*I*_, resulting in a value of *p*_*IE*_ = 0.085, which is also close to 0.1. We thus fix *p*_*IE*_ = 0.1 and consider values in the range from 0.08 to 0.17 as biologically plausible.

We first determine *p*_*EI*_ for the E-to-I connectivity in an approach analogous to (i) above. We use the experimental result that each CA1 PC innervates 91 interneurons, of which approximately 14% are PV+BCs [55]. Therefore each PC diverges to innervate approximately *D* = 13 PV+BCs (*D* is chosen in analogy to estimate (i) for the I-to-E connectivity above). Thus, *D* = *p*_*EI*_*N*_*I*_, so that *p*_*EI*_ = 6.5%. When trying to obtain the E-to-I connectivity in analogy to the approach (ii) above, one first notes that the number of excitatory synapses on a PV+BC is not known [55]. It has, however, been estimated that a hypothetical ‘average interneuron’ in CA1 receives 2211 excitatory boutons from local collaterals (i.e. local CA1 PCs) [55]. If each CA1 PC makes on average 3 synapses onto each postsynaptic interneuron [55], this means that each interneuron is contacted by approximately *C* ≈ 740 CA1 PCs. This results in $p_{EI}=\frac{740}{N_{E}}=0.062$. If each CA1 PC made only two synapses onto each postsynaptic interneuron, this would result in *C* ≈ 1106 and thus $p_{EI}=\frac{1106}{N_{E}}=0.092$. We therefore consider values 6.5% ≤ *p*_*E*_ ≤ 10% as biologically plausible. For concreteness, we fix *p*_*EI*_ = 0.1 in this study. This is at the upper end of the biologically plausible values, but acceptable because we will systematically vary the excitatory drive to the E population in our simulations: Decreasing *p*_*EI*_ is similar to decreasing the number of active E cells since the latter reduces the number of the realized E-to-I connections that will have a postsynaptic effect. A decrease in the number of active E cells results in our models from changing the number *n*_*E*_ of PCs that receive CA3 drive in model 1 and 2 and by changing the strength of the CA3 drive in model 3, see next section.

In summary, using the values available in the experimental literature [55, 77, 79–82] we obtained for our networks *p*_*EI*_ = 0.1, so that each PV+BC receives on average input from 1200 PCs, and *p*_*IE*_ = 0.1, so that each PC receives on average input from 20 PV+BCs. These connection probabilities are in good agreement with many previous computational studies of CA1 [33, 41, 42]. Notably, [21] use the same values for the connection probabilities except for a higher value of *p*_*IE*_ = 0.3. Our more detailed choice for the connection probabilities differs from that in ref. 34, where a single value of *p* = 0.2 for all synapses was assumed. Finally, it differs from the all-to-all connectivity for all synapses except the E-to-E synapses assumed by [26].

To conclude, as a result of the considerations in this section we set *p*_*EE*_ = 0.0164, *p*_*II*_ = 0.2, *p*_*IE*_ = 0.1 and *p*_*EI*_ = 0.1 (if not mentioned otherwise).

### Excitation of pyramidal cells by CA3

#### Models 1 and 2

Models 1 and 2 explicitly mimic sharp-wave input from CA3 without modeling CA3 [21]: a subset *n*_*E*_ of the pyramidal cells is driven by time-dependent conductances *g*_ext_ with a Gaussian profile,

$$\begin{array}{c} g_{\text{ext}}\left(t\right)=g_{\text{ext}}^{0}\;\text{exp}\;(-\frac{{\left(t-t_{0}\right)}^{2}}{2\sigma_{g}^{2}})\;. \end{array}$$ (6)

#### Model 3

In model 3, CA3 is modeled as a population of *N*_*E*,CA3_ = 15000 excitatory neurons that each spike according to an inhomogeneous Poisson process with rate time course

$$\begin{array}{c} r\left(t\right)=r_{0}\;\text{exp}\;(-\frac{{\left(t-t_{0}\right)}^{2}}{2\sigma_{t}^{2}})\;. \end{array}$$ (7)

The connection probability between CA3 and CA1 is $p=\frac{130}{N_{E,\text{CA3}}}\approx 1\%$ if not mentioned otherwise. This means that every CA1 pyramidal neuron on average receives input connections from 130 CA3 pyramidal neurons. The number is comparable to the typical number of active presynaptic CA3 neurons of a CA1 PC during an SPW/R event (120 − 300 active inputs, [42, 83]). This is much lower than the number of CA3 neurons converging on a single CA1 pyramidal neuron (15000 − 30000, [55]). However, only approximately 1% of all CA3 pyramidal neurons are active during a SPW/R [57, 64]. Assuming that rat CA3 contains approximately 205000 PCs [55] and that the average connection probability from CA3 to CA1 (neglecting CA3 sublayer specificity) is between 1 and 8% [55], we obtain between 0.01 ⋅ 0.01 ⋅ 205000 ≈ 21 and 0.01 ⋅ 0.08 ⋅ 205000 = 164 inputs per CA1 PC, which is consistent with our choice for *p* given above. In light of this estimate, we consider one hundred to a few hundreds, but not thousand, active inputs to a CA1 PC from CA3 to be realistic estimates. Our results do not depend crucially on the exact size of *p*. For example, a modest increase in *p* can be compensated for by a decrease in the average strengths of the peak dendritic currents (given by Eq 2) or the peak rate *r*_0_ in Eq 7, see S3 Appendix for an analytical computation. Importantly, in our model 3 not every CA3 cell is active, which reduces the average number of active CA3 inputs to a CA1 PC 5-fold from 130 to 26. This shall cover the fact that not all spikes interact nonlinearly, as they can arrive at different dendritic compartments (Discussion, S3 Appendix). As already mentioned above when introducing Eq 1, the impact of temporally coincident spikes from CA3 is nonlinearly amplified, in all *N*_*E*_ excitatory cells: if in one of these cells 5 or more spikes arrive in an interval of *w*_*D*_ = 2 ms [67, 68], a dendritic spike is triggered. We additionally require that at that time, the membrane voltage is above the inhibitory reversal potential $E_{\text{inh}}^{E}$. Due to the noise term in Eq 1, it can occasionally, but rarely, happen that *V* is slightly driven under the inhibitory reversal potential. Because dendritic spikes require that the soma is not strongly hyperpolarized [67, 68], we added this additional condition for the generation of dendritic spikes (see, however, [73] for the robustness of strong dendritic spikes against recurrent inhibition). We checked that the dynamics of model 3 are qualitatively unchanged without this additional condition.

Irrespective of the coincidence detection mechanism, each CA3 spike impinging on a CA1 PC also causes a small depolarization (rise/decay time 1/2 ms, peak conductance 0.75 nS) with a delay of 1 ms. The dendritic spike is incorporated in the model by the current *I*_dendritic_(*t*) that it generates in the soma (see Fig 2). We assume that the impact of the dendritic spike is stereotypical, i.e. $I_{\text{dendritic}}^{\text{peak}}$ (cf. Eq 2) is for a given neuron constant over time and independent of the CA3 inputs that triggered it. This can be motivated by the fact that dendritic spikes are stereotypical and couplings between dendrites and soma are reliable [73]. There are thus two sources of heterogeneity in the excitatory connections from CA3 to CA1. The first source is that the number of CA3 inputs impinging on a given CA1 cell is variable as only the connection probability *p* (see above) is fixed. The second source is the variable peak dendritic current $I_{\text{dendritic}}^{\text{peak}}$ (cf. Eq 2) which is drawn, for each CA1 PC independently, from a lognormal distribution (Eq 2) with fixed parameters *μ* and *σ*. In our model, not every dendritic spike causes a somatic spike or even has a discernible influence on the soma (see Fig 2), which is in agreement with experimental observations [73, 84].

Large and small amplitude fast dendritic spikes have been observed in the apical dendrites of CA1 PCs during SPW/Rs [84]. Large amplitude fast dendritic spikes lead slow dendritic spike components (see also [68, 70]) and ride on top of them. Slow dendritic spikes and related bursts of fast ones are not included in our model. Slow dendritic spikes, which are calcium and/or NMDA channel-dependent, are less sensitive to synchronous input and their generation can crucially involve backpropagating action potentials [70, 85–87]. This is a caveat, because it might indicate that dendritic spikes in [84] can be less sensitive to synchrony in the input from CA3 and their generation can involve other mechanisms such as backpropagating action potentials.

## Results

In the following, we study our three minimal CA1 two-population models (cf. Fig 1) with respect to the generation of HFOs in the ripple range. In each of the three models, we compute the following four quantities for the E and I population: the oscillation frequency of the population activity, its standard deviation across different realizations, the number of spikes per active cell and the fraction of active cells. The first quantities of interest are the frequencies of the network activity of the I and E population. We compute their means *f*_*α*_ (*α* ∈ {*E*, *I*}) and standard deviations std(*f*_*α*_) (*α* ∈ {*E*, *I*}) across at least 10 network simulations with independent random realizations of external noise and connectivity. A small standard deviation indicates that the oscillation generated by the network is ‘robust’. This is a desirable property because it indicates that the oscillations are hardly affected by the frozen noise caused by the random connectivity and the noise on the membrane voltage (cf. Eqs 1 and 3). In addition, to assess the firing activity of individual cells, we compute the mean number of spikes per active neuron, *C*_*α*_, and the fraction of active cells in the population, *q*_*α*_ (*α* ∈ {*E*, *I*}). This quantity is defined as the ratio of the number of cells which spiked at least once during the whole simulation and the total number of neurons *N*_*α*_ (*α* ∈ {*E*, *I*}). We chose these basic statistical measures because they allow for an easy comparison with experimental values. In particular, computing spike counts instead of rates is suitable for our short simulations.

### Temporally broad excitation of pyramidal cells (model 1)

We first study a model in which a subset of the PC population receives temporally broad input, which peaks at the same time *t*_0_, but has a different amplitude for each cell: A subset of *n*_*E*_ PCs is each excited by a time-dependent conductance (Eq 6), which peaks at *t*_0_ = 50 ms. The amplitudes $g_{\text{ext}}^{0}$ of the conductances are distributed according to a Gaussian with mean $\bar{g}$ and standard deviation ${\text{CV}}_{g}\bar{g}$, where CV_*g*_ is the coefficient of variation, which is kept constant when we vary $\bar{g}$. The setup is similar to the indirect drive condition in [21]. Following [19], where it was shown that exciting a small number of CA1 pyramidal cells is necessary and (in the presence of intact inhibition) also sufficient to induce a ripple oscillation, we first do not include any external excitatory drive to the inhibitory population of PV+BCs. That inhibition is necessary ([19]) suggests a model of rhythmogenesis where the excited pyramidal cell subpopulation excites inhibitory cells, which then inhibit their excitatory targets. In such a model, the network frequency will depend on the recurrent E-to-I and I-to-E connections [34].

**Fig 3:**
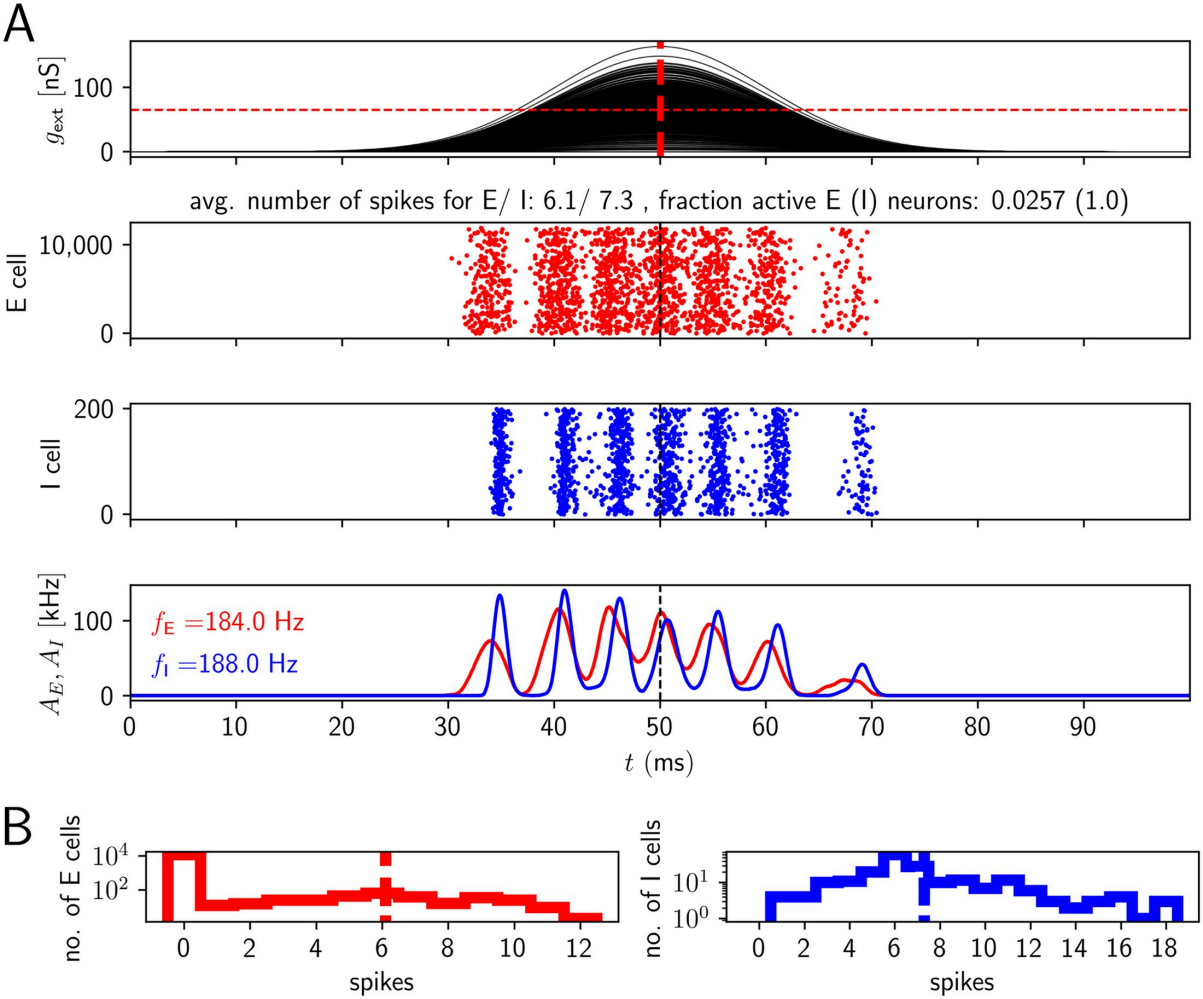
Network activity for temporally broad excitation of E cells (model 1). The population activity of E and I cells oscillates at ripple frequency, but the active E cells contribute many spikes to a SPW/R event, like I cells. A, upper subpanel: Time courses of sharp wave input to the CA1 E cells (input conductances). The horizontal red dashed line indicates the mean $\bar{g}$ of the sharp wave amplitudes. Middle: spike rastergrams of the CA1 E and I cells (red and blue). Lower: population rates (red: population of E cells, blue: population of I cells). *f*_*E*_ and *f*_*I*_ in the lower panel give the oscillation frequency of the E and I population (spectrogram peak). B: Histograms of spike counts for E (left) and I (right) cells on a logarithmic scale. Dashed vertical lines highlight the average spike count. Parameter values as in Fig 4, $\left(\bar{g},n_{E}\right)=\left(65\;\text{nS},400\right)$.

**Fig 4:**
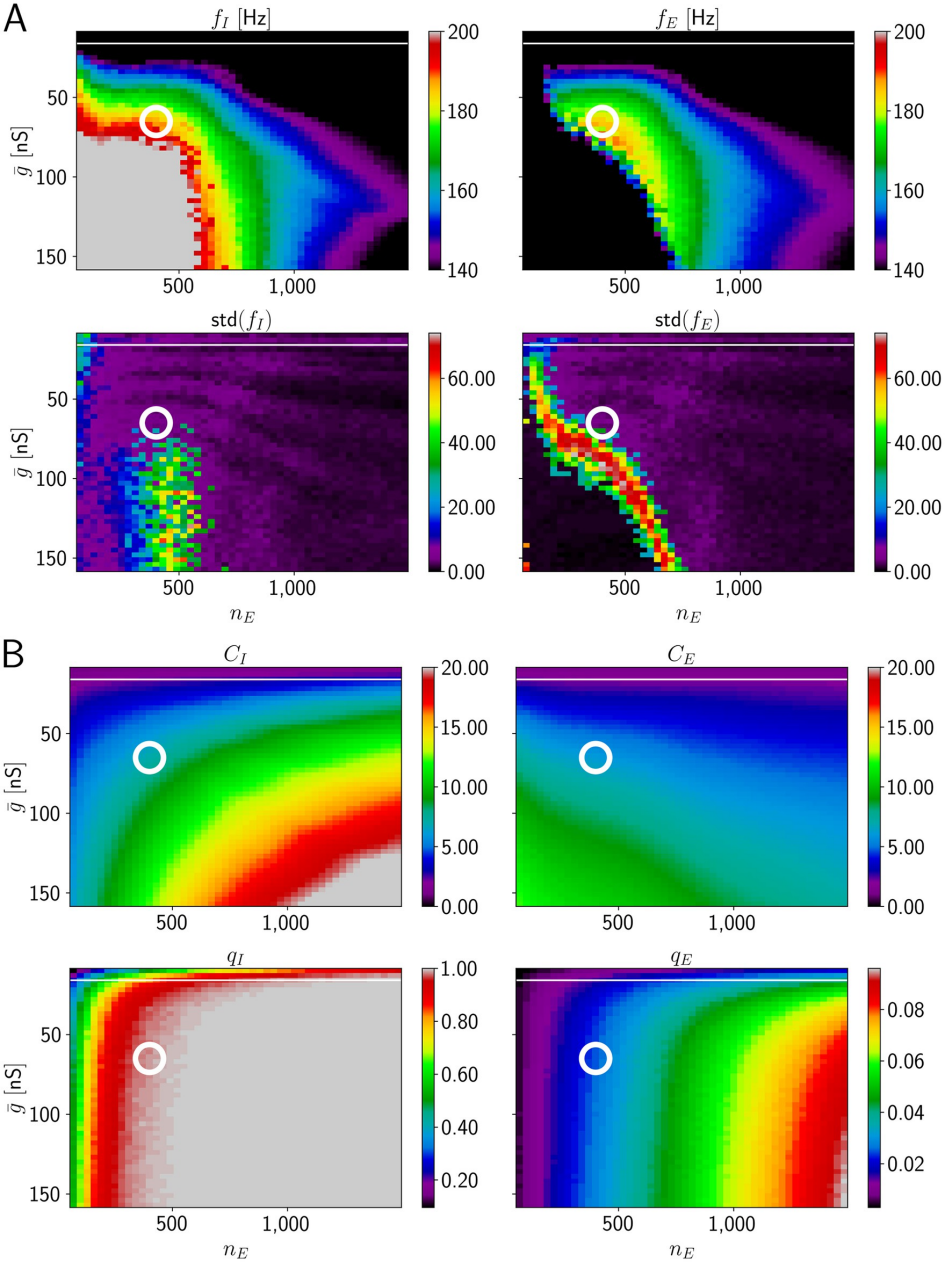
HFOs in networks with temporally broad excitation of E cells (model 1). There is no overlap of the parameter region with reliable ripple oscillation generation and sparse spiking of E neurons. A, upper subpanel: Frequency of I and E population activity oscillations *f*_*I*_, *f*_*E*_ as a function of mean drive $\bar{g}$ and number of excited E cells *n*_*E*_ (average taken across different network realizations). Lower: Corresponding standard deviations across network realizations, std(*f*_*I*_) and std(*f*_*E*_). B, upper subpanels: mean number of spikes per active inhibitory (excitatory) neuron, *C*_*I*_ (*C*_*E*_). Lower: fraction of active neurons (*q*_*I*_ and *q*_*E*_). The range for *f*_*I*_ and *f*_*E*_ displayed in detail by different colors is the ripple range, [140, 200] Hz. The range for both *C*_*I*_ and *C*_*E*_ is [0, 20]. Frequency values below (above) this range are indicated in black (grey). To guide the eye, the white horizontal line indicates a border of the range of biologically plausible *C*_*E*_; this range lies completely above the line, which is at $\bar{g}=16\;\text{nS}$. The white circle indicates $\left(\bar{g},n_{E}\right)=\left(65\;\text{nS},400\right)$, the parameters used in Fig 3. Standard deviation of the sharp wave peak conductance is CV_*g*_ = 0.5, width of sharp waves *σ*_*g*_ = 10 ms.

To gain intuition, in Fig 3, we show network activities and histograms of spike counts for the two populations for a fixed level of excitatory drive and a fixed number of excited E cells ($\left(\bar{g},n_{E}\right)=\left(65\;\text{nS},400\right)$). We see that E cells that are active tend to be active on multiple ripple waves (i.e. ripple cycles). This is because high external input amplitudes remain high over multiple ripple waves. Active E cells spike considerably more often than once or twice (on average they spike approximately 6 times in the event of Fig 3, see also Fig 4B for the corresponding average over events *C*_*E*_). The spikes do not all occur on the same ripple wave: The absolute refractory period for a single E cell is 2 ms. A single ripple wave (broad red stripes in Fig 3) lasts less than 5 ms. It is therefore impossible that an E cell spikes 6 times in a single cycle (ripple wave): the shortest time interval (assuming instant spike generation after the end of each refractory period) separating 6 spikes would be 5 times the absolute refractory period, that is, 10 ms, which is longer than a single ripple wave.

I cells basically spike on every ripple wave (on average ∼ 7 times in Fig 3). In particular, nearly all I cells are active, *q*_*I*_ ≈ 1. Most E cells, in contrast, remain silent (because *n*_*E*_ ≪ *N*_*E*_), as evidenced by the large peak at 0 in the histogram for the E cell spike counts in Fig 3B, left panel.

To confirm that dense firing of the active E cells is a generic problem of model 1 and not due to our choice of $\bar{g}$ and *n*_*E*_, we systematically varied these parameters. The results are shown in Fig 4. We find that robust HFOs in the ripple range accompanied by sparse firing of E cells do not occur in model 1: The rainbow coloring in Fig 4A, upper subpanels, marks the region where the expected PC and PV+BC population activity oscillates in the ripple frequency range (upper subpanels in A). The magenta part of the lower subpanels indicates the region where the standard deviation of the oscillation frequencies across different network realizations is small (lower subpanels in A). Networks with parameters that fall into both regions can be expected to generate robust ripple frequency oscillations. However, in the entire region where the expected oscillation frequency is in the ripple frequency range, an active pyramidal cell spikes on average more than 5 times during the whole SPW/R event (Figs 4B and 3B). This is in marked disagreement with experimental findings [17], which show that most pyramidal cells fire once or twice during a SPW/R. More recent experiments even observed that E cells typically spike only once per SPW/R [19]. The white horizontal line in Fig 4A roughly delimits the region where E cells spike sparsely. (Notably also the I neurons usually spike sparsely above the line.) Anywhere below this white line, i.e. for higher values of $\bar{g}$, *C*_*E*_ is larger than two, which is biologically implausible.

Thus, in our model 1 with temporally broad excitation of E cells, in the absence of external feedforward excitatory drive to inhibitory cells and with unstructured, random connectivity, it is not possible to reach the ripple frequency range with sparse firing of pyramidal cells by changing $\bar{g}$ and *n*_*E*_. This remains true if *p*_*IE*_ is increased from 0.1 to 0.2 (S2 Fig). We increased *p*_*IE*_ because one might expect that more inhibitory inputs received by each PC result in fewer E spikes; however, there still is no region where HFOs in the ripple range co-occur with small values for *C*_*E*_. Increasing *p*_*IE*_ further and also increasing the width of the sharp wave *σ*_*g*_ to distribute the firing of the PCs does not result in lower firing of the E cells but instead lowers the frequency of the network oscillations to a maximum of approximately 170 Hz (S3 Fig). We note that larger network frequencies can always be obtained by increasing *p*_*II*_ and decreasing *p*_*IE*_ (S4 Fig); however, there still is no region where HFOs in the ripple range co-occur with small values for *C*_*E*_.

Finally, we include a strong feedforward excitatory drive to the I cell population. Such strong drive to the I cells renders our model an interneuron ripple model (see Introduction). This is because the I cell population already generates the observed oscillation, while the impact of the E cells is small. This is shown in S5 Fig, where each PV+BC is driven with a conductance as in Eq 6 with *σ*_*g*_ = 10 ms. All I cells get the same amplitude $\bar{g}=20\;\text{nS}$, which results in robust oscillations at *f*_*I*_ ≈ 165 Hz in the absence of the pyramidal cell population. With this modification, we now observe a region in parameter space where, for small *n*_*E*_ and $\bar{g}$, robust HFOs in the ripple range with small *C*_*E*_ < 2 are reached. Analogous to Fig 3, we show representative network activity in S6 Fig. Due to the weak drive and strong inhibition, E cell spiking is very sparse: in S6 Fig, less than 30 E cells are active, which is less than 1% of the whole population. Therefore, E activity does not discernibly influence the rhythmic I spiking. Although E cell activity is sparse, some E cells still spike more than twice during the ripple (S6 Fig).

Thus, in the presence of strong feedforward excitation to the inhibitory cells, model 1 leads to both network and single cell statistics consistent with the experimental data. However, the experiments of [19], find that strong drive to the I cells is not necessary to optogenetically evoke SPW/Rs, that it is insufficient to generate LFP HFOs in the ripple range *in vivo* and in fact, abolishes ongoing ripples (Fig 5E in [19]). Moreover, we find in our models that with the inclusion of a strong feedforward drive to the I cells, I cells start to spike before E cells in each ripple (cf. S6 Fig), which is not observed in experiments, where most E cells spike before I cells on all ripple cycles [17, 19]. We have not been able to find parameters alleviating the latter problem, but cannot exclude their existence. Given that strong excitatory feedforward drive to the I cell population seems to be a prominent feature of CA1 [88, 89], we consider it as an in principle biologically plausible option to decrease the activity of E cells in our model 1.

In conclusion, model 1 with broad temporal excitation of E cells and random connectivity does not appear suitable to describe SPW/Rs, where high network frequencies in the range from 140 to 200 Hz should co-occur with sparse firing of PCs.

### Temporally narrow excitation of pyramidal cells (model 2)

We next ask whether the biologically implausible frequent E cell firing during ripple frequency oscillations in our two-population models can be avoided by providing excitation to CA1 pyramidal cells that is shorter, pulse-like, and received at different times. For this we distribute the input peak times across the pyramidal neurons according to a Gaussian distribution, $t_{0}∼N\left(50\;\text{ms},\sigma_{t}\right)$. The individual excitation pulses have each a width of *σ*_*g*_. For simplicity, we assume that all pulses have the same amplitude, $g_{\text{ext}}^{0}=\bar{g}$, such that all PCs receive the same amount of excitation, but at different points in time.

**Fig 5:**
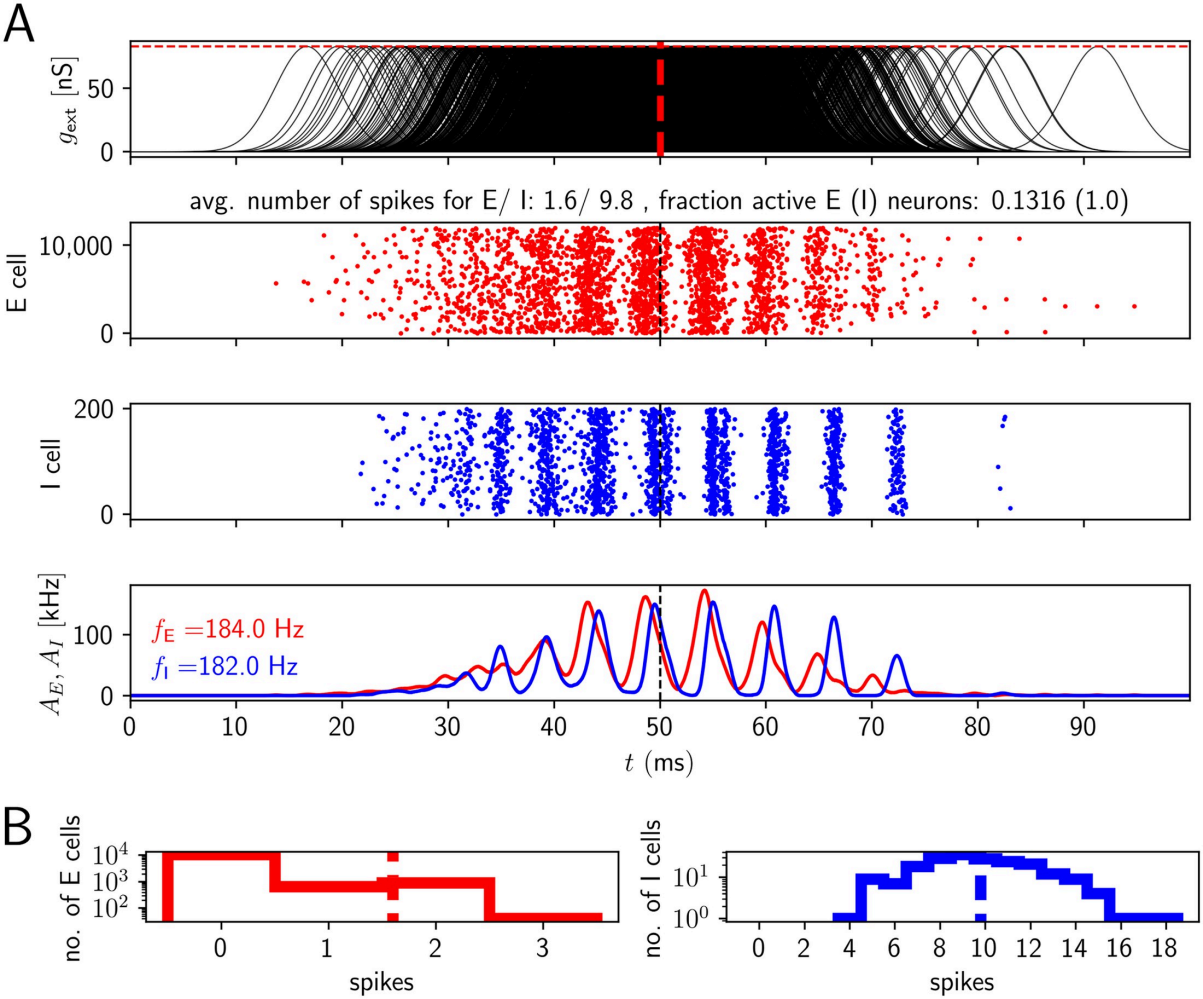
Network activity for temporally narrow excitation of E cells (model 2). Each E cell receives an input pulse whose duration is comparable to the interval between two ripple waves. The peak times of the pulses are distributed. E and I populations spike at ripple frequency, active E cells typically contribute one to two spikes to an event. A: Input pulses, spike rastergrams and network activities, displayed as in Fig 3. B: Histograms of spike counts during the displayed event. Parameter values as in Fig 6, $\left(\bar{g},n_{E}\right)=\left(83\;\text{nS},1580\right)$.

A sample output of this model for *σ*_*g*_ = 3 ms is shown in Fig 5. Robust oscillations in the ripple range are reached in the region around $\left(\bar{g},n_{E}\right)=\left(83\;\text{nS},1580\right)$. In contrast to model 1 (see Fig 4), active E cells typically contribute 1 or 2 spikes to an event.

**Fig 6:**
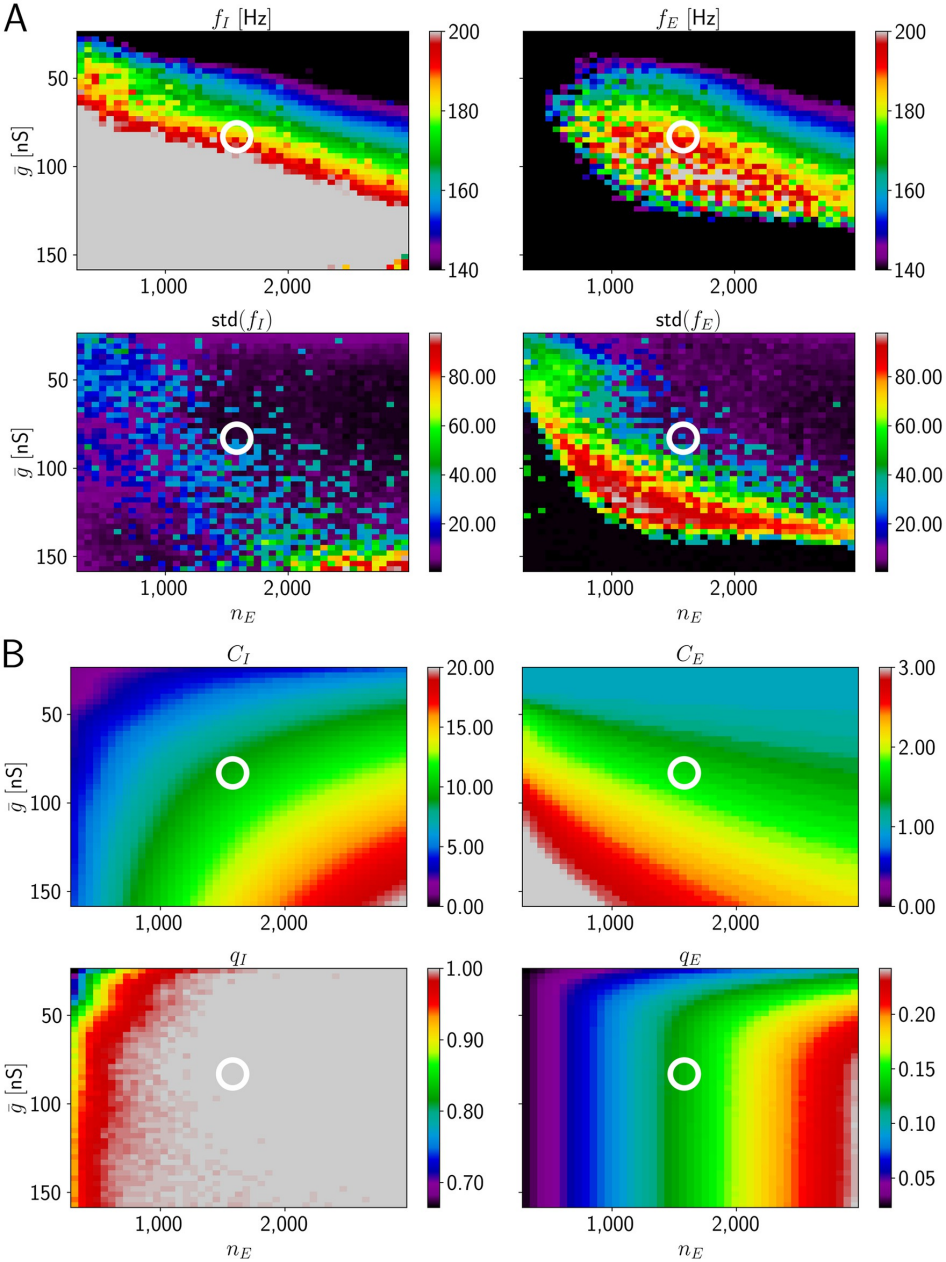
HFOs in networks with temporally narrow excitation of E cells (model 2). Ripple oscillation generation with sparse spiking of E neurons is robust in networks where the E cells are driven by short input pulses. Figure layout as in Fig 4. A: Frequency of population activity oscillations and standard deviations. B: Number of spikes per active neuron and fraction of active neurons. The ranges for *C*_*I*_ and *C*_*E*_ are [0, 20] and [0, 3], respectively. The white circle is located at $\left(\bar{g},n_{E}\right)=\left(83\;\text{nS},1580\right)$. Parameters: *σ*_*t*_ = 10 ms, *σ*_*g*_ = 3 ms.

There is a broad region in the parameter space spanned by $\left(\bar{g},n_{E}\right)$ where HFOs in the ripple range co-occur with sparse firing of E cells (Fig 6). This region lies at intermediate values of $\bar{g}$. An increase (decrease) in $\bar{g}$ has to be accompanied by an increase (decrease) in *n*_*E*_ to stay in this region (Fig 6). Thus, higher levels of excitation have to be distributed over more E cells to stay in the ripple range.

To maintain sparse firing of E cells, individual external input pulses need to be narrow. In S7 Fig, we increase their width to *σ*_*g*_ = 5 ms, which results in more E cell spikes per active E cell (between 2 and 3 spikes on average in the region where ripple frequency oscillations are generated).

As shown before for model 1, higher oscillation frequencies can be reached by increasing *p*_*II*_. Additionally increasing *p*_*IE*_ renders the oscillations more robust and the region in parameter space where HFOs in the ripple range are generated increases (S8 Fig). We show in S9 Fig that the network dynamics and statistics stay realistic for longer sharp waves. For this we increase *σ*_*t*_, which controls how broadly the pulse peaks are distributed over time (Eq 6), in addition to increasing *p*_*II*_ and *p*_*IE*_. In S10 Fig we show the network dynamics and statistics when we decrease *p*_*II*_ back to 0.2 (keeping *p*_*IE*_ = 0.2) compared to S9 Fig. Again, there is a regime where high network frequencies and sparse firing of pyramidal neurons coexist.

In conclusion, for model 2 the generation of HFOs in the ripple range with sparse firing of pyramidal cells does not depend much on the details of how the recurrent I-to-E and E-to-I loop are set up. Instead, the main determinant for the sparseness of pyramidal cell activity are the properties of the afferent, pulse-like CA3 drive. In the next section, we show that dendritic spikes generated by apical and basal dendrites of CA1 pyramidal cells might give rise to appropriate temporally localized input.

### Dendritic spikes provide short windows of excitation for pyramidal cells (model 3)

The apical and basal dendrites of CA1 pyramidal cells can, when excited with sufficiently synchronous and spatially clustered inputs, generate dendritic spikes that evoke pulse-like depolarizations in the soma [67–70, 73]. The Schaffer collaterals, which transmit the sharp wave from CA3 to CA1, target these apical and basal dendrites [1, 90]. It has been shown for apical dendrites of CA1 neurons that dendritic spikes occur during sharp-wave activity *in vivo* [84], as well as pulse-like depolarizations and action potentials in the somata. These observations indicate that during SPW/Rs, the CA3 spikes forming the sharp wave input to CA1 generate dendritic spikes in CA1 neurons, which leads to pulse-like somatic depolarizations. Ref. [69] suggested that dendritic spikes observed in the apical dendrites of CA1 pyramidal cells endow the cell with unique information processing capabilities during SPW/Rs. Specifically it was shown that these dendritic spikes result in precise somatic spikes with low temporal jitter. This led to the conjecture that dendritic compartments receiving input clustered both in space and time perform supralinear dendritic integration during SPW/R events. The dendrites might then act as feature detectors on the CA3 input and determine the neuronal action potential output. This would be largely independent of the mean input strength in contrast to the output during theta oscillations [69].

Consistent with these findings and suggestions, we propose that dendritic spikes may provide the short-term excitation that we found to be necessary for HFOs with sparse E cell firing in model 2 (see Fig 6 and S7 Fig). For this, we incorporate in our CA1 E neurons dendritic spike generation in response to input from CA3. Since dendritic spikes are generated by coincident spike arrivals, we introduce a simple model of CA3 with *N*_*E*,CA3_ pyramidal cells, each of which fires according to an inhomogeneous Poisson process. This has a peaked rate profile with the same amplitude and width across all CA3 neurons (see Eq 7, the peak is at *t*_0_ = 50 ms, width is *σ*_*t*_ = 10 ms). When there are at least five input spikes arriving within a time window of 2 ms, a dendritic spike is elicited. Dendritic spikes impact a neuron’s somatic voltage in a stereotypical fashion. Between neurons, their impact varies: the peak current elicited by a dendritic spike in the soma is distributed according to a lognormal distribution (Eq 2) across the CA1 E cells. Each CA1 PC receives inputs from a small fraction of all CA3 pyramidal cells. We set the connection probability to $p=\frac{130}{N_{E,\text{CA3}}}$, so that the average number of CA3 input connections to a CA1 E cell is 130. The size of the modeled CA3 population is *N*_*E*,CA3_ = 15000 and their peak rate is *r*_0_ = 8 Hz [65, 91, 92] (see Materials and methods for further details). Besides their contribution to dendritic spikes, the inputs from CA3 elicit EPSPs with a peak of 0.4 mV when the neuron is otherwise held at −55 mV.

**Fig 7:**
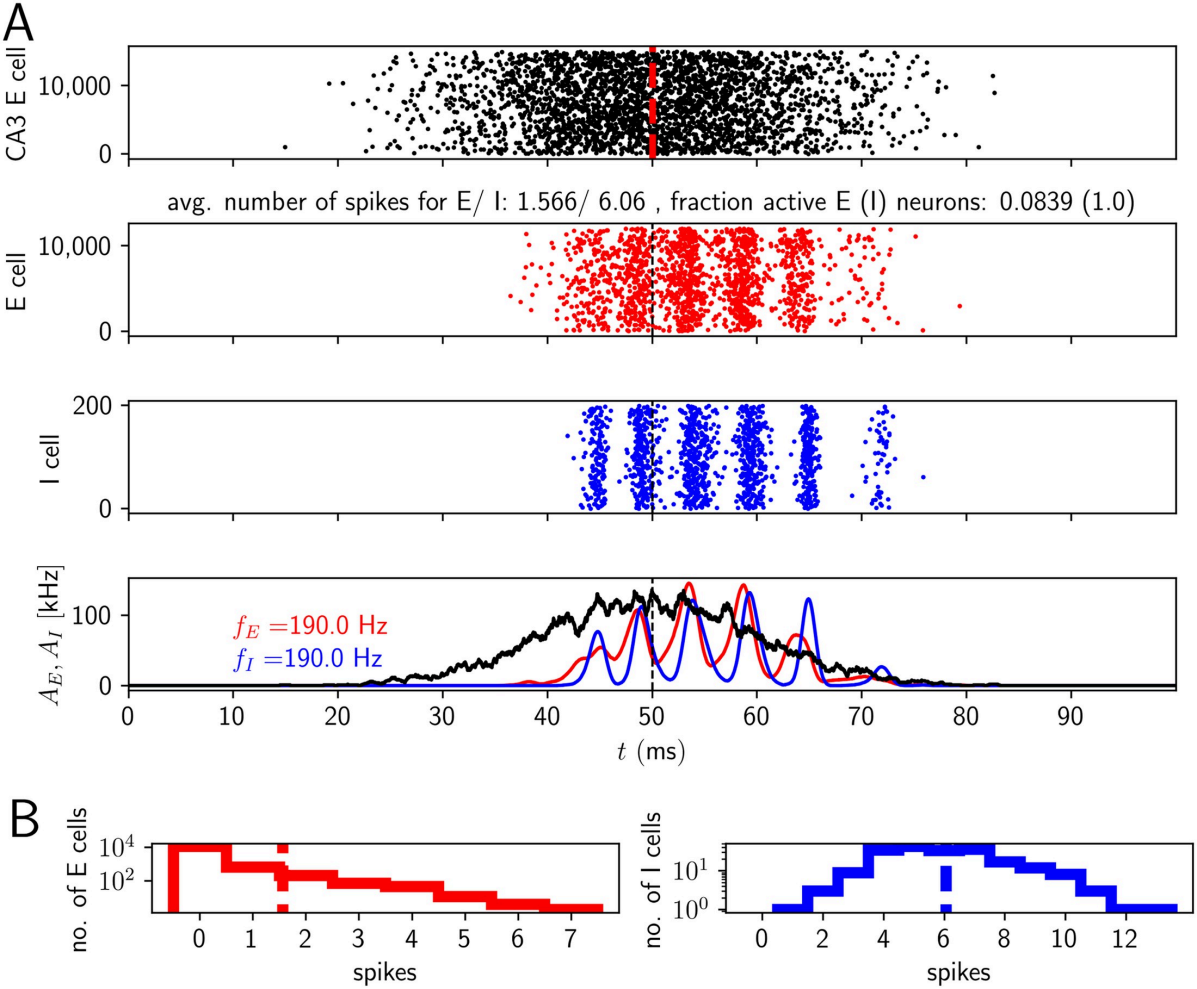
Activity in a network with nonlinear dendritic excitation of E cells (model 3). E and I populations spike at ripple frequency, active E cells typically contribute one or two spikes to an event. A, upper subpanel: Spikes generated by CA3. Middle: spike rastergrams of the CA1 E and I cells (red and blue). Lower: population rates (red: population of E cells, blue: population of I cells, black: population of CA3 cells). B: Histograms of spike counts. Parameter values as in Fig 8, (*σ*, *μ*) = (0.75, 0.0). Fraction of active E/I cells (*q*_*E*_, *q*_*I*_): 0.084, 1. Number of spikes per spiking E/I cell (*C*_*E*_, *C*_*I*_): 1.6, 6.1.

Fig 7 shows a representative simulation of a model 3 network generating HFOs in the ripple range accompanied by sparse firing of pyramidal cells. The mean number of spikes in the active E cell population is between 1 and 2 (approximately 1.6 in Fig 7) and most active E cells spike once or twice. A small fraction of neurons spikes 5 times or more during the SPW/R event (left histogram in Fig 7B). Such ‘bursting’ behavior is caused by large values for the peak of the dendritic current and/or exceptionally high synchrony in the presynaptic spike trains impinging on a CA1 PC. We will see below that it is not crucial for generating SPW/Rs in our models (S15–S18 Figs). Bursting of pyramidal cells in CA1 was observed *in vivo* during slow wave sleep [58, 93], in particular during SPW/Rs [36, 94].

Why does model 3 generate sparse E cell firing in contrast to model 1, if in both models the CA1 E cells receive a broad sharp wave input? In model 3, CA1 receives noisy Poisson spiking and each E neuron has a short integration window for dendritic spike generation. Such a short window generates little averaging over the input spikes. Therefore the fluctuations in the number of spikes arriving within the window are large compared to the average. When the temporal density of input spikes to a CA 1 E neuron is by chance exceptionally high, a dendritic spike is generated. This happens rarely, but not with negligible frequency, see also S3 Appendix for a numerical analysis and analytical computations making this more precise. The resulting sparse dendritic spiking provides narrow, pulsatile, and often strong inputs to these E cells, which leads like in model 2 to their sparse spiking.

We note that compared to model 2, we have decreased the synaptic latency from I to E cells from 1.0 ms to 0.5 ms. This stabilizes the oscillations, but is not essential, see S14 Fig. That it is helpful can be intuitively explained as follows: The spike rate of the CA3 population (given by Eq 7) is towards its peak sufficiently high to generate widespread spiking in the CA1 pyramidal cells. Fast recurrent inhibition within CA1 is required to terminate a single pyramidal ripple wave; in the absence of inhibition it would go on as long as CA3 spiking is strong enough. Inhibition has to impinge early enough on the excitatory population on every ripple wave to prevent an excess of excitation and thus a slowing of the oscillation due to resulting excessive inhibitory spiking. Therefore, a parameter change that lets recurrent inhibition arrive earlier decreases the number of E spikes and renders the oscillations more pronounced and robust. Any mechanism that decreases the width of the excitatory ripple wave will have a similar effect. In addition to the PV+BCs considered here, such silencing of pyramidal cells could be provided by bistratified cells [95, 96] or feedforward inhibition [97].

**Fig 8:**
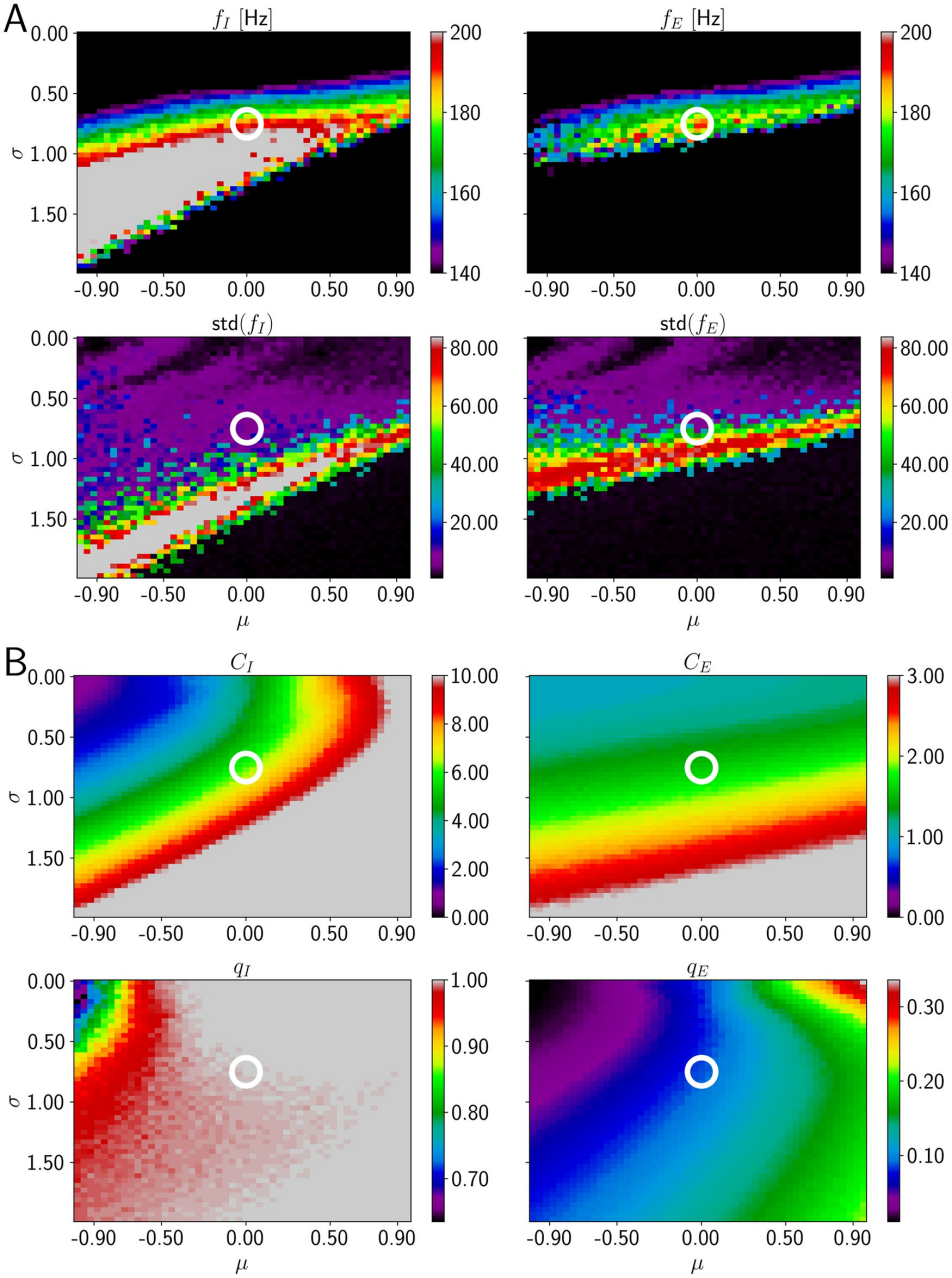
HFOs in networks incorporating dendritic excitation (model 3). In the parameter space of the distribution for the peak dendritic current, a region with reliable ripple oscillation generation and sparse spiking of E neurons exists in model 3. The layout of the figure is similar to Fig 4; the changed variables are now the parameters *μ* and *σ* of the lognormal distribution of dendritic spike impacts on the soma (Eq 2). A: Network frequencies and standard deviations as a function of *μ* and *σ* (Eq 2). The range displayed in detail by different colors is the ripple range, [140, 200] Hz. Frequencies above and below are shown in gray and black. B: Number of spikes per active neuron and fraction of active neurons. The displayed ranges for *C*_*I*_ and *C*_*E*_ are [0, 10] and [0, 3], respectively, values above are colored in gray. The white circle is located at (*σ*, *μ*) = (0.75, 0.0), the parameter values of Fig 7.

We now systematically vary the parameters *σ* and *μ* of the lognormal distribution for the dendritic spike impact strength (Eq 2) in Fig 8 and find that biologically plausible ripple activity occurs robustly: For intermediate values of both *σ* and *μ* (rainbow colored band left and right of white circle in Fig 8A upper panels), the system generates fast oscillations in the ripple range with realistic single-neuron firing statistics. To stay in the ripple range, an increase in *μ* can be compensated by a decrease in *σ*. For a fixed value of *μ*, an increase of *σ* beyond a certain value renders the oscillations non-robust: the oscillation frequency varies considerably across different network realization (Fig 8A lower panels).

E cell spiking is sparse all over the range where ripple frequency oscillations are generated: *C*_*E*_ is approximately constant, between 1 and 2 (Fig 8B). The amount of I cell firing depends on the precise values for the parameters of the peak dendritic current. For negative values of *μ* and *σ* ≈ 1, I cells fire on average three times per ripple. Moving along the diagonal where ripple oscillations are generated to values of *σ* ≈ 0.5 and *μ* ≈ 0.9, increases *C*_*I*_ to more than 10 spikes per ripple. The range in between appears consistent with the experimental observation that CA1 PV+BCs typically spike on every ripple wave.

We now discuss the impact of certain selected parameter changes on model 3 to obtain an understanding of the robustness of our findings. Simulations for higher I-to-E connection probability (*p*_*IE*_ = 0.3 instead of *p*_*IE*_ = 0.1) are shown in S11 Fig. The band where HFOs in the ripple range occur has moved to slightly larger values of *σ* compared to Fig 8. E cell firing is sparse within the band. Thus, model 3 is robust to a higher I-to-E connection probability. We argue in the Materials and methods that *p*_*IE*_ should be between 0.08 and 0.17 for the network sizes we consider in this paper. Given that HFOs in the ripple range are still observed with a higher value *p*_*IE*_ = 0.3, we are confident that the results of model 3 do not hinge critically on the precise value of the I-to-E connection probability.

We next increase the I-to-E synaptic latency *τ*_*l*_ from 0.5 ms to 0.9 ms (S12 Fig). The maximal oscillation frequency reached by the E population is reduced to approximately 150 Hz and the region where this occurs is shifted to smaller values of *σ*. E cell firing remains sparse. However, we also observe that larger *τ*_*l*_ can be compensated by a decrease in the AMPA rise time on I cells ($\tau_{\text{exc},r}^{I}=0.1\;\text{ms}$ instead of 0.5 ms, such that the resulting model generates HFOs in the ripple range with sparse firing of E cells, see S14 Fig). We note that additionally the peak E-to-I synaptic conductance was decreased (as in S13 Fig). The results indicate that in model 3 fast synaptic transmission from the I to the E population is important, but a slower transmission can be compensated by changes in other parameters.

We also decreased the peak E-to-I conductance $g_{\text{exc},\text{peak}}^{I}$ from 3 to 1 nS still in the experimentally observed range [81, 82]. This results in more robust oscillations in the ripple range (S13 Fig). Further, the region in the (*σ*, *μ*) parameter space where HFOs exist is enlarged compared to Fig 8, it now has the shape of a diagonal stripe extending from negative values for *μ* and large values for *σ* to positive values for *μ* and small values for *σ*. This appears broadly consistent with in *in vivo* experiments in transgenic mice, which found increased ripple power when reducing AMPA receptor-mediated excitation on PV+BCs [98].

Finally, we study whether the large values at the tail of the lognormal distribution of dendritic spike impacts are important. We thus truncate the distribution given by Eq 2 such that values of $I_{\text{dendritic}}^{\text{peak}}$ larger than 4 nA are mapped to 0. With this truncation, a single dendritic spike can generate at most (and then barely) two somatic ones (see Fig 2C for an example showing two somatic spikes as a response to one dendritic spike with a peak amplitude of 5 nA). Thus, with this truncation, there can be no somatic bursting due to a single dendritic spike. We find that this reduces the maximal frequency that can be reached (S15 Fig). However, frequencies around 170 Hz still occur. The standard deviation across network realizations is low in the corresponding regions, albeit higher than in Fig 8. Additionally, we checked that the low ripple range (∼ 150 − 160 Hz) can still be reached when the truncation is introduced at 3 nA, but not when it is introduced at 2 nA.

We additionally study a modified way to truncate the distribution for the peak dendritic current (S16 Fig). Instead of setting values above the cutoff to zero, we re-sample values from the lognormal distribution until each value for the peak dendritic current is smaller than 4 nA. This leads to results similar to those in S15 Fig, but with higher frequencies in the ripple range around 190 Hz (see red region next to white circle in S16A Fig, upper panels). The fact that ‘outliers’, which are eliminated by the truncation, are not crucial for the generation of HFOs in model 3 also suggests that our choice for the distribution of the peak dendritic currents (Eq 2) is not unique. Indeed, we find that results similar to Fig 8 can be obtained with a Gaussian distribution for the peak dendritic current that has the same mean and standard deviation as the corresponding lognormal distribution (S17 Fig). We also find that increasing the absolute refractory period of each CA1 PC to the unrealistically high value of 200 ms still results in networks generating robust HFOs in the ripple range (S18 Fig). With this absolute refractory period, the firing of the E cells is certainly sparse as they can at most spike once during a simulation of duration 100 ms. This shows that while there can be bursts of CA1 PC activity in our model, they are not necessary for the generation of HFOs in the ripple range.

In conclusion, high values for the peak dendritic current leading to somatic bursts (multiple PC spikes during one ripple wave) aid the model in generating fast and robust HFOs. They are, however, not necessary: after truncating the lognormal dendritic spike strength distribution, replacing it with a Gaussian one, or preventing E neurons altogether from generating multiple spikes by a long somatic refractory period, robust HFOs in the ripple range are still generated.

### Intrinsic CA1 replay and ripple oscillations by pulse and gap coding

A prominent feature of SPWRs in CA1 is that they occur in conjunction with replay of activity sequences from previous periods of exploration and learning [2, 94, 99–101]. Recent experimental results show that CA1 can generate sequence replay intrinsically without structured input from CA3 [36]. Further experimental evidence indicates that place cells in CA1 and CA3 have different properties [93, 102]. One possible explanation for the intrinsic replay in CA1 is that it is based on recurrent excitatory connections that are amplified due to dendritic spikes in the basal dendrites of CA1 [41, 42]. In this model, pulses of excitatory spikes travel along pathways in the sparse recurrent connectivity, which is enabled by amplification by dendritic spikes in the basal dendrites. Replay of spike sequences might also arise from continuous attractor dynamics. Here a localized bump of activity in the neural tissue moves around because of asymmetric synaptic connections, short-term plasticity, or adaptation mechanisms [103–107]. Sequential network structures might guide these bumps to replay certain sequences. Due to the very sparse excitatory recurrent connectivity, also propagation of inhibitory spikes pulses in an essentially inhibitory network as suggested for the striatum [108], might be considered biologically plausible for CA1. However, given the high firing rate of I neurons during ripples (as mentioned before, PV+BCs fire on nearly every ripple wave [1, 17]), it seems unlikely that their population activity forms sequential patterns similar to that of PCs. In support of this, it is also known that I cells have broader, more unspecific place fields than E cells [83, 97] (see however [109] and [110]).

**Fig 9:**
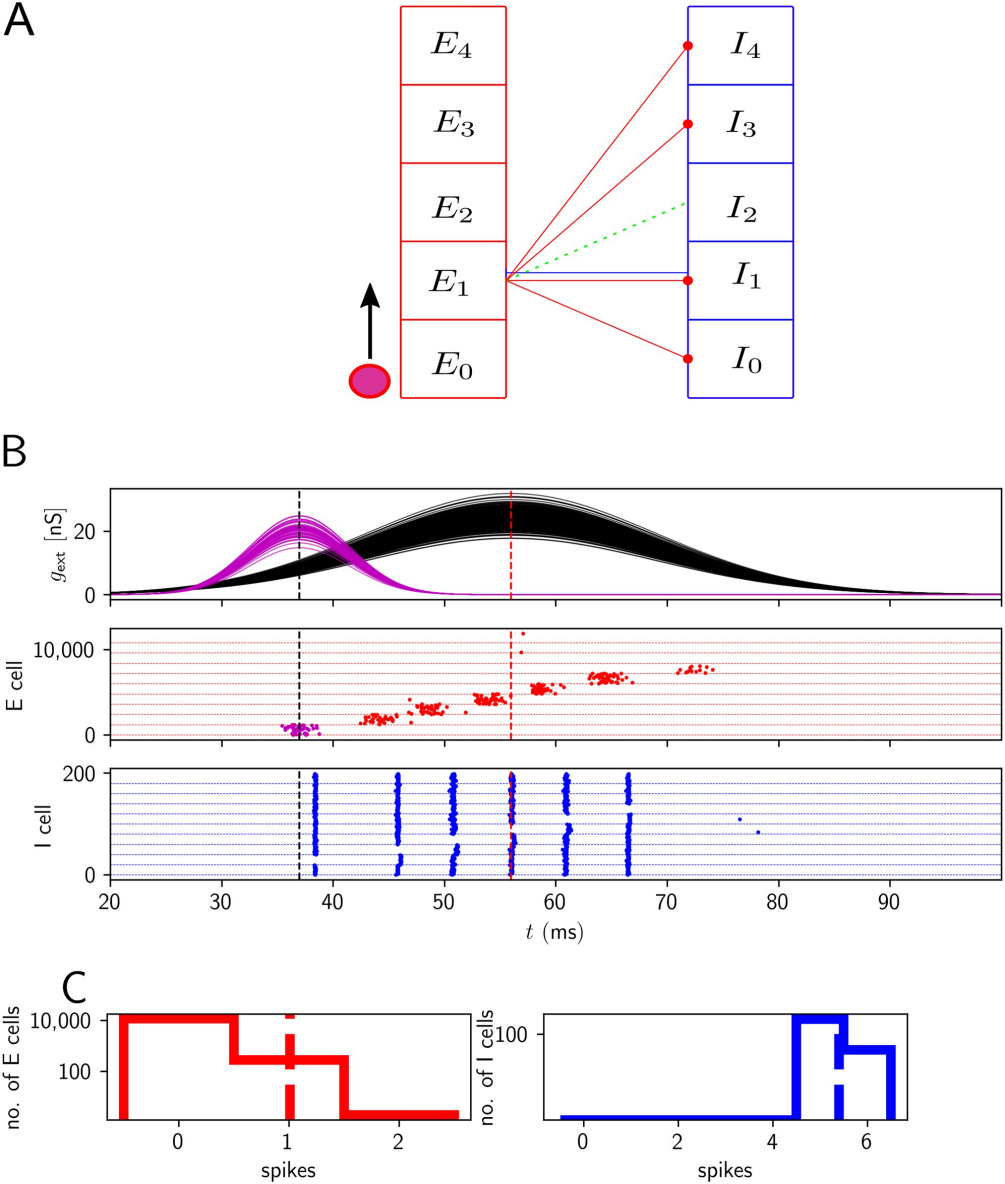
Replay events and ripple oscillations induced by pulse and gap coding of E cells and I cells. The sequence replay is based on disinhibition of a group of E cells and the resulting excitation of all but one group of I cells, which in turn disinhibits another group of E cells. A: Connectivity scheme. Both the E cells and the I cells are grouped into *K* + 1 groups (here: *K* = 4 was chosen for simplicity, in the simulations below, *K* = 9). Group *E*_1_ excites all groups of I cells except I_2_ (green dashed line). Similarly, group *E_k_* excites all groups *I_l_* except Ik+1(l=0,…k+1,…K). Group *I_k_* projects on a single group *E_k_*; that is, group *I*_0_ inhibits group *E*_0_, group *I*_1_ inhibits group *E*_1_, and so on. All connections relevant for the sequence generation are shown for group *E*_1_ and *I*_1_. A replay event is triggered by the initial stimulation of group *E*_0_. The subsequent activation by CA3 lets E neurons spike when they are not inhibited. The activity progresses in the direction of the black arrow: *E*_0_ activates all I neurons except those forming group *I*_1_. Since *I*_1_ does then not inhibit *E*_1_, these become active due to the random, unstructured CA3 input. This excites all I cells except those forming group *I*_2_. The resulting gap in inhibitory activity generates a pulse in E cell group *E*_2_ on the next ripple wave etc. B: Example realization with model 1, i.e. with temporally broad excitation of E cells similar to Fig 3. The network generates replay activity together with an oscillation of frequency *f* ≈ 190 Hz. Neurons of group *E*_0_ are activated by an initial part of the sharp wave. We have implemented this by a short input pulse (upper panel, magenta) preceding the main sharp wave. The E neurons in the other groups receive temporally broad inputs from the main sharp wave (black). About seven E and I groups are sequentially (de)activated, before the sequence terminates due to the termination of CA3 input. C: Histograms of spike counts during the displayed event. Parameters are as in Fig 4, except for those listed in S2 Appendix.

Motivated by the high sparseness of excitatory-excitatory connectivity in CA1 and the supposed absence of replay in inhibitory activity, we propose an alternative model for how specific sequences may be stored and replayed (schematically depicted in Fig 9A). Like the proposed ripple generation mechanism for random networks, sequence generation and the thereby evoked oscillations are based on the prominent excitatory-inhibitory and inhibitory-excitatory connectivity in the hippocampal region CA1. The basic idea is as follows: CA3 excites an initial, zeroth group of CA1 E cells to spike within a short time interval [111]. This group excites all CA1 I cells involved in the dynamical pattern, except for the first group of I cells. This group would inhibit the first group of E cells. The gap in its activity (I cell ‘gap coding’) disinhibits the first group of E cells, such that it becomes active in pulse-like manner (E cell ‘pulse coding’). It excites all I cells except for the second group that would inhibit the second group of E cells. Therefore the second group of E cells becomes active and so on (Fig 9A). A generalization to continuous, non-grouped activity sequences [28, 42] seems straightforward. Further alternative concepts for replay, which we do not consider as they seem less biologically plausible for CA1, are presented in S19 Fig. The first such concept (S19A Fig) is a two-population model assuming that both the E and I populations use pulse coding, such that both E and I cells spike in a sequential fashion: the active group of E cells excites the next group of I cells, which inhibits all E cells except those that fire in the next step. The second concept is an inhibition-first model, which generates sequences by pure gap coding in the I population (S19B Fig): groups of I cells become sequentially inactive, since they receive at some point more inhibition than their peers because of structured inhibitory connectivity. The resulting disinhibition of E cells leads to their sequential activity, but their spiking does not contribute to the maintenance of the sequence.

We first show that the E pulse and I gap coding scheme generates sequences and biologically plausible ripples in model 1, i.e. with temporally broad excitation of E cells: Fig 9B displays a replay event in a model 1 network structured according to Fig 9A. Active E cells typically spike once and only rarely twice. Thus, the high spike frequency of active E cells in model 1 is avoided. All I cells are active and spike on nearly every ripple wave. The used parameters are similar to those in Fig 4 (see S2 Appendix for details). The introduced parameter changes decrease the sharp wave input strength and the strength of E-to-I synapses. The former ensures that I cell spiking can suppress E cell spiking effectively, the latter prohibits overly high I cell activity.

**Fig 10:**
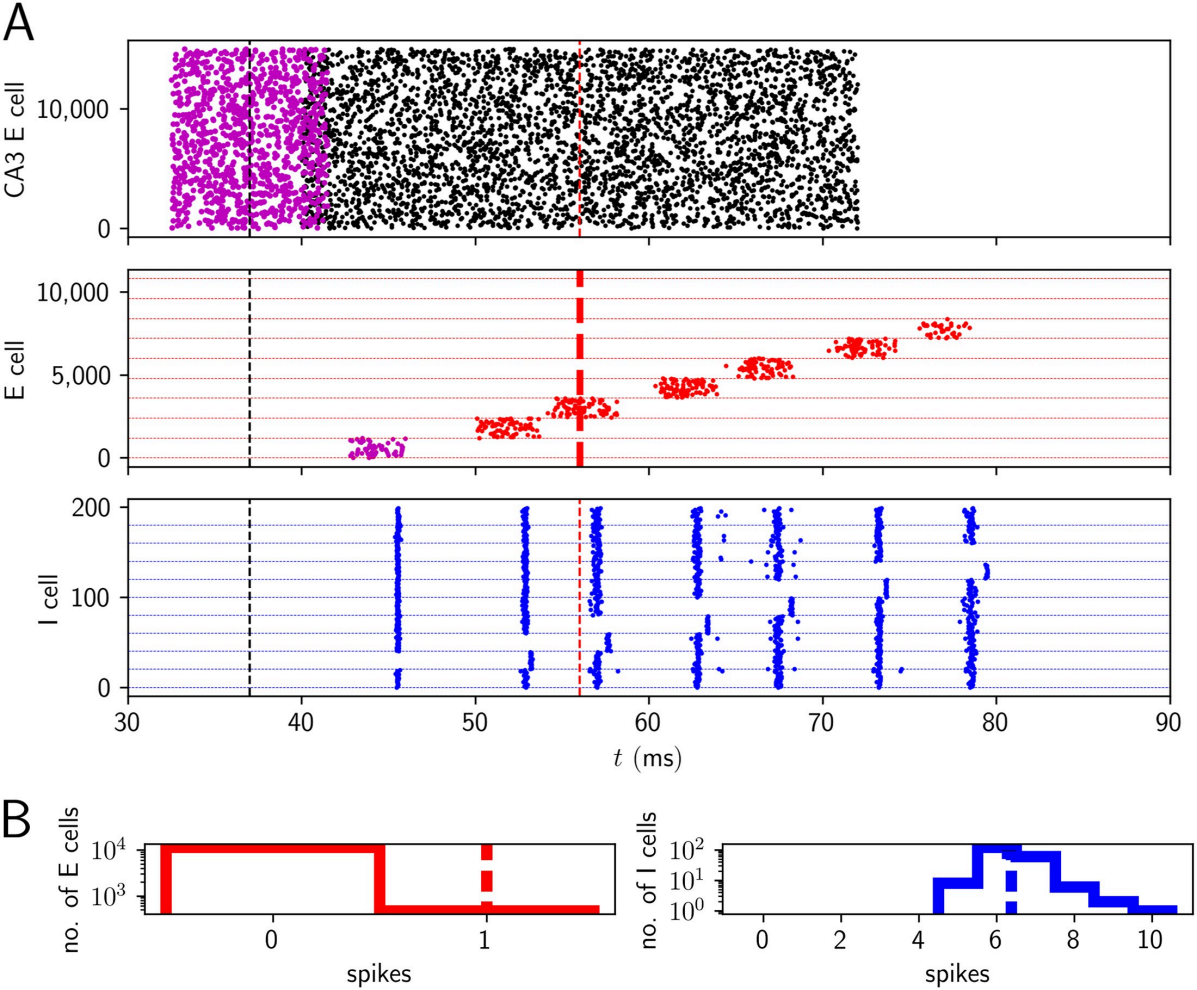
Replay events and ripple oscillations in model 3. Appropriately structured model 3 networks with relatively weak dendritic spikes generate replay and oscillations in the ripple frequency range through alternating E pulse and I gap coding. Active E cells contribute only one spike, while all I cells participate and spike in nearly every ripple. The populations are split into *K* + 1 = 10 groups forming a chain as in Fig 9A. The figure layout is similar to Fig 7. A, upper subpanel: Spikes generated by CA3. Middle: spike rastergrams of the CA1 E and I cells (red and blue). Example realization in a network with *N*_*E*_ = 12000 excitatory cells and *N*_*I*_ = 200 inhibitory cells generating replay together with an oscillation of frequency *f* ≈ 190 Hz. Group *E*_0_ receives input from an initializing CA3 population here consisting of 15000 neurons (magenta spikes, population size chosen like that of the main sharp wave to keep the connection probability constant across neurons). The remaining groups of excitatory cells receive unstructured input from *N*_*E*,CA3_ = 15000 different excitatory CA3 spike trains shown in black (for simplicity the same rastergram as for the magenta spikes was used). About seven E and I groups are sequentially (de)activated, before the sequence terminates due to the termination of CA3 input. B: Histograms of spike counts. Parameters are as in Fig 8, except for those listed in S2 Appendix.

Fig 10 shows sequence and ripple generation by E pulse and I gap coding in a model 3 network structured according to Fig 9A. There are dendritic spikes amplifying the input from CA3 to CA1, but their impact is weakened compared to Fig 8. This, together with further parameter changes (see S2 Appendix for details) ensures that CA1 E cells only spike if they ‘see’ the inhibitory gap, because otherwise inhibition is strong enough to suppress them. Every active E cell in Fig 10 spikes exactly once. All I cells are active and spike on nearly every ripple wave.

## Discussion

In the current article, we have studied two-population models for the generation of ripples and sequences in the hippocampal region CA1. In these models E and I neurons interact to generate the ripple rhythm possibly together with sequential activity. This is motivated by recent *in vivo* experiments, which have shown that both the local PC and the local PV+BC populations in CA1 contribute to generating ripple oscillations [19]. Further motivation comes from the observation that the connectivity from local PCs to PV+BCs and back is high (Materials and methods). Our models are constrained by biologically plausible connectivity and by the fact that during ripple oscillations, basically all PV+BCs spike at high frequency, i.e. on nearly every ripple wave [17, 36, 60–62], while the PCs spike sparsely, contributing typically only one or two spikes [17, 19]. The latter implies that the spiking activity during CA1 SPW/Rs is a mixture of two often considered oscillation types: strongly synchronized [33] and weakly synchronized oscillations [21, 23, 34]. The E cell population is weakly synchronized; the average single-cell firing frequency during a ripple is much lower than the population frequency. The I cell population (consisting of PV+BCs) is strongly synchronized, with every I cell spiking at nearly each individual ripple wave.

We observe that temporally broad CA3 sharp wave input generates in random CA1 two-population model networks ripple frequency oscillations or sparse spiking of E neurons, but not both, unless there is strong feedforward inhibition. Sparse spiking of E neurons can be reached if there is a strong CA3 projection to the I neurons, such that the oscillations are effectively generated by the I population alone. We were, however, unable to achieve with the model the experimentally found phase relation of E and I neuron spiking [17, 57], which is equivalent to a 1.2 − 2.0 ms lag of the I neuron spike peak behind the E neuron peak. Further, experiments indicate that ripples are generated by the interaction of local E and I neurons without requiring feedforward input to the I neurons [19] (see, however, [26] for a different explanation of the results). In non-random, sequence generating networks, we observe biologically plausible ripples generated by the propagating activity. We suggest that appropriate sequential activity may be realized by pulse-coding of E and gap coding of I cells in CA1. This is motivated by the experimental observation that recurrent E-to-E connectivity in CA1 is very sparse [54, 55]. The sequential structure enables sparse E spiking. It has also previously been proposed that sequential activity may underlie ripple generation [28, 42]; the sequences there were generated due to enhanced E-to-E connections.

For random networks, we have explored the idea that the sparse spiking of E cells in CA1 originates from temporally sparse, short and strong inputs from CA3 (model 2) that lead to somatic spiking if inhibition is temporally weak. Different CA1 PCs receive these inputs at different times, their density is highest near the peak of the sharp wave. We propose in model 3 that such sparse inputs might originate from dendritic spikes in the apical dendrites, which are elicited when sufficiently many spikes arrive from CA3 within a short time window [67, 69].

**Fig 11:**
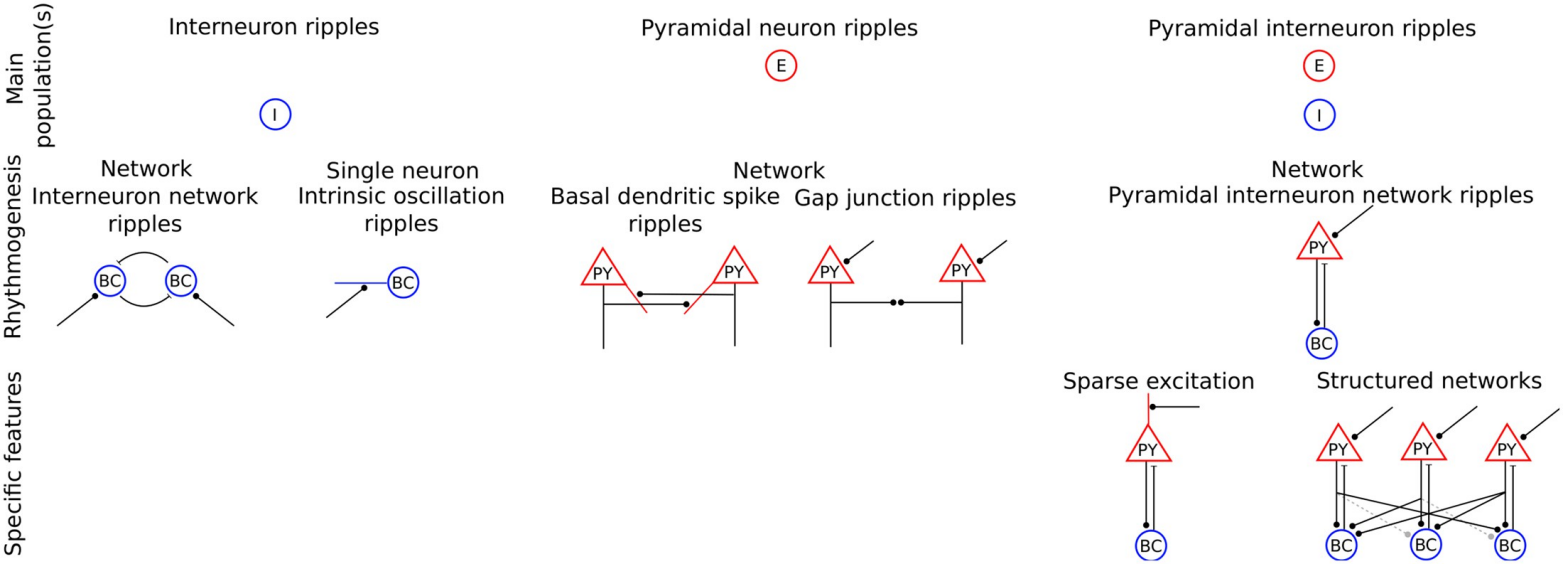
Our work in the context of previous CA1 ripple models. Visual depiction of our classification scheme of CA1 ripple models (see Introduction) with our models sorted in. The classification scheme has three levels, indicating (i) which neuron population or populations are mainly involved in the ripple generation, (ii) what the main mechanism for rhythmogenesis is (in particular if it is a network or single neuron mechanism) and (iii) specific features that are important for the overall rhythm. In the current article we have proposed new models (bottom right) of the class of pyramidal interneuron network ripples incorporating the specific features of sparse excitation (left) and structured networks (right, missing connections leading to sequential replay are gray dashed). These explain sparse pyramidal neuron and dense interneuron spiking during SPW/Rs. For clarity, specific model features of previous models are omitted, such as required structured networks for SPW/R generation in some basal dendritic spike models [42] and ripple generation by multiple disparate, inhibitorily coupled oscillators [36]. For our models one could have added further specific features such as recurrent inhibition. PY: pyramidal neurons, BC: PV+BCs.

A variety of models has been proposed to explain the mechanisms underlying SPW/Rs (see our classification in the Introduction and Fig 11). Given the current experimental knowledge, many of them are biologically plausible. Neurobiological ripples could also be generated by a combination of them: as an example, strong feedforward inhibition as in the variant of model 1 used in S6 Fig might support ripple generation in model 1 with structured networks (Fig 9) and in model 3. Furthermore, experimentally uncovered differences in some SPW/R properties might indicate that partially different mechanisms underlie *in vitro* and *in vivo* ripples (inhibition dependence [19, 112], action potential shape [44, 48]) as well as CA1 and CA3 ripples (optogenetic stimulation of PV+BCs [19, 25]), requiring different models. Our model aims at explaining *in vivo* experiments indicating that the local PC and the local PV+BC populations in CA1 contribute to generating ripple oscillations [19]. The designed models are therefore of the pyramidal interneuron ripple class. Related are previous interneuron network ripple models [21, 26, 28], other pyramidal interneuron network HFO models [21, 34, 38] and pyramidal neuron network ripple models [41, 42]. Compared to pure interneuron network ripple models, the inclusion of pyramidal cells and the condition of their sparse firing renders the generation of high frequency oscillations in interneuron network ripple models as well as in pyramidal interneuron network ripple models challenging. This is because the additional E-to-I and I-to-E loop usually slows down the network oscillation frequency [34]. We find that simply increasing the level of external excitation to increase the oscillation frequency often results in too much E cell spiking, which renders the models unsuitable to describe ripple oscillations. Previous models for HFOs incorporating two populations have assumed connection probabilities [23, 26, 34, 38] and/or spiking dynamics [21, 34, 38] that do not fit CA1 and/or its ripples [1, 17, 55]. Our current model 1 using structured connectivity and our model 3 with random and structured connectivity provide a pyramidal interneuron network ripple model incorporating biologically plausible connectivity and generating biologically plausible sparse E and dense I spiking [17, 36, 60–62] with realistic phase relations [17, 57]. Our model 1 with strong feedforward inhibition is an interneuron network ripple model, like [21, 26, 34]. In contrast to [26], which uses all-to-all connectivity, it has realistic, sparse connectivity. In contrast to the models in [21, 34], which generate sparse E and I spiking, it generates sparse E and dense I spiking. It has, however, an implausible phase relation of the peaks of E and I spiking (S5 and S6 Figs). It will be interesting to explore whether adding more biological detail to our model will allow to correct this. Like the models in [41, 42], our model 3 is based on dendritic sodium spikes. In contrast to the previous models these are not generated by the very sparse recurrent CA1 interactions but by inputs from CA3. This is motivated by the fact that each CA1 PC receives about 200 afferents from other PCs, but 15000 − 30000 afferents from CA3 [55]. Still, dendritic spikes may also be generated by recurrent excitatory synapses in CA1, because they are strong, their connectivity may be appropriately patterned and they arrive at the highly excitable basal dendrites [41, 42]; CA1 and CA3 input may also cooperatively generate dendritic spikes in the basal dendrites. In model 3 with random networks, the sparse dendritic spikes enable sparse spiking of E cells in windows with little inhibitory input, while the dendritic spikes in refs. [41, 42] mediate propagation of synchrony. As already mentioned, ref. [19] found that stimulation of the local E CA1 population can excite ripple oscillations. Such stimulation will not generate dendritic spikes in the apical dendrites, but it may generate dendritic spikes in the basal dendrites of postsynaptic CA1 E cells [41, 42], which could lead to ripples in a similar manner as in our model 3 (or in the manner described in ref. [41, 42]).

We note that also the recurrent inhibitory connectivity is important for the generation of HFOs in our models: we tested in our random network models that lower values for *p*_*II*_ result in slower oscillations. Also gap junctions are frequent between inhibitory PV+BCs in CA1 [45, 113]. Experiments, however, indicate that they are not important for ripple oscillations [1, 114, 115]. Therefore we did not incorporate them in our models. In the context of interneuron ripple models, recent theoretical results show that adding gap junctions between inhibitory cells has beneficial effects for ripple oscillations, as they enhance synchrony and decrease the minimal number of cells required for ripple oscillations [116].

### Biological plausibility of our model

How realistic are our assumptions concerning the generation of dendritic spikes? Apical dendritic spikes have been directly observed during SPW/Rs [84]. It is difficult to precisely estimate the number of synaptic inputs required to generate dendritic spikes, estimates range “anywhere from a handful to dozens of inputs” [117]. The number of synaptic inputs required to evoke a dendritic spike differs between the different parts of the apical dendritic tree, in particular, it differs between apical oblique and trunk dendrites. Detailed multicompartmental models of morphologically reconstructed neurons suggested that at least ∼ 50 synaptic inputs arriving within at most 3 ms on a small part of the distal apical trunk of CA1 neuron dendrites are required to elicit dendritic sodium spikes [67, 69]. For apical oblique dendrites, it was shown that ∼ 20 inputs arriving within ∼ 6 ms suffice to generate a dendritic spike [70]. For the basal dendrites, the dendritic integration time is between 2 ms and 3 ms [68]. Dendritic spikes are generated when the input to a basal dendritic compartment is so strong that it would generate in the soma a peak depolarization of 4 mV [68]. A typical input from CA3 to CA1 evokes in the soma a peak depolarization of 0.13 mV [118], suggesting that about 30 inputs from CA3 are required to arrive within less than 3 ms at a basal dendrite to generate a dendritic spike. For an input strength of 0.4 mV as assumed in our paper, 10 inputs are required. The strength of somatic depolarization generated by dendritic spikes depends on the type of dendrite and even the branch where it has been evoked. It ranges from a few millivolts of additional somatic depolarization to 10 mV and more [68, 70, 73]. We have neglected differences in dendritic spike generation and assumed that every dendrite has the same threshold and integration time window, whereas the impact that a dendritic spike has on the soma differs. In our model 3, we assume that already 5 spikes arriving within 2 ms are sufficient for dendritic spike generation. This is similar to the number of inputs required for spike generation in basal dendrites of CA1 pyramidal cells when they receive CA1 inputs [41, 42, 54, 68]. It is also similar to the number of inputs required for spike generation in apical oblique dendrites [70] in the sense that when assuming a constant temporal distance, ∼ 20 inputs arriving within ∼ 6 ms correspond to ∼ 7 spikes in 2 ms.

Increasing the threshold for dendritic spike generation while keeping the number of afferent inputs and the dendritic integration window *w*_*D*_ constant necessitates an increase of the afferent rate. In our model 3, increasing the dendritic spike threshold from 5 to 50 would require an increase of *r*_0_ in Eq 7 from 8 Hz to more than 100 Hz to approximately maintain the same number of dendritic spikes. This value is clearly too high as a discharge rate for a typical CA3 cell during sharp waves [65] (but values larger than 10 Hz are possible [58]). The inputs from CA3 to CA1 may, however, be clustered such that sufficient coincident inputs impinge on an apical dendrite.

Our result might also indicate that a smaller number of coincident inputs than previously estimated is needed to elicit dendritic spikes. Further, dendritic spikes relevant during SPW/Rs may be less sensitive to synchrony than assumed in our model, i.e. their effective dendritic integration window may be longer. Such dendritic spikes might then be NMDA or calcium spikes [73, 84, 86, 119, 120]. Further, spikes generated on different apical oblique dendrites might sum to generate larger somatic depolarizations [70], possibly together with the slower NMDA or calcium spikes. Testing these possibilities would require a model with multiple dendrites or dendritic compartments. In S3 Appendix, we present analytical computations and numerical simulations suggesting that model 3 would still generate a similar amount of dendritic spikes (and hence lead to similar dynamics) if multiple dendritic compartments and all connections to active CA3 neurons were included. CA3 cell bursting [58] might in principle also contribute to dendritic spike generation, for example because asynchronous but overlapping bursts generate synchronous spike inputs. Experimentally found ISIs within individual CA3 bursts [58] are, however, typically larger than the sodium dendritic spike integration window [67, 68]. Finally, basal dendrites could generate dendritic spikes as incorporated in our model, since they receive inputs from CA3 PCs besides those from CA1 PCs [12]. Particularly promising candidates are the recently discovered axon carrying dendrites, from which the axon emanates in many CA1 PCs [71]. These dendrites are particularly excitable, generate strong dendritic spikes and have a high impact on action potential generation.

We implement a single dendritic compartment for simplicity and reduce the number of active inputs that it receives to cover the fact that not all CA3 input spikes interact nonlinearly. This yields a minimal model of ripple generation. We argue in S3 Appendix that in terms of numbers of dendritic spikes our minimal model with 5-fold reduction of active inputs corresponds roughly to a model where neurons have 9 dendritic compartments. If the input from CA3 during SPWRs is randomly distributed over multiple dendritic compartments, the probability of generating dendritic spikes rapidly decreases with the number of compartments, because in each compartment too few inputs arrive within the short dendritic integration time window. Clustering of synapses that are near-simultaneously activated may counteract this [121, 122]. Furthermore, dendritic compartments for dendritic spike generation may be large: Single neuron modeling studies show dendritic spike generation with only moderately clustered inputs [67]. There may also be hot spots of dendritic sodium spike generation in dendritic branching points, as observed in some neuron types [123]. In their presence, inputs to large parts of the dendrites together generate dendritic spikes.

PC spiking during SPW/Rs in our model 3 is generally very sparse, the majority of PCs contributes one to two spikes, as observed experimentally. In the model without sequence replay a few CA1 PCs spike more than 3 times during a SPW/R event (Fig 7). Such bursting is consistent with experiments [36, 58, 93, 94]. The bursts are generated in our model because the lognormal distribution of the peak dendritic currents across neurons (Eq 2) contains larger values with non-negligible probability. Additional simulations with truncated lognormal or Gaussian distributions with less bursting and simulations with a long somatic refractory period, which completely prevents multiple spiking, can generate ripple range HFOs. This shows that bursts are not necessary for the generation of HFOs in the ripple frequency range in our model.

HFOs in the ripple range occur in model 3 (Fig 8) at or before the border of stability where standard deviations across realizations increase. This is the case because a certain critical amount of externally supplied excitation (quantified by the parameters of the peak dendritic current in model 3) is needed to generate HFOs in the ripple range. Below this level, gamma and high gamma oscillatory states are reached. Beyond this level, E cell spiking cannot be organized into distinct ripple waves by PV+BC spiking anymore; the E cells are permanently active and show little oscillatory modulation in their spiking.

### Sequence generation by a two-population mechanism

A prominent property of the hippocampus is that it generates sequences of activity. These may replay previously imprinted sequential experience [94, 99, 100], serve as a backbone to store episodic memories [124, 125] or generally provide a sequential reference frame for sensory experience and brain activity [126]. Sequence generation is often assigned to CA3 because of its prominent, albeit sparse, recurrent excitatory connectivity [124, 125]. However, CA1 also generates sequences on its own [36]. Earlier work proposed that the highly sparse recurrent excitatory connectivity in CA1 could underlie sequence generation, since it may be highly structured and amplified by basal dendritic spikes [41, 42]. Here we propose a different class of models for sequence generation in CA1: two-population models. In such models the sequences are generated cooperatively by the E and I population. We conceptually propose two such models, one where the sequence generation depends on the prominent E-to-I neuron and I-to-E neuron connectivity (Fig 9A) and another one where also the similarly prominent I-to-I neuron connectivity is important (S19 Fig). We explicitly implemented the first one of these concepts, since it is more plausible for CA1. It is based on alternating pulse and gap coding of the E and I neuron populations, such that both the E and the I neuron firing patterns together generate the sequence. This is different from the classical view that sequence generation in neural networks depends mainly on the excitatory connectivity between E neuron groups like in synfire chains [50, 127–129], while inhibition prevents pathological activity, allows gating and introduces competition between sequences [130–136]. Specifically, inhibition of the embedding network and of previously active groups by propagating synfire chain activity was shown to prevent pathological, strong increases of overall network spiking activity [130, 134]; the stability of propagation along the synfire chain could then be improved by additional inhibitory neuron groups: their sparse feedforward activation induced disinhibitory removal of excessive inhibition from specific excitatory groups [136].

We have shown that two-population based sequential replay generates in model 1 high frequency oscillations with sparse E cell firing and that it is compatible with filtering of CA3 input by dendritic spikes, as proposed in model 3. Two-population based sequence generation may occur in further areas beyond the hippocampal region CA1. Promising candidates for these are areas in which there is sequence generation, rather sparse E-to-E and prominent E-to-I and I-to-E coupling. One such candidate is the hippocampal region CA3. It has been found to generate sequences [137] and studied in this function in various modeling studies [28, 124, 125, 138]. Excitatory connectivity in CA3 is very sparse (a few percent connection probability [42, 139–142]), albeit more prominent and more global than in CA1 [143, 144]. The E-to-I and I-to-E coupling is denser [139]. Observations of spontaneous (not requiring sensory stimulation and hence internally generated) sequential activity have also been made in the primary visual [145] and auditory [146] cortices as well as in the somatosensory cortex [146, 147]. Further experiments indicate that the E-to-E coupling in these areas is sparse, in the typical range for the cortex, and the E-to-I and I-to-E coupling is prominent [148–150]. To further investigate the plausibility of two- versus one-population based sequence generation schemes, future research could comparatively explore the implications of different connection probabilities, average synaptic strengths and neuron numbers (the number of I neurons is smaller than that of E neurons in all discussed areas) for the schemes.

The sequences generated by the proposed two-population mechanisms may consist of discrete groups or they might be continuous. Further, they may be preexisting or learned during experience. It is an interesting direction of future research to determine how their spontaneous formation or learning may take place through the interplay of excitatory and inhibitory synaptic plasticity [42, 151–153]. It will also be interesting to see whether there are functional, anatomical or physiological advantages when realizing sequence generation by the proposed two-population mechanisms. As an example, the generic involvement of both excitatory and inhibitory activity in sequence generation may allow to more easily avoid pathological activity like synfire chain explosions in an embedding network and extinguishing of propagation [50, 130, 134, 136].

### Experimental predictions

Ripple generation in our model 3 relies on strong sodium spikes generated in the apical dendrites of CA1 pyramidal cells. Consequently, enhancing dendritic activity by 4-aminopyridine (4-AP) or Ba^2+^ [67] should increase ripple frequency or lead to epileptoform HFOs when too much somatic spiking is induced. Conversely, selective blocking of a part of the dendritic sodium channels in the PC population’s apical dendrites should decrease the frequency of the ripple oscillations (equivalent to a decrease of *μ* and/or *σ* in our model 3, see Eq 2 and Fig 8). This may require a localized application of sodium channel blockers since the same channel subtype seems to mediate spike generation in the apical dendrites and in the axon initial segment [154].

Experiments indicate that dendritic spikes are crucial for the generation and emergence of the location specific activity of place cells [155, 156]. In particular, during exploration of a novel environment, dendritic spikes may lead for certain dendritic branches to the strengthening of clustered synapses [156] or to the strengthening of the somatic impact of their dendritic spikes [73, 157]. Based on our model 3, we predict that during later sharp wave ripple activity, such branches generate dendritic sodium spikes, possibly in conjunction with NMDA spikes [68, 73, 157]; the fast dendritic sodium spikes lead to sparse spiking of the soma (and recall of the location).

The main experimental prediction arising from our model for sequence generation is that CA1 can generate replay events intrinsically without temporally or spatially structured input from CA3 cell assemblies. For sequences of place cells, this stands in contrast to the common assumption that CA1 place fields are a reflection of spatiotemporally tuned CA3 input [1] and in contrast to the way of location recall suggested by our model 3. We predict that precisely timed interactions between E and I cells are at the heart of hippocampal replay, while couplings between local E cells are not important for this, in contrast to previous models such as [42, 158]. This implies in particular that changing PV+BC activity during SPW/Rs, e.g. by optogenetic stimulation that removes firing gaps, will impair sequence generation.

We predict sequence replay due to a certain dynamical motif in CA1: E pulse and I gap coding. For the specific case of the sequential reactivation of place cells along paths this implies that there should be E cells with place fields at locations where a group of I cells is silent. Further, the mechanism implies specific coupling motifs for the E-I and I-E connections: The I cells with silent place fields at a certain location are not innervated by E cells with place fields located shortly before in the path. Instead, they receive input from E cells with more distant place fields. In other brain regions sequences may be generated by a two-population mechanism where both E and I cells generate pulse coding, where inhibitory neurons disinhibit a group of E cells by inhibiting all others (S19A Fig). Both of these two-population mechanisms could also combine to give rise to replay.

### Conclusion

Based on neurobiological knowledge on the hippocampal regions CA1 and CA3, their single neurons and their dynamics, we have shown how CA3 drive and two-population interactions in the region CA1 may lead to the SPW/R population pattern, with its characteristic high frequency oscillations and sparse E and dense I cell firing. For the associated sequential replay we have developed a model that is consistently based on two-population interactions, specifically on E pulse and I gap coding.

## Supporting information

S1 Appendix Parameters for E and I cells, synapses, connection probabilities, initial conditions and parameters for models 1, 2 and 3 and motivation of single cell parameters. (PDF)

S2 Appendix Further details on the sequence generation in models 1 and 3 (Figs 9 and 10). (PDF)

S3 Appendix Dendritic spikes in neurons with single and multiple dendritic compartments in model 3. (PDF)

S1 Fig Synaptic dynamics in CA1. (PDF)

S2 Fig HFOs in networks with temporally broad excitation of E cells and higher I-to-E connectivity. (PDF)

S3 Fig HFOs in networks with temporally broad excitation of E cells and higher I-to-E connectivity as well as broader sharp waves. (PDF)

S4 Fig HFOs in networks with temporally broad excitation of E cells and higher I-to-I connectivity, higher I-to-E connectivity and broader sharp waves. (PDF)

S5 Fig HFOs in networks with temporally broad excitation of E cells and strong feedforward excitatory drive to I cells. (PDF)

S6 Fig Network activity for temporally broad excitation of E cells and strong feedforward excitatory drive to I cells. (PDF)

S7 Fig HFOs in networks with temporally narrow excitation of E cells and broader input pulses. (PDF)

S8 Fig HFOs in networks with temporally narrow excitation of E cells and larger I-to-E and I-to-I connectivity. (PDF)

S9 Fig HFOs in networks with temporally narrow excitation of E cells and broader spread of individual input pulses as well as higher I-to-E and I-to-I connectivity. (PDF)

S10 Fig HFOs in networks with temporally narrow excitation of E cells and broader spread of individual input pulses as well as higher I-to-E connectivity. (PDF)

S11 Fig HFOs in networks incorporating dendritic excitation and higher I-to-E connection probability. (PDF)

S12 Fig HFOs in networks incorporating dendritic excitation and higher I-to-E synaptic latency. (PDF)

S13 Fig HFOs in networks incorporating dendritic excitation and lower E-to-I peak conductance. (PDF)

S14 Fig HFOs in networks incorporating dendritic excitation and higher I-to-E latency, faster E-to-I rise time and lower E-to-I peak conductance. (PDF)

S15 Fig HFOs in networks incorporating dendritic excitation and a truncated distribution for the peak dendritic current. (PDF)

S16 Fig HFOs in networks incorporating dendritic excitation and a truncated re-sampled distribution for the peak dendritic current as well as lower E-to-I peak conductance. (PDF)

S17 Fig HFOs in networks incorporating dendritic excitation and a Gaussian distribution for the peak dendritic current as well as lower E-to-I peak conductance. (PDF)

S18 Fig HFOs in networks incorporating dendritic excitation and a long somatic absolute refractory period for the E cells. (PDF)

S19 Fig Further alternative concepts of replay. (PDF)
