# Supplementary material for: High-frequency oscillations and sequence generation in two-population models of hippocampal region CA1
Source: PLoS Comput Biol. 2022 Feb 17;18(2):e1009891. doi: 10.1371/journal.pcbi.1009891 (PMC8890743; doi:10.1371/journal.pcbi.1009891)
Supplement: S1 Appendix — (PDF) [file pcbi.1009891.s001.pdf]

# S1 Appendix

## Parameters of cells and synapses

Table 1: **Parameters of E cells.**

| | | | |
|---|---|---|---|
| $C_E$ | 275 pF | $g_l^E$ | 25 nS |
| $E_{\text{rest}}^E$ | −67 mV | $E_{\text{exc}}^E$ | 0 mV |
| $E_{\text{inh}}^E$ | −68 mV | $N_E$ | 12000 |
| $V_{\text{thresh}}^E$ | −50 mV | $V_{\text{reset}}^E$ | −67 mV |
| $\tau_{\text{ref}}^E$ | 2 ms | | |

Table 2: **Parameters of I cells.**

| | | | |
|---|---|---|---|
| $C_I$ | 100 pF | $g_l^I$ | 10 nS |
| $E_{\text{rest}}^I$ | −65 mV | $E_{\text{exc}}^I$ | 0 mV |
| $E_{\text{inh}}^I$ | −75 mV | $N_I$ | 200 |
| $V_{\text{thresh}}^I$ | −52 mV | $V_{\text{reset}}^I$ | −65 mV |
| $\tau_{\text{ref}}^I$ | 1 ms | | |

Table 3: **Parameters of synapses on E cells.**

| | | | |
|---|---|---|---|
| $g_{\text{exc,peak}}^E$ | 0.9 nS | $g_{\text{inh,peak}}^E$ | 9.0 nS |
| $\tau_{\text{exc},d}^E$ | 1.8 ms | $\tau_{\text{exc},r}^E$ | 0.5 ms |
| $\tau_{\text{inh},d}^E$ | 2.0 ms | $\tau_{\text{inh},r}^E$ | 0.4 ms |

Table 4: **Parameters of synapses on I cells.**

| | | | |
|---|---|---|---|
| $g_{\text{exc,peak}}^I$ | 3.0 nS | $g_{\text{inh,peak}}^I$ | 5.0 nS |
| $\tau_{\text{exc},d}^I$ | 1.2 ms | $\tau_{\text{exc},r}^I$ | 0.5 ms |
| $\tau_{\text{inh},d}^I$ | 1.2 ms | $\tau_{\text{inh},r}^I$ | 0.45 ms |

Table 5: **Connection probabilities in CA1.**

| | | | |
|---|---|---|---|
| $p_{EE}$ | 1.64% | $p_{II}$ | 20% |
| $p_{IE}$ | 10% | $p_{EI}$ | 10% |

The latencies $\tau_l$ are fixed at 1 ms for all four types of synapses if not mentioned otherwise. At the beginning of a simulation, the neurons are initialized with voltages drawn from Gaussian distributions centered around their respective resting potentials with standard deviation 0.1 mV.

## Parameters specific to models 1-3

Table 6: **Parameters for model 1**

| | | | |
|---|---|---|---|
| $t_0$ | 50 ms | $\sigma_g$ | 10 ms |
| $\mathrm{CV}_g$ | 0.5 | $g^0_{\mathrm{ext}}$ | $\sim \mathcal{N}(\bar{g}, \mathrm{CV}_g \bar{g})$ |

Table 7: **Parameters for model 2**

| | | | |
|---|---|---|---|
| $t_0$ | $\sim \mathcal{N}(50\ \mathrm{ms}, \sigma_t)$ | $\sigma_t$ | 10 ms |
| $\sigma_g$ | 3 ms | $g^0_{\mathrm{ext}}$ | $\bar{g}$ |

Table 8: **Parameters for model 3**

| | | | |
|---|---|---|---|
| $r_0$ | 8 Hz | $\sigma_t$ | 10 ms |
| $N_{E,\mathrm{CA3}}$ | 15000 | $p_0$ | $\frac{130}{N_{E,\mathrm{CA3}}}$ |
| $w_D$ | 2 ms | $t_{r,D}$ | 5 ms |
| $n_D$ | 5 | | |
| dendritic current rise time | 1 ms | dendritic current decay time | 4 ms |

## Single cell parameters

We model single neurons as conductance-based leaky integrate-and-fire neurons. As parameters we have chosen standard values for CA1: For the I cell model, the time constant is 10 ms, in good agreement with values reported by [1] (Table 1). The firing threshold for our I cells is $-52$ mV, also in good agreement with the values from [1] (Table 1). In agreement with these values, in [2], membrane time constants for basket cells of $9.9 \pm 4.6$ ms are reported. Our model I cell resting membrane potential is also in good agreement with values reported in [2] ($-65$ mV in our model vs. $-64 \pm 7.2$ mV in Buhl et al. 1996).

For our E cell model, the membrane time constant is 11 ms, in good agreement with values reported by [3] (Fig. S1). Also the firing threshold and the resting membrane potential ($-67$ mV) are in good agreement with values reported by [3] (Fig. S5). Similar values for CA1 E cell resting potentials ($-66$ mV compared to $-67$ mV in our model) are reported in [4]. Membrane time constants for CA1 pyramidal cells between 5.4 and 18.2 ms are reported in [5]. In this study, resting potentials between $-60$ and $-75$ mV are reported; our value ($-67$ mV) fits well into this range. Overall, our single cell model parameters are in good agreement with experimental observations and with previous theoretical work, e.g. [6].

## References


1. Afia B. Ali, Jim Deuchars, Hannelore Pawelzik, and Alex M. Thomson. CA1 pyramidal to basket and bistratified cell epsps: dual intracellular recordings in rat hippocampal slices. *The Journal of Physiology*, 507(1):201–217, 1998.

2. Eberhard H. Buhl, Tibor Szilágyi, Katalin Halasy, and Peter Somogyi. Physiological properties of anatomically identified basket and bistratified cells in the ca1 area of the rat hippocampus in vitro. *Hippocampus*, 6(3):294–305, 1996.

3. Florian Bähner, Elisa K. Weiss, Gunnar Birke, Nikolaus Maier, Dietmar Schmitz, Uwe Rudolph, Michael Frotscher, Roger D. Traub, Martin Both, and Andreas Draguhn. Cellular correlate of assembly formation in oscillating hippocampal networks in vitro. *Proceedings of the National Academy of Sciences*, 108(35):14385–14386, 2011.

4. Nathan P. Staff, Hae-Yoon Jung, Tara Thiagarajan, Michael Yao, and Nelson Spruston. Resting and active properties of pyramidal neurons in subiculum and ca1 of rat hippocampus. *Journal of Neurophysiology*, 84(5):2398–2408, 2000. PMID: 11067982.

5. J. Deuchars and A.M. Thomson. Ca1 pyramid-pyramid connections in rat hippocampus in vitro: Dual intracellular recordings with biocytin filling. *Neuroscience*, 74(4):1009 – 1018, 1996.

6. José R. Donoso, Dietmar Schmitz, Nikolaus Maier, and Richard Kempter. Hippocampal ripple oscillations and inhibition-first network models: Frequency dynamics and response to gaba modulators. *Journal of Neuroscience*, 2018.
