# Supplementary material for: High-frequency oscillations and sequence generation in two-population models of hippocampal region CA1
Source: PLoS Comput Biol. 2022 Feb 17;18(2):e1009891. doi: 10.1371/journal.pcbi.1009891 (PMC8890743; doi:10.1371/journal.pcbi.1009891)
Supplement: S2 Appendix — (PDF) [file pcbi.1009891.s002.pdf]

S2 Appendix

Further details on the sequence generation in models 1 and 3 (Figs 9 and 10).
For both model 1 and model 3, the E-to-E connectivity is unchanged, i.e. it is random and such that each E cell receives on average 197 connections from other E cells. Likewise, the I-to-I connectivity is random and such that each I cell receives on average 40 connections from other I cells.
Parameters for replay in model 1. The parameters are as in Fig 4, except for those mentioned below. The reference values from Fig 4 are given in parentheses below. AMPA peak conductance on I cells:
$g^{I}_{\text{exc,peak}} = 1.0$ (3.0) nS
Parameters of the initializing Gaussian (cf. Eq 6) pulse input (magenta): $t_0 = 37$ ms, $\sigma_g = 4.5$ ms, $\text{CV}_g = 0.1$ and $(\bar{g}, n_E) = (20 \text{ nS}, 50)$.
Parameters of the main sharp wave input (black): $t_0 = 56$ ms, $\sigma_g = 13$ ms, $\text{CV}_g = 0.1$ and $(\bar{g}, n_E) = (24 \text{ nS}, 400)$
Parameters for replay in model 3.
The parameters are as in Fig 8, except those mentioned below. The reference values from Fig 8 are given in parentheses. We keep the I-to-E synaptic latency of 0.5 ms as in Fig 8. This comparably small value is helpful for stable replay accompanied by HFOs in the ripple range. There are $N_{E,\text{CA3}} = 15000$ neurons in both the initializing (magenta) and main (black) group. The CA3-CA1 excitation is exclusively via dendritic spikes, i.e. there are no small depolarizations elicited by each CA3 spike as in Fig 8.
AMPA peak conductance on I cells: $g^{I}_{\text{exc,peak}} = 1.0$ (3.0) nS
Parameters of the peak dendritic current distribution (cf. Eq 2): $\mu = -0.15$ (0.0), $\sigma = 0.05$ (0.75). The distribution is truncated at 4 nA. After drawing from the distribution, 92% of all values are thus mapped to 0.
Rectangular rate functions for all CA3 neurons: $r(t) = r_0\,(\Theta(t - (t_0 - \sigma)) - \Theta(t - (t_0 + \sigma)))$ with the Heaviside function Θ. The rate function of the CA3 neurons driving group E_0 is centered at $t_0 = 37$ ms and has width $\sigma = 4.5$ ms (magenta spikes Fig 10 A top). The rate function of the CA3 neurons driving all groups except E_0 is centered at $t_0 = 56$ ms and has width $\sigma = 16$ ms (black spikes in Fig 10 A top). The peak rate $r_0 = 8$ Hz.
CA3-CA1 connectivity: $p = \frac{300}{N_{E,\text{CA3}}}$ $(\frac{130}{N_{E,\text{CA3}}})$.
Justification of parameter changes for replay in model 3 (Fig 10) We here justify the parameter changes in Fig 10 compared to Fig 8 one-by-one, subsuming the changes for μ and σ under one bullet point.

1. The E-to-I peak conductance has to be decreased to reduce the spiking activity of the I cells. This is similar to SI Fig. 14, where we have shown that decreasing the E-to-I peak conductance results in faster HFOs because of reduced I cell activity.

2. We decrease μ from 0 to -0.15 and σ from 0.75 to 0.05. As a result, the mean of the lognormal distribution for the peak dendritic current decreases from 1.32 nA to 0.86 nA. The standard deviation of the distribution decreases from 1.15 nA to 0.04 nA. This is necessary to decrease the amount of depolarization a dendritic spikes evokes in the soma. With the obtained weaker, yet sufficiently strong dendritic spikes, an E cell that generates a dendritic spike can still generate an action potential but after the action potential is generated, further firing will be prevented by inhibition.

3. Increasing the CA3-to-CA1 connection probability results in more inputs to all CA1 cells on average, so that more dendritic spikes will be generated. Because these are weak (see point 2), they do not usually lead to somatic spikes. Instead, the input is dominated by inhibition unless an E cell receives less inhibition because of a gap in I cell firing (Fig. 9 A). In this gap, the E cell has the opportunity to spike.

4. Because there are more dendritic spikes due to the increased CA3-to-CA1 connection probability, more E cells will participate in every ripple cycle. This, however, would lead to too much I cell spiking. Therefore, we set the majority of peak dendritic currents to 0, such that only a small fraction (at most 8%) of all CA1 E cells can spike. This is similar to models 1 and 2, where only a fraction of all E cells $(\frac{n_E}{N_E})$ receives CA3 excitation. We find that keeping the CA3-CA1 connection probability at the lower value used in Fig. 8 and then setting fewer of the peak dendritic currents to zero results in less stable replay. This is because the smaller CA3-to-CA1 connection probability implies that for otherwise suitable parameters, dendritic spikes are generated more sparsely and randomly. Therefore, E cells can spike at time points outside of the time slot allocated to them. Finally, we note that there is a trade-off between how many of the peak dendritic current values are set to 0 and the CA3-to-CA1 connection probability: within narrow bounds, decreasing the CA3-to-CA1 connection probability can be compensated by increasing the number of E cells that are excited by CA3.
