# Supplementary material for: High-frequency oscillations and sequence generation in two-population models of hippocampal region CA1
Source: PLoS Comput Biol. 2022 Feb 17;18(2):e1009891. doi: 10.1371/journal.pcbi.1009891 (PMC8890743; doi:10.1371/journal.pcbi.1009891)
Supplement: S3 Appendix — (PDF) [file pcbi.1009891.s003.pdf]

S3 Appendix

Dendritic spikes in neurons with single and multiple dendritic compartments in model 3. How many dendritic spikes are generated in model 3? Would the model work with multiple dendritic compartments? To address these questions, we perform additional numerical simulations and analytical computations. Fig. 1 shows the obtained estimates of the number of dendritic spikes generated with different numbers of dendritic branches and input connections.

In the analytical computations we neglect the refractory period of dendritic spikes and we bin time in bins of size dt; we assume that CA3 synapses contact each branch with the same probability, that each CA1 PC has the same number of presynaptic contacts and that the inputs of different CA1 PCs are independent. The ith time bin is $(t_i - dt, t_i]$ with $t_i = idt$. Inputs arriving within the dendritic window w_D before and during the bin, $(t_i - w_D, t_i]$, are taken into account for dendritic spike generation within the bin. How shall we choose the bin size? If it is too small, the dendritic integration windows have many and large overlaps. Therefore a time interval in which sufficiently many inputs are received leads to dendritic spiking in several close-by bins that cover the interval: consider as an extreme example the case of infinitesimally small dt, where spiking in infinitely many bins occurs if n_D spikes arrive anywhere within a time interval of w_D. On the other hand, the bin size may not be too large, because then too many groups of n_D or more spikes arriving within a time interval of less than w_D do not arrive within a time bin, but only in separate ones and are thus not accounted for dendritic spike generation. By comparison with numerical simulations we find that a bin size of $dt = 1$ ms $= w_D/2$ is a good compromise that results in reasonable agreement between the overall generated number of dendritic spikes in the simplified system and in the full numerical simulations, see Fig. 1A.

The choice of bin size means, in particular, that we assume that dendritic spikes are usually generated due to inputs spread over more than $dt = w_D/2$. In other words, we assume that the probability that sufficiently many inputs for spike generation have arrived already in the first half of the window $(t_i - w_D, t_i]$ is small. If the probability was large, we would often have the case that inputs generate a spike in two subsequent bins, inconsistent with the refractory period in the full model. (The input in the discussed first half of the window, $(t_i - w_D, t_i - \frac{1}{2}w_D]$, counts for both $(t_i - w_D, t_i]$ and $(t_{i-1} - w_D, t_{i-1}] = (t_i - \frac{3}{2}w_D, t_i - \frac{1}{2}w_D]$; therefore we would have a dendritic spike in $(t_{i-1} - dt, t_{i-1}]$ and $(t_i - dt, t_i]$.) To show the plausibility of the assumption and thus further justify the choice of bin size, we consider an isolated dendritic integration window and compute the probability of dendritic spike generation in its first half given that the input in the full window suffices to generate a dendritic spike,

$$
\begin{aligned}
&P(\text{dendritic spike generation in first half } \mid \text{dendritic spike generation}) && (1)\\
=&P(\#\text{inputs in first half} \geq n_D \mid \#\text{inputs in entire window} \geq n_D) && (2)\\
=&\frac{P((\#\text{inputs in first half} \geq n_D) \wedge (\ \#\text{inputs in entire window} \geq n_D))}{\text{P}(\#\text{inputs in entire window} \geq n_D)} && (3)\\
=&\frac{P(\#\text{inputs in first half} \geq n_D)}{\text{P}(\#\text{inputs in entire window} \geq n_D)} && (4)\\
=&\frac{\sum_{i=n_D}^{\infty} P(i \text{ inputs in the first half})}{\sum_{j=n_D}^{\infty} P(j \text{ inputs in the entire window})} && (5)\\
\approx&\frac{1 - \text{CDF}_{n_{\text{pre}} r(t_c) \frac{w_D}{2}}(n_D - 1)}{1 - \text{CDF}_{n_{\text{pre}} r(t_c) w_D}(n_D - 1)}, && (6)
\end{aligned}
$$

where "#" denotes "number of", t_c is the center of the integration window, n_D the threshold for dendritic spike generation and w_D the length of the window. We use in the fourth line that $P((\#\text{inputs in first half} \geq n_D) \wedge (\#\text{inputs in entire window} \geq n_D)) = P(\#\text{inputs in first half} \geq n_D)$, because if we have n_D or more input arrivals in the first half, this already implies that we also have n_D or more arrivals in the entire dendritic integration window. In the sixth line, we use that the number of inputs during the first half of the dendritic integration window and during the entire dendritic integration window are Poisson distributed, with an approximate parameter $\lambda = n_{\text{pre}} r(t_c) \frac{w_D}{2}$ and $\lambda = n_{\text{pre}} r(t_c) w_D$, respectively. Here $r(t_c)$ is the rate of a CA3 neuron at the center of the window and $\text{CDF}_\lambda(k) = \sum_{i=0}^{k} \frac{\lambda^k}{k!} e^{-\lambda}$ is the cumulative distribution function of the Poisson distribution. Inserting our parameters ($n_D = 5, r(t) = r_0 \exp[-(t - t_0)^2/(2\sigma_t^2)]$ with $r_0 = 8$ Hz, $w_D = 2$ ms, $n_{\text{pre}} = n_{\text{pre},0} = 130$) shows that the fraction of dendritic spikes generated in the first half of the window remains below 7.2% of all dendritic spikes, where the maximum (about 7.2%) is assumed for $t_c = t_0$. Fig. 1 shows that despite the simplifying assumptions, the numbers of spikes obtained numerically with the reduced, time-binned system agree reasonably well with the results of full network simulations (cf. main text

Fig. 7). As an example, the simulation of the simplified system displayed in Fig. 1A (red circles) yielded 9510 dendritic spikes, the full network simulation 10492 dendritic spikes (green histogram).
We now further simplify the system for analytical assessment. First, we assume independent inputs in the different dendritic integration windows $(t_i - w_D, t_i]$. Second, we again assume that the input rate of a CA3 neuron during the window is given by the input rate $r(t)$ at the window's center $t_{ci} = t_i - \frac{1}{2}w_D$, where $r(t_{ci}) = r_0 \exp(-(t_{ci} - t_0)^2/(2\sigma_t^2))$. The number of inputs within the window is then Poisson distributed with parameter $\lambda = n_{\mathrm{pre}} r(t_{ci}) w_D$. A CA1 neuron generates a dendritic spike in bin $(t_i - dt, t_i]$, if the number of inputs in $(t_i - w_D, t_i]$ exceeds $n_D - 1$. This happens with probability

$$P_{\mathrm{DS}}(i) = 1 - \mathrm{CDF}_{n_{\mathrm{pre}} r(t_{ci}) w_D}(n_D - 1), \tag{7}$$

where again CDF denotes the cumulative distribution function of the Poisson distribution. The expected number of dendritic spikes generated in the entire CA1 population in the ith time bin is therefore

$$\mu_{\mathrm{DS}}(i) = N_E P_{\mathrm{DS}}(i). \tag{8}$$

Fig. 1A shows the analytically derived expectation values, which agree well with the sampled numbers. The continuous curve connecting the values for illustration is the interpolation
$\mu_{\mathrm{DS}}(t) = N_E P_{\mathrm{DS}}(t) = N_E \left(1 - \mathrm{CDF}_{n_{\mathrm{pre}} r(t-dt/2) w_D}(n_D - 1)\right)$.
We use our analytical estimation to investigate the impact of dendritic compartments and additional inputs on the number of generated dendritic spikes. For this, we assume that the inputs n_{pre} are distributed equally between the m branches of a neuron, i.e. each branch receives n_{pre}/m inputs (assuming for simplicity that n_{pre} is divisible by m). The number of inputs in a given dendrite in the ith integration window is then Poisson distributed with mean $\lambda_D(i) = \frac{n_{\mathrm{pre}}}{m} r(t_{ci}) w_D$. The probability of generating a dendritic spike in this dendrite is

$$P_{\mathrm{DS}}(i) = 1 - \mathrm{CDF}_{\frac{n_{\mathrm{pre}}}{m} r(t_{ci}) w_D}(n_D - 1) \tag{9}$$

and the expected number of dendritic spikes in the entire population

$$\mu_{\mathrm{DS}}(i) = N_E m P_{\mathrm{DS}}(i), \tag{10}$$

because each of the m dendritic compartments of a neuron can generate a dendritic spike. Fig. 1B exemplarily illustrates how μ_{DS} strongly increases and decreases with n_{pre} and m, respectively. Eq. (10) allows to compute the increase in the number of inputs n_{pre} required to compensate the loss of dendritic spikes with increasing m: For this we equate Eq. (10) with our standard parameters ($m = 1$, $n_{\mathrm{pre}} = n_{\mathrm{pre},0} = 130$) and Eq. (10) with fixed $m > 1$ and variable n_{pre}. We then numerically solve for n_{pre}. We perform this computation for the window around the central peak of the equation, $t_{ci} = t_0 = 50$ ms, where most dendritic spikes are generated, as well as for the windows around $t_{ci} = 40$ ms and $t_{ci} = 30$ ms, see Fig. 1C. The computation indicates, for example, that with 9 dendritic compartments an about 5-fold increase in the number of inputs per neuron (using that on average 130 presynaptic CA3 neurons send a spike, see Materials and methods, Model 3) yields the same number of dendritic spikes in the CA1 population, as further illustrated in Fig. 1D. We find in full network simulations that already a 4.4-fold increase in the number of inputs per neuron suffices to generate the same overall number of dendritic spikes in the CA1 population as for standard parameters (cf main text Fig. 7). This might be because the refractory period in the full network simulations affects systems with multiple dendritic compartments less, due to the lower spike density in a single compartment. The analytics also shows that an increased w_D has a similar effect as an increased n_{pre}. Given the experimental evidence ($2 \leq w_D < 3$ ms [1, 2], or $w_D \sim 6$ ms [3]), we have chosen a rather small w_D (albeit also a small n_D). A longer dendritic integration window may thus also compensate dendritic compartments. Taken together, our considerations indicate that we expect in networks of model 3 dendritic spikes during SPW/Rs also with a moderate number of dendritic compartments.

References

1. Alon Polsky, Bartlett W Mel, and Jackie Schiller. Computational subunits in thin dendrites of pyramidal cells. *Nature Neuroscience*, 7:621– 627, 2004.

2. Sonia Gasparini, Michele Migliore, and Jeffrey C. Magee. On the initiation and propagation of dendritic spikes in CA1 pyramidal neurons. *Journal of Neuroscience*, 24(49):11046–11056, 2004.

3. Attila Losonczy and Jeffrey C. Magee. Integrative properties of radial oblique dendrites in hippocampal CA1 pyramidal neurons. *Neuron*, 50(2):291 – 307, 2006.

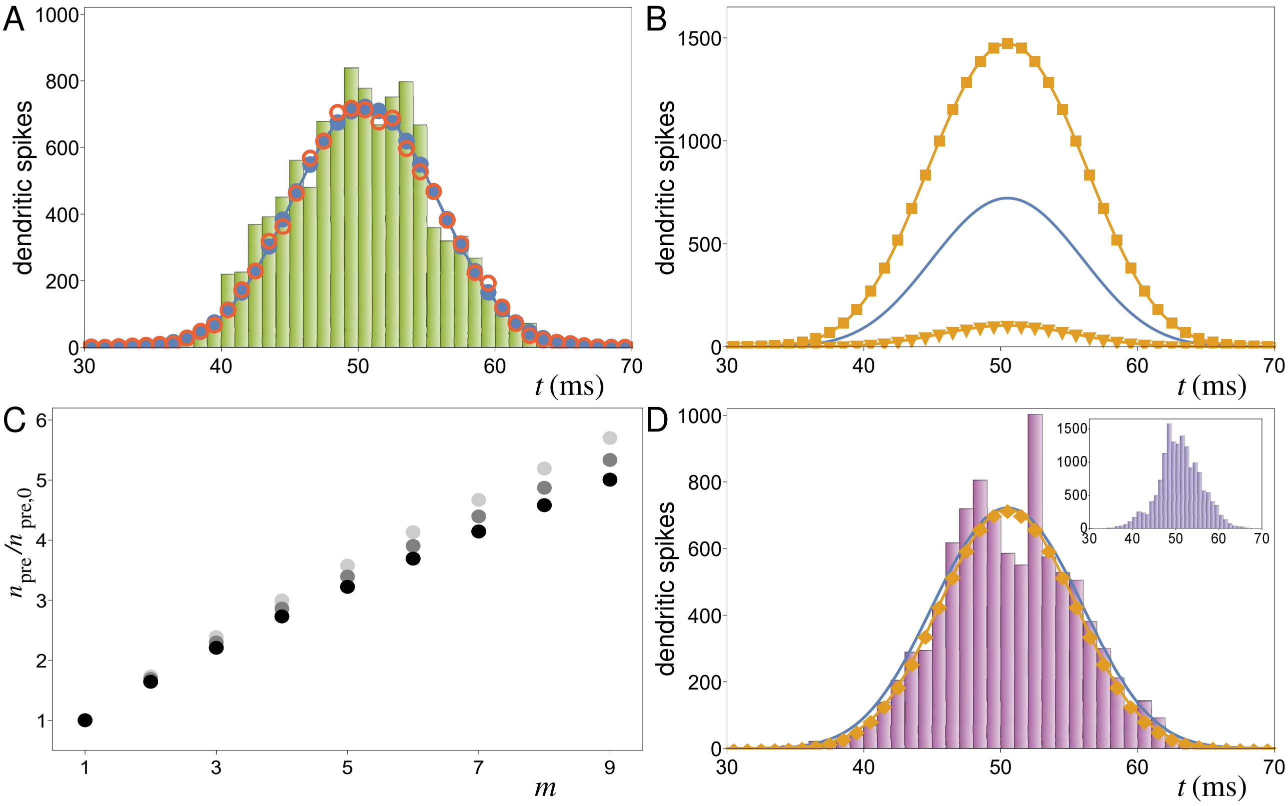

Figure 1: **Number of dendritic spikes in CA1 populations with one and multiple dendrites.** A: Number of dendritic spikes generated in time bins of 1 ms duration in a simulation of the full model 3 network with parameters as in main text Fig. 7 (green histogram), in a simulation of the reduced model with binned time (red circles) and expectation values obtained from an analytical computation using the reduced model (blue circles). In a given bin, only a small fraction of the $N_E = 12000$ CA1 neurons generates dendritic spikes. This underlies the sparse spiking in model 3. B: An increased number of input connections from CA3 yields an increased number of dendritic spikes (yellow squares and interpolating curve: $n_{\text{pre}} = 1.25 n_{\text{pre},0}$ input connections per CA1 PC, analytical expectation values obtained with the reduced model; blue curve: interpolating curve for $n_{\text{pre}} = n_{\text{pre},0} = 130$ as in A, displayed for comparison). An increased number of dendritic compartments yields a decreased number of dendritic spikes (yellow triangles and interpolating curve: $n_{\text{pre}} = n_{\text{pre},0}$ and $m = 2$ dendritic compartments per neuron, analytical expectation values obtained with the reduced model). C: Increase in the number of input connections n_{pre} compensating an increased number of dendritic compartments. The figure shows the factor $n_{\text{pre}}/n_{\text{pre},0}$ by which the standard number $n_{\text{pre},0} = 130$ of input connections has to be increased to reach the same number of dendritic spikes as for $m = 1$ in the 31th (light gray points), 41th (gray points) and 51th (black points) time bins, which end at $t_{30} = 31$ms, $t_{40} = 41$ms and $t_{50} = 51$ms. D: Number of dendritic spikes for $m = 9$ dendritic compartments and a compensatorily increased number n_{pre} of input connections. The analytic estimates of the number of dendritic spikes in the CA1 population with $n_{\text{pre}} = 5 n_{\text{pre},0}$ and $m = 9$ (yellow diamonds and interpolating curve) agree well with those for $n_{\text{pre}} = n_{\text{pre},0}$ and $m = 1$ (blue interpolating curve). In our full network simulations, a 4.4-fold increase of the number of inputs yields similar numbers of dendritic spikes (magenta histogram, $n_{\text{pre}}/n_{\text{pre},0} = 4.4$, $m = 9$, other parameters are as in main text Fig. 7). A 5-fold increase of the number of inputs overcompensates the additional dendritic compartments, generating more dendritic spikes (blue-magenta histogram in the inset).
