# Supplementary material for: High-frequency oscillations and sequence generation in two-population models of hippocampal region CA1
Source: PLoS Comput Biol. 2022 Feb 17;18(2):e1009891. doi: 10.1371/journal.pcbi.1009891 (PMC8890743; doi:10.1371/journal.pcbi.1009891)

S1 Fig

Synaptic dynamics in CA1 The figure displays somatic voltage, conductance and current changes due to excitatory and inhibitory inputs mediated by the four synapse classes present in our models, for the standard parameters used. At $t = 2$ ms (red vertical dashed line) a presynaptic spike occurs which triggers a conductance change $\tau_l = 1$ ms later. Membrane voltage (top panel), synaptic conductance (middle panel) and post-synaptic current (PSC, bottom panel) for (A): E-to-E synapses, (B): I-to-E synapses, (C): E-to-I synapses, (D): I-to-I synapses. Holding potential: -55 mV. $\sigma_n = 0$ mV (no noise on membrane voltage, see Eq 1). Values for time constants and peak conductances can be found in S1 Appendix.

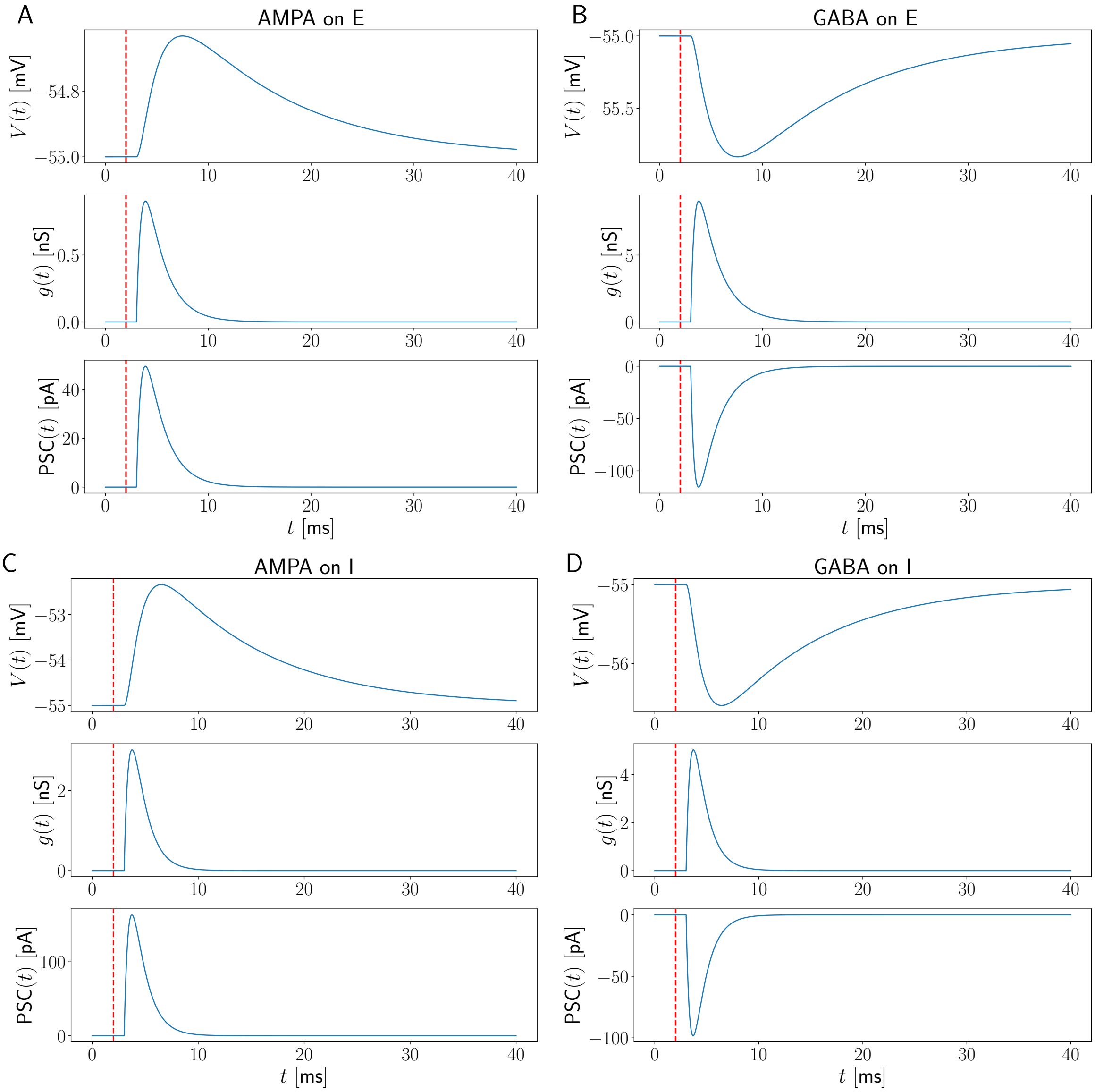

Supplement: S1 Fig — (PDF) [file pcbi.1009891.s004.pdf]
