# Supplementary material for: High-frequency oscillations and sequence generation in two-population models of hippocampal region CA1
Source: PLoS Comput Biol. 2022 Feb 17;18(2):e1009891. doi: 10.1371/journal.pcbi.1009891 (PMC8890743; doi:10.1371/journal.pcbi.1009891)

S2 Fig

HFOs in networks with temporally broad excitation of E cells and higher I-to-E connectivity. Parameters are as in Fig 4, except higher $p_{IE} = 0.2$. The displayed frequency range for f_I and f_E is enlarged to $[100, 200]$ Hz to yield a broader overview. The plot layout is as in Fig 4.

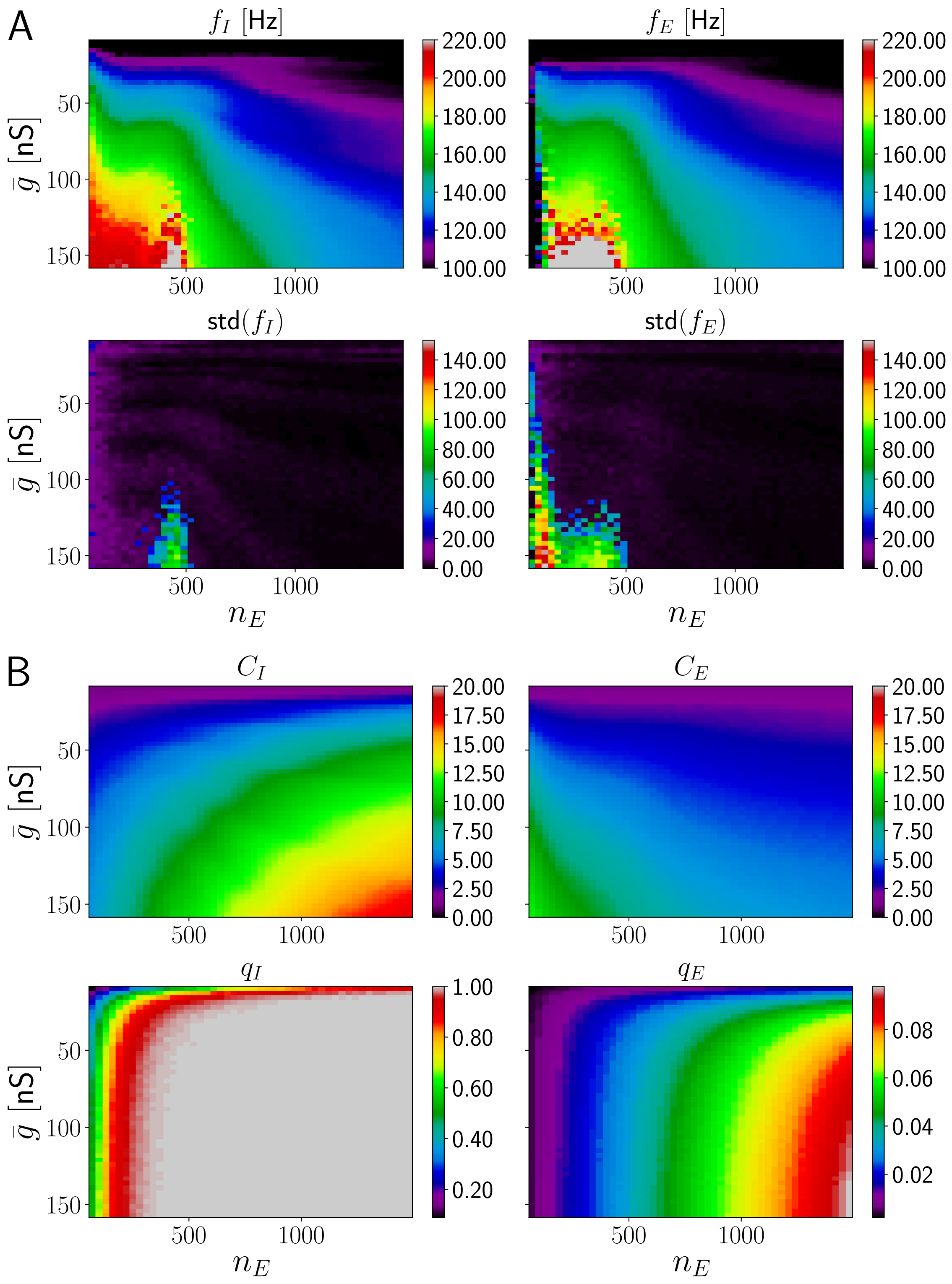

Supplement: S2 Fig — (PDF) [file pcbi.1009891.s005.pdf]
