# Supplementary material for: High-frequency oscillations and sequence generation in two-population models of hippocampal region CA1
Source: PLoS Comput Biol. 2022 Feb 17;18(2):e1009891. doi: 10.1371/journal.pcbi.1009891 (PMC8890743; doi:10.1371/journal.pcbi.1009891)

S3 Fig

HFOs in networks with temporally broad excitation of E cells and higher I-to-E connectivity as well as broader sharp waves. Parameters are as in Fig 4, except higher $p_{IE} = 0.3$ and larger width of sharp waves $\sigma_g = 15$ ms. The plot layout is as in Fig 4. The displayed frequency range for f_I and f_E is enlarged to $[100, 200]$ Hz to yield a broader overview.

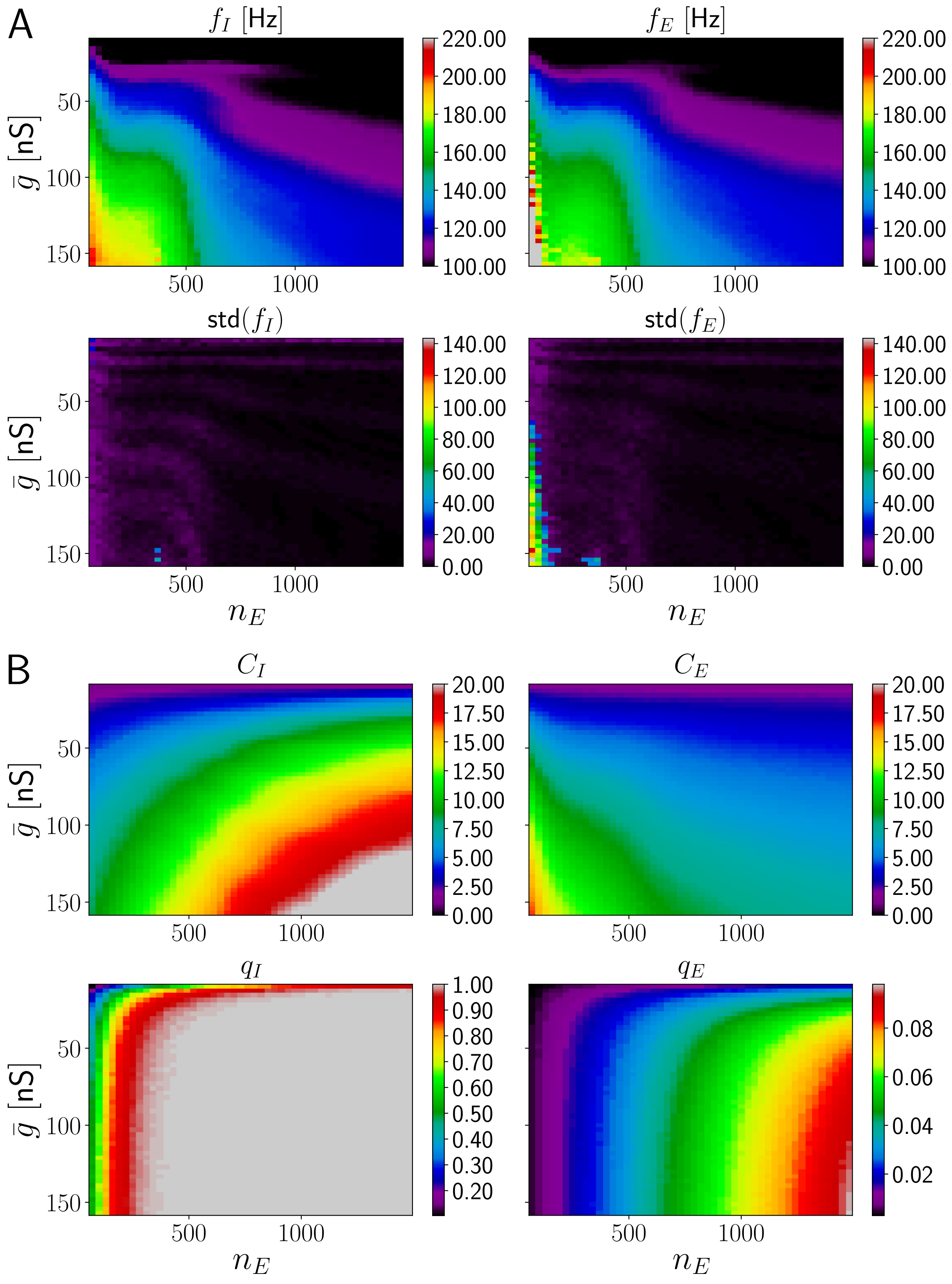

Supplement: S3 Fig — (PDF) [file pcbi.1009891.s006.pdf]
