# Supplementary material for: High-frequency oscillations and sequence generation in two-population models of hippocampal region CA1
Source: PLoS Comput Biol. 2022 Feb 17;18(2):e1009891. doi: 10.1371/journal.pcbi.1009891 (PMC8890743; doi:10.1371/journal.pcbi.1009891)

S5 Fig

HFOs in networks with temporally broad excitation of E cells and strong feedforward excitatory drive to I cells. A, upper subpanel: Frequency of I and E population activity oscillations f_I, f_E as a function of mean drive $\bar{g}$ and number of excited E cells n_E (average taken across different network realizations). Lower: Corresponding standard deviations across network realizations, std(f_I) and std(f_E). B, upper subpanels: mean number of spikes per active inhibitory (excitatory) neuron C_I (C_E). Lower: fraction of active neurons (q_I and q_E). The range for f_I and f_E is $[140, 200]$ Hz. The ranges for C_I and C_E are $[0, 20]$ and $[0, 3]$, respectively. Frequency values and spike numbers below (above) this range are indicated in black (gray). The white horizontal line is located at $\bar{g} = 25$ nS, roughly delimiting the region of sparse E firing. The white circle is at $(\bar{g}, n_E) = (19 \text{ nS}, 190)$ (parameters of S7 Figure). Parameters as in Fig 4, except for drive of I cells: Each PV+BC is driven by a conductance as in Eq 6, with width $\sigma_g = 10$ ms and fixed amplitude $\bar{g} = 20$ nS. The plot layout is as in Fig 4.

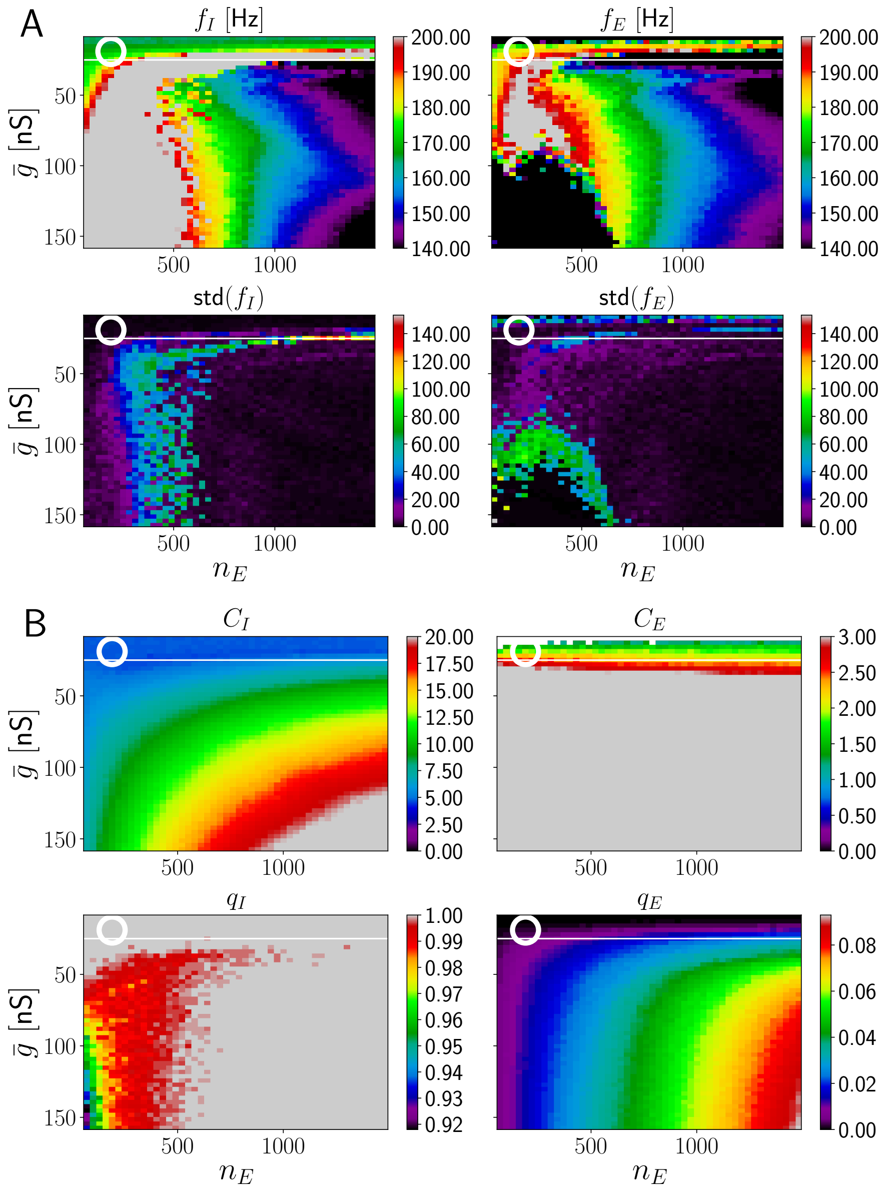

Supplement: S5 Fig — (PDF) [file pcbi.1009891.s008.pdf]
