# Supplementary material for: High-frequency oscillations and sequence generation in two-population models of hippocampal region CA1
Source: PLoS Comput Biol. 2022 Feb 17;18(2):e1009891. doi: 10.1371/journal.pcbi.1009891 (PMC8890743; doi:10.1371/journal.pcbi.1009891)

S6 Fig

Network activity for temporally broad excitation of E cells and strong feedforward excitatory drive to I cells. A: Sharp wave time courses for E cells, spike rastergrams and network activities. The I cells receive sharp wave input with fixed amplitude $\bar{g} = 20$ nS and $\sigma_g = 10$ ms. The horizontal red dashed line is located at the mean $\bar{g}$ of the sharp wave amplitudes. B: Histograms of spike counts for E (left) and I (right) cells on a logarithmic scale. Parameter values as in S6 Figure, parameters of E cell drive are $(\bar{g}, n_E) = (19 \text{ nS}, 190)$.

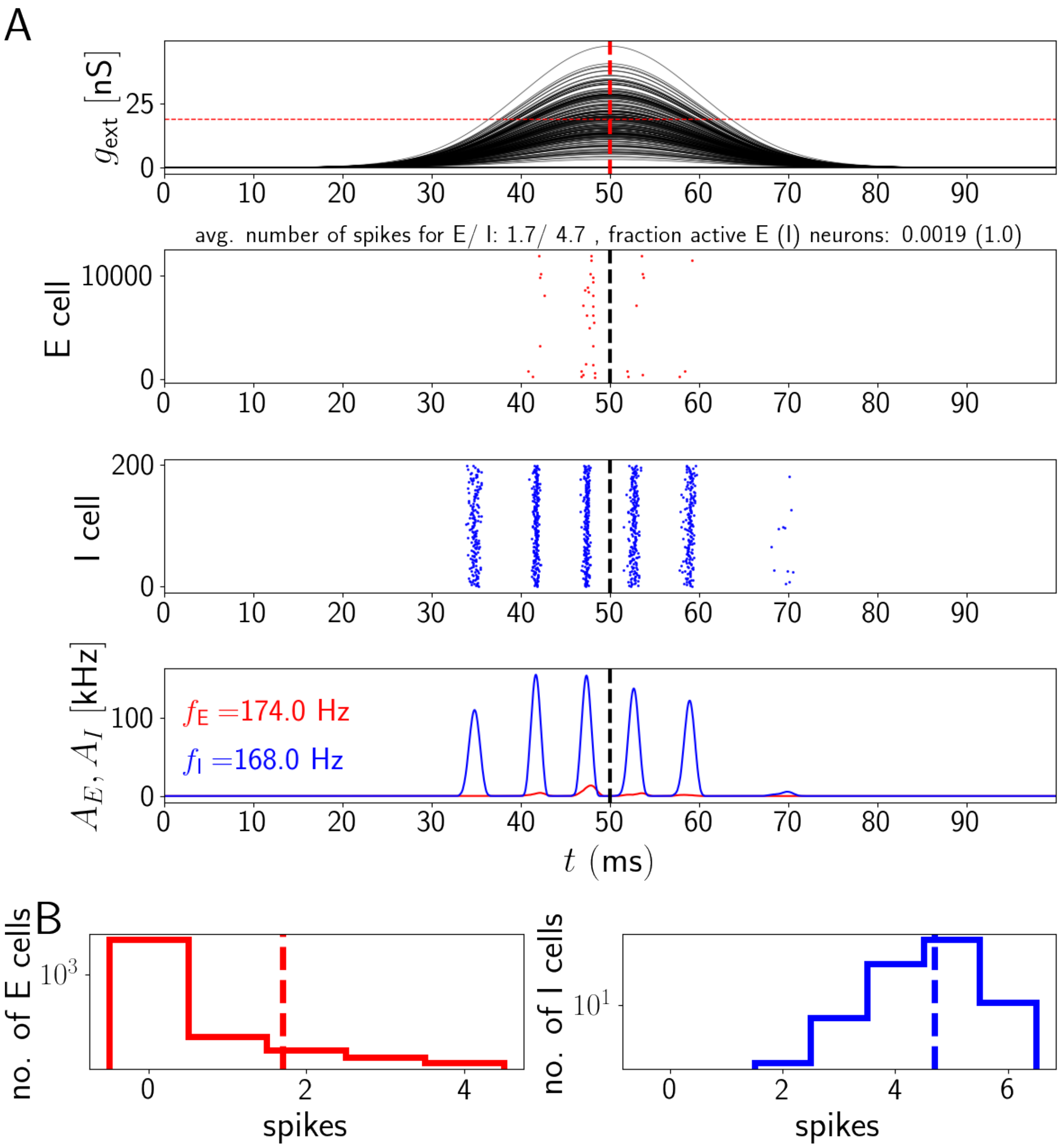

Supplement: S6 Fig — (PDF) [file pcbi.1009891.s009.pdf]
