# Supplementary material for: High-frequency oscillations and sequence generation in two-population models of hippocampal region CA1
Source: PLoS Comput Biol. 2022 Feb 17;18(2):e1009891. doi: 10.1371/journal.pcbi.1009891 (PMC8890743; doi:10.1371/journal.pcbi.1009891)

# S7 Fig

**HFOs in networks with temporally narrow excitation of E cells and broader input pulses.** Sparse spiking of E cells is largely lost in the parameter region where ripple frequency HFOs occur. Parameters are as in Fig 6, except $\sigma_g = 5$ ms. The plot layout is as in Fig 6. The frequency range for $f_I$ and $f_E$ is set to $[100, 200]$ Hz. The white circle is located at $(\bar{g}, n_E) = (90\ \text{nS}, 1330)$. In this region, HFOs exist, but E cells spike more than two times on average, which is not realistic.

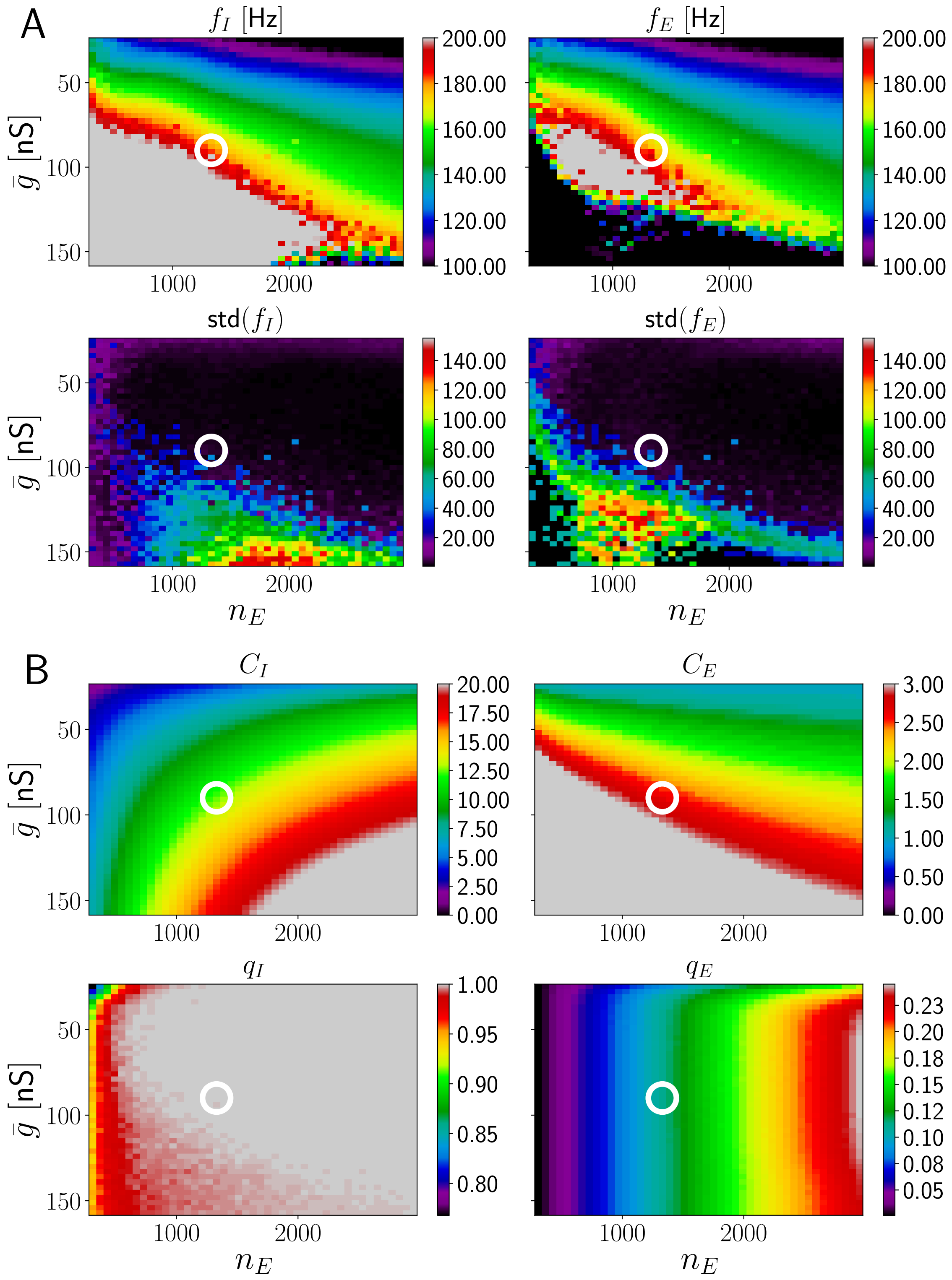

Supplement: S7 Fig — (PDF) [file pcbi.1009891.s010.pdf]
