# Supplementary material for: High-frequency oscillations and sequence generation in two-population models of hippocampal region CA1
Source: PLoS Comput Biol. 2022 Feb 17;18(2):e1009891. doi: 10.1371/journal.pcbi.1009891 (PMC8890743; doi:10.1371/journal.pcbi.1009891)

S8 Fig

HFOs in networks with temporally narrow excitation of E cells and larger I-to-E and I-to-I connectivity. Parameters are as in Fig 6, except $p_{II} = 0.3$ and $p_{IE} = 0.2$. The plot layout is as in Fig 6. The frequency range for f_I and f_E is set to $[100, 200]$ Hz. The white circle is located at $(\bar{g}, n_E) = (121\ \text{nS}, 1330)$. It indicates a region where ripple frequency oscillations and sparse E cell spiking still occur.

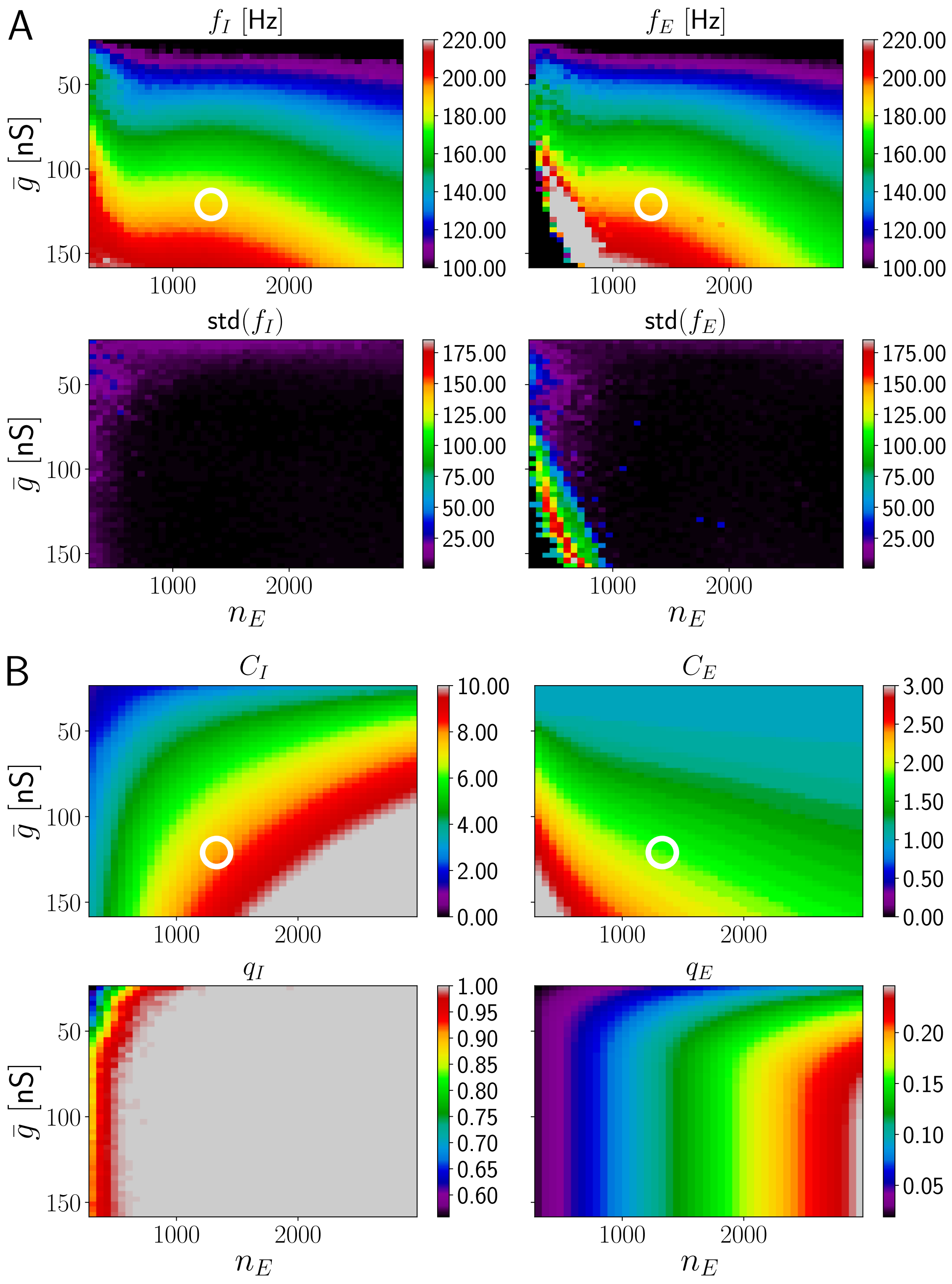

Supplement: S8 Fig — (PDF) [file pcbi.1009891.s011.pdf]
