# Supplementary material for: High-frequency oscillations and sequence generation in two-population models of hippocampal region CA1
Source: PLoS Comput Biol. 2022 Feb 17;18(2):e1009891. doi: 10.1371/journal.pcbi.1009891 (PMC8890743; doi:10.1371/journal.pcbi.1009891)

S10 Fig

HFOs in networks with temporally narrow excitation of E cells and broader spread of individual input pulses as well as higher I-to-E connectivity. Parameters are as in Fig 6, except $\sigma_t = 15$ ms and $p_{IE} = 0.2$. The plot layout is as in Fig 6. The frequency range for f_I and f_E is set to $[100, 200]$ Hz.

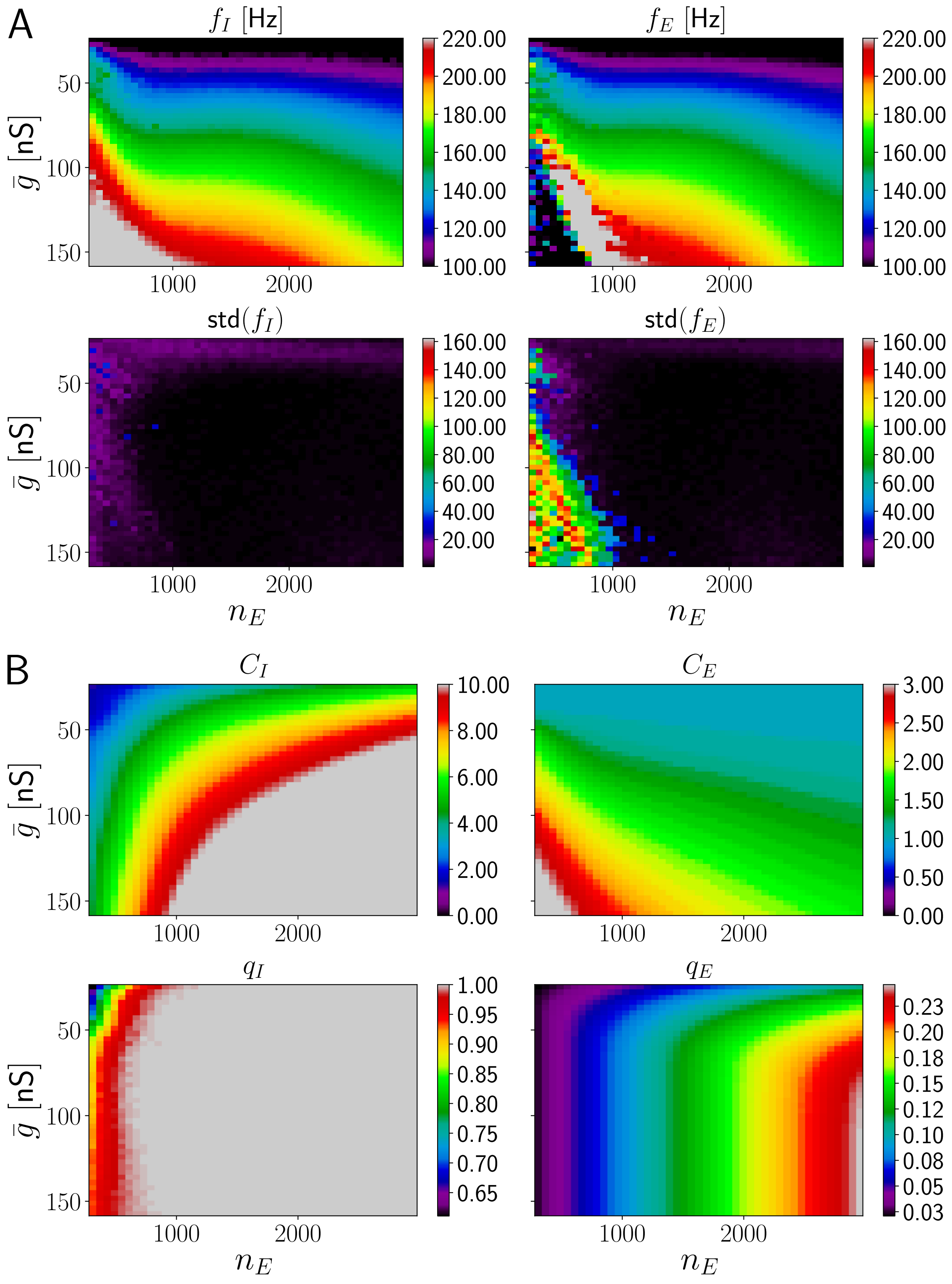

Supplement: S10 Fig — (PDF) [file pcbi.1009891.s013.pdf]
