# Supplementary material for: High-frequency oscillations and sequence generation in two-population models of hippocampal region CA1
Source: PLoS Comput Biol. 2022 Feb 17;18(2):e1009891. doi: 10.1371/journal.pcbi.1009891 (PMC8890743; doi:10.1371/journal.pcbi.1009891)

S11 Fig

HFOs in networks incorporating dendritic excitation and higher I-to-E connection probability. Parameters are as in Fig 8, except $p_{IE} = 0.3$ instead of 0.1. The plot layout is as in Fig 8. The frequency range for f_I and f_E is set to $[100, 200]$ Hz. The white circle indicates a region where HFOs in the ripple range are generated and E cells fire sparsely. It is located at $(\sigma, \mu) = (1.1, 0.0)$. At this point, HFOs with frequency ~ 190 Hz are generated and E cells fire sparsely.

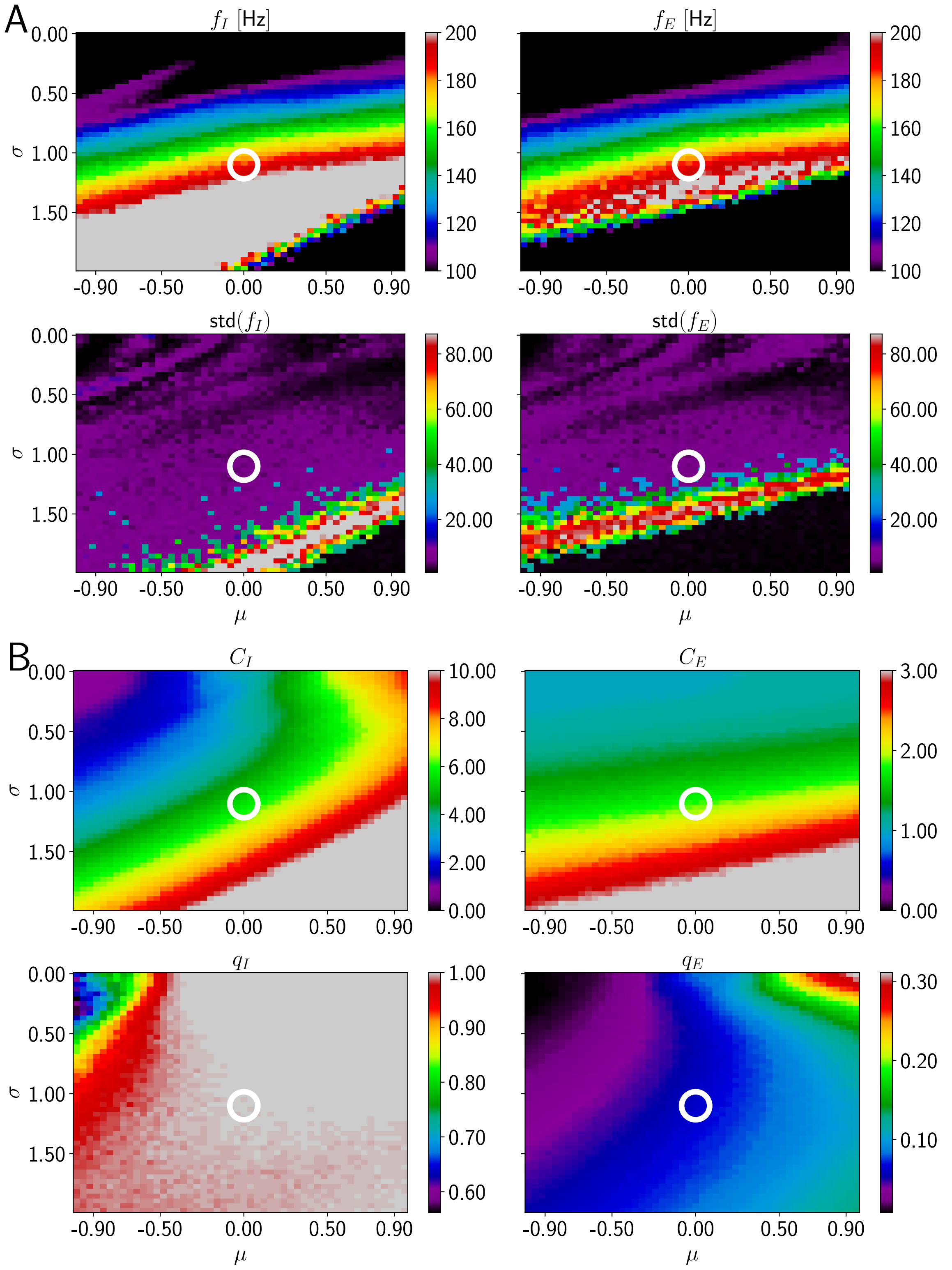

Supplement: S11 Fig — (PDF) [file pcbi.1009891.s014.pdf]
