# Supplementary material for: High-frequency oscillations and sequence generation in two-population models of hippocampal region CA1
Source: PLoS Comput Biol. 2022 Feb 17;18(2):e1009891. doi: 10.1371/journal.pcbi.1009891 (PMC8890743; doi:10.1371/journal.pcbi.1009891)

S12 Fig

HFOs in networks incorporating dendritic excitation and higher I-to-E synaptic latency.
Parameters are as in Fig 8, except for a higher I-to-E latency $\tau_l = 0.9$ ms instead of 0.5 ms. The plot layout is as in Fig 8. The frequency range for f_I and f_E is set to $[100, 200]$ Hz. The white circle is located at $(\sigma, \mu) = (0.75, 0.0)$ as in Fig 8. It is now in a region where HFOs slightly below the ripple range are generated and E cells fire sparsely.

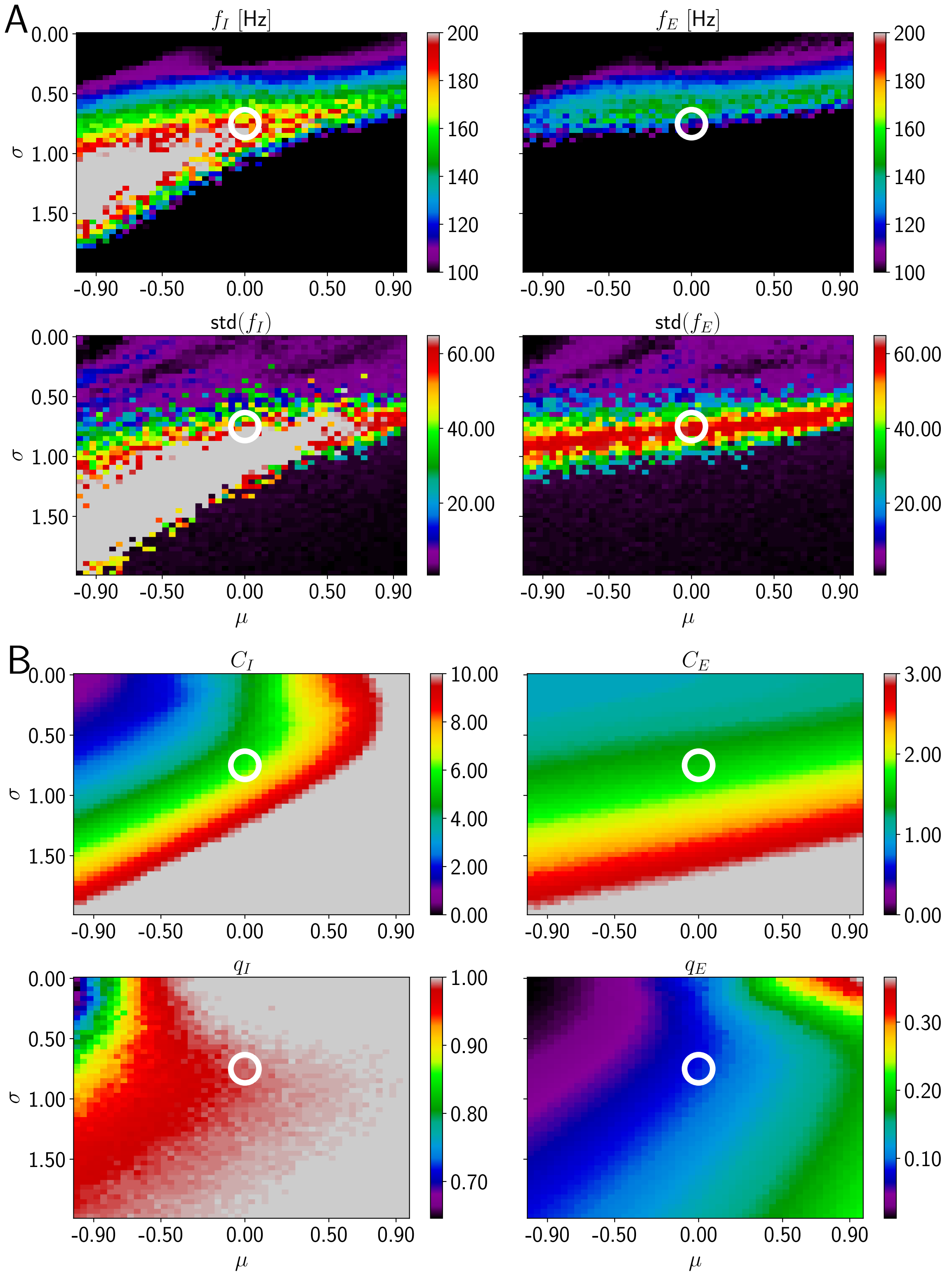

Supplement: S12 Fig — (PDF) [file pcbi.1009891.s015.pdf]
