# Supplementary material for: High-frequency oscillations and sequence generation in two-population models of hippocampal region CA1
Source: PLoS Comput Biol. 2022 Feb 17;18(2):e1009891. doi: 10.1371/journal.pcbi.1009891 (PMC8890743; doi:10.1371/journal.pcbi.1009891)

S14 Fig

HFOs in networks incorporating dendritic excitation and higher I-to-E latency, faster E-to-I rise time and lower E-to-I peak conductance. Parameters are as in Fig 8, except for a higher I-to-E latency $\tau_l = 1.0$ ms instead of 0.5 ms, faster E-to-I rise time $\tau^I_{\mathrm{exc},r} = 0.1$ ms instead of 0.5 ms and lower E-to-I peak conductance $g^I_{\mathrm{exc,peak}} = 1$ nS instead of 3 nS. The plot layout is as in Fig 8. The frequency range for f_I and f_E is set to $[100, 200]$ Hz. The white circle indicates a region where HFOs in the ripple range are generated and E cells fire sparsely. It is located at $(\sigma, \mu) = (0.75, 0.0)$.

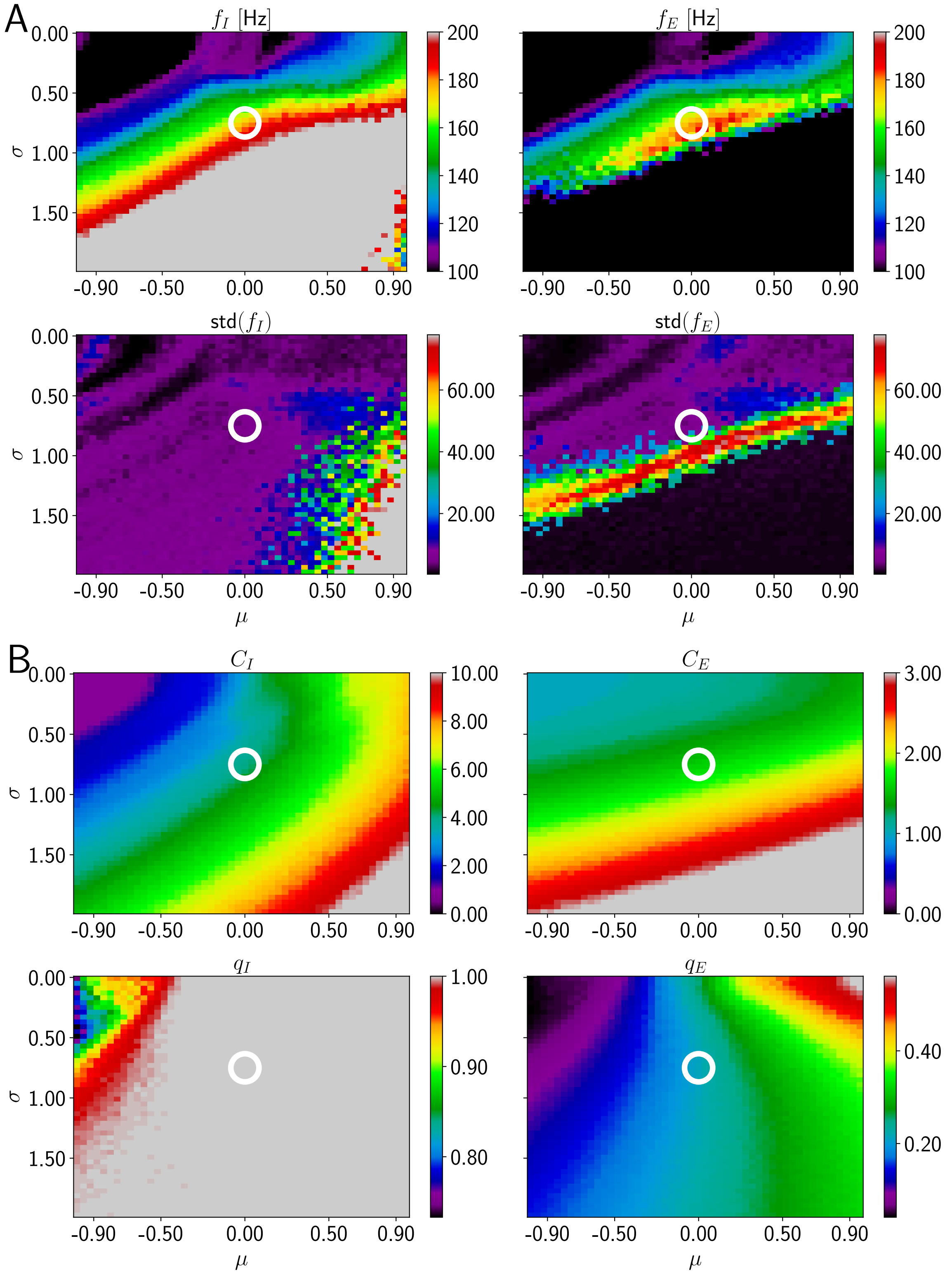

Supplement: S14 Fig — (PDF) [file pcbi.1009891.s017.pdf]
