# Supplementary material for: High-frequency oscillations and sequence generation in two-population models of hippocampal region CA1
Source: PLoS Comput Biol. 2022 Feb 17;18(2):e1009891. doi: 10.1371/journal.pcbi.1009891 (PMC8890743; doi:10.1371/journal.pcbi.1009891)

# S15 Fig

**HFOs in networks incorporating dendritic excitation and a truncated distribution for the peak dendritic current.** Parameters are as in Fig 8, except for a truncation of the lognormal distribution Eq 2 at 4 nA. The plot layout is as in Fig 8. The frequency range for $f_I$ and $f_E$ is set to $[100, 200]$ Hz. The white circle indicates a region where HFOs in the ripple range are generated and E cells fire sparsely. It is located at $(\sigma, \mu) = (0.75, 0.0)$.

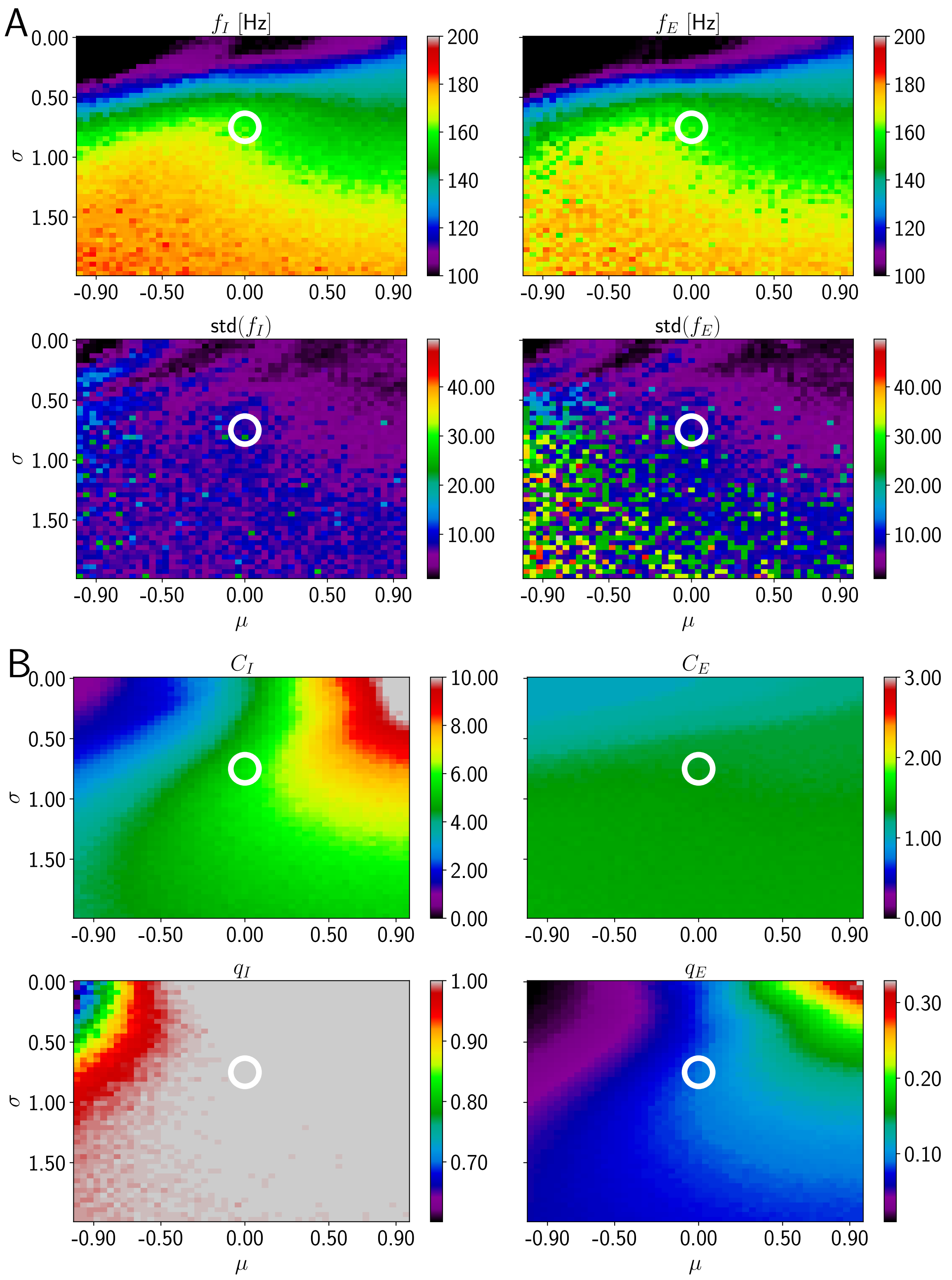

Supplement: S15 Fig — (PDF) [file pcbi.1009891.s018.pdf]
