# Supplementary material for: High-frequency oscillations and sequence generation in two-population models of hippocampal region CA1
Source: PLoS Comput Biol. 2022 Feb 17;18(2):e1009891. doi: 10.1371/journal.pcbi.1009891 (PMC8890743; doi:10.1371/journal.pcbi.1009891)

S16 Fig

HFOs in networks incorporating dendritic excitation and a truncated re-sampled distribution for the peak dendritic current as well as lower E-to-I peak conductance. Parameters are as in Fig 8, except for a truncation of the lognormal distribution Eq 2 at 4 nA with re-sampling of values larger than the truncation value and lower E-to-I peak conductance $g^{I}_{\text{exc,peak}} = 1$ nS instead of 3 nS. The plot layout is as in Fig 8. The frequency range for f_I and f_E is set to $[100, 200]$ Hz. The white circle is located at $(\sigma, \mu) = (0.75, 0.0)$. It indicates a region where HFOs in the ripple range are generated and E cells fire sparsely.

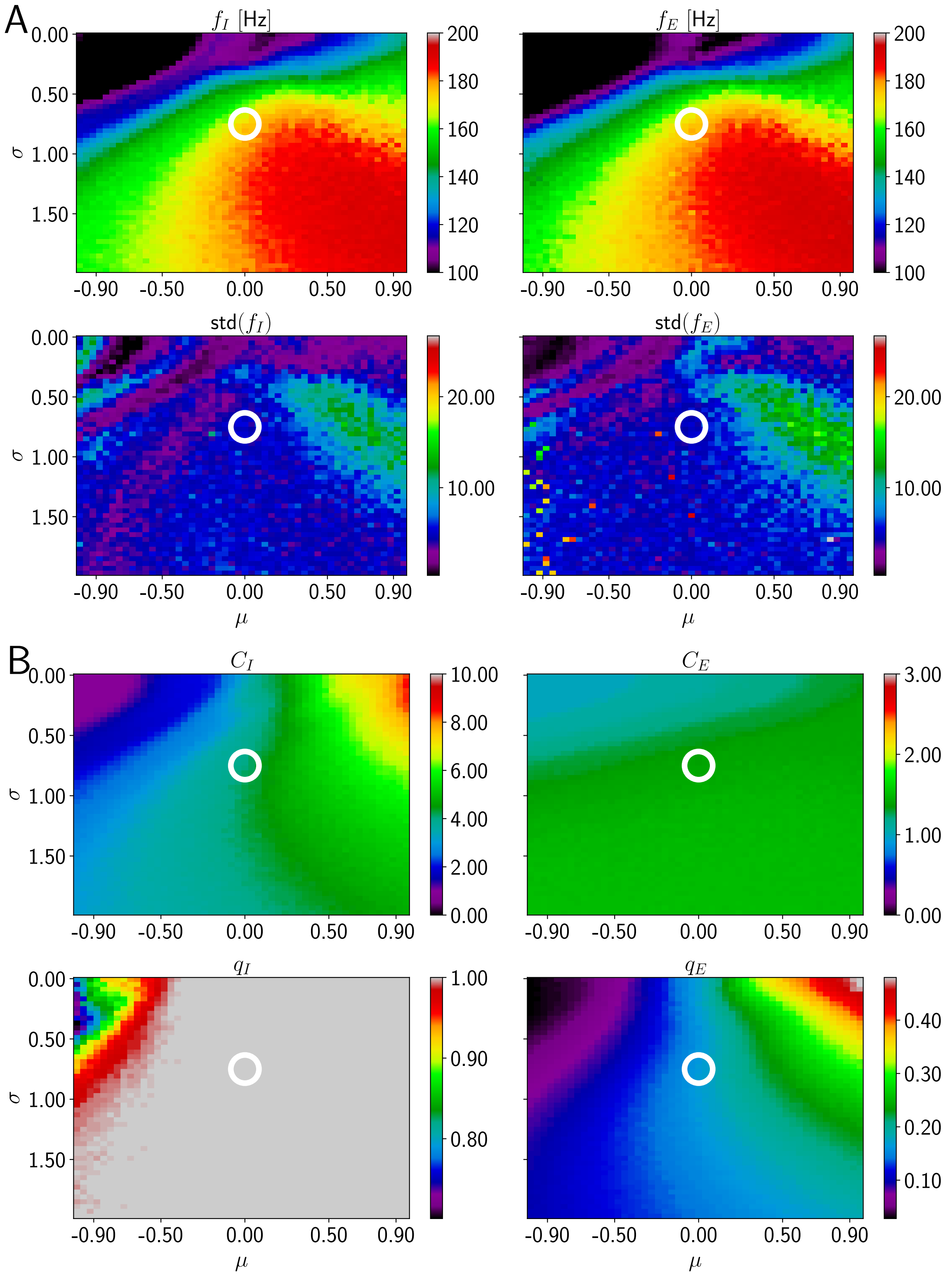

Supplement: S16 Fig — (PDF) [file pcbi.1009891.s019.pdf]
