# Supplementary material for: High-frequency oscillations and sequence generation in two-population models of hippocampal region CA1
Source: PLoS Comput Biol. 2022 Feb 17;18(2):e1009891. doi: 10.1371/journal.pcbi.1009891 (PMC8890743; doi:10.1371/journal.pcbi.1009891)

S17 Fig

HFOs in networks incorporating dendritic excitation and a Gaussian distribution for the peak dendritic current as well as lower E-to-I peak conductance. Parameters are in Fig 8, except for a Gaussian distribution of the peak dendritic current ($I^{\text{peak}}_{\text{dendritic}}/\text{nA} \sim \mathcal{N}(\mu_{\text{Gaussian}}(\mu,\sigma), \sigma_{\text{Gaussian}}(\mu,\sigma))$ and lower E-to-I peak conductance $g^{I}_{\text{exc,peak}} = 1$ nS instead of 3 nS. The plot layout is as in Fig 8. The mean and standard deviation of the Gaussian distribution are matched to the mean and standard deviation of a lognormal distribution with parameters μ and σ: $\mu_{\text{Gaussian}}(\mu,\sigma) = \exp\left(\mu + \frac{\sigma^2}{2}\right)$, $\sigma_{\text{Gaussian}}(\mu,\sigma) = \left[\exp\left(\sigma^2\right) - 1\right] \exp\left(2\mu + \sigma^2\right)$; to allow a direct comparison with the previous figures, the parameters μ and σ are given on the plot axes. The frequency range for f_I and f_E is set to $[100, 200]$ Hz. The white circle is located at $(\sigma, \mu) = (0.75, 0.0)$. It indicates a region where HFOs in the ripple range are generated and E cells fire sparsely.

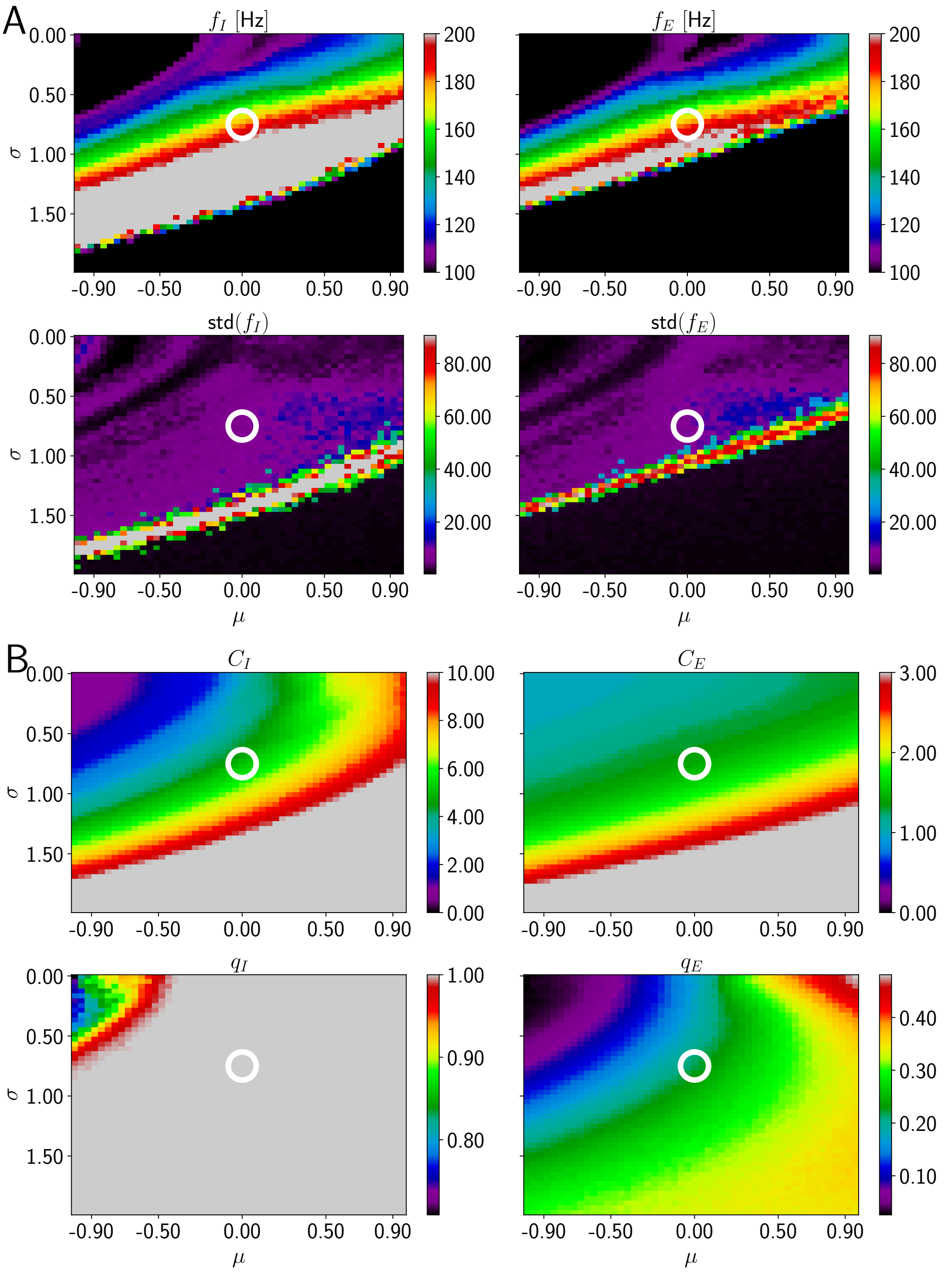

Supplement: S17 Fig — (PDF) [file pcbi.1009891.s020.pdf]
