# Supplementary material for: High-frequency oscillations and sequence generation in two-population models of hippocampal region CA1
Source: PLoS Comput Biol. 2022 Feb 17;18(2):e1009891. doi: 10.1371/journal.pcbi.1009891 (PMC8890743; doi:10.1371/journal.pcbi.1009891)

S18 Fig

HFOs in networks incorporating dendritic excitation and a long somatic absolute refractory period for the E cells. Parameters are as in Fig 9, except for an absolute somatic refractory period $\tau^E_{\mathrm{ref}} = 200$ ms. The plot layout is as in Fig 8. Every active E cell spikes once. The frequency range for f_I and f_E is set to $[100, 200]$ Hz. The white circle is located at $(\sigma, \mu) = (0.75, 0.0)$. It indicates a region where HFOs in the ripple range are generated and E cells fire sparsely.

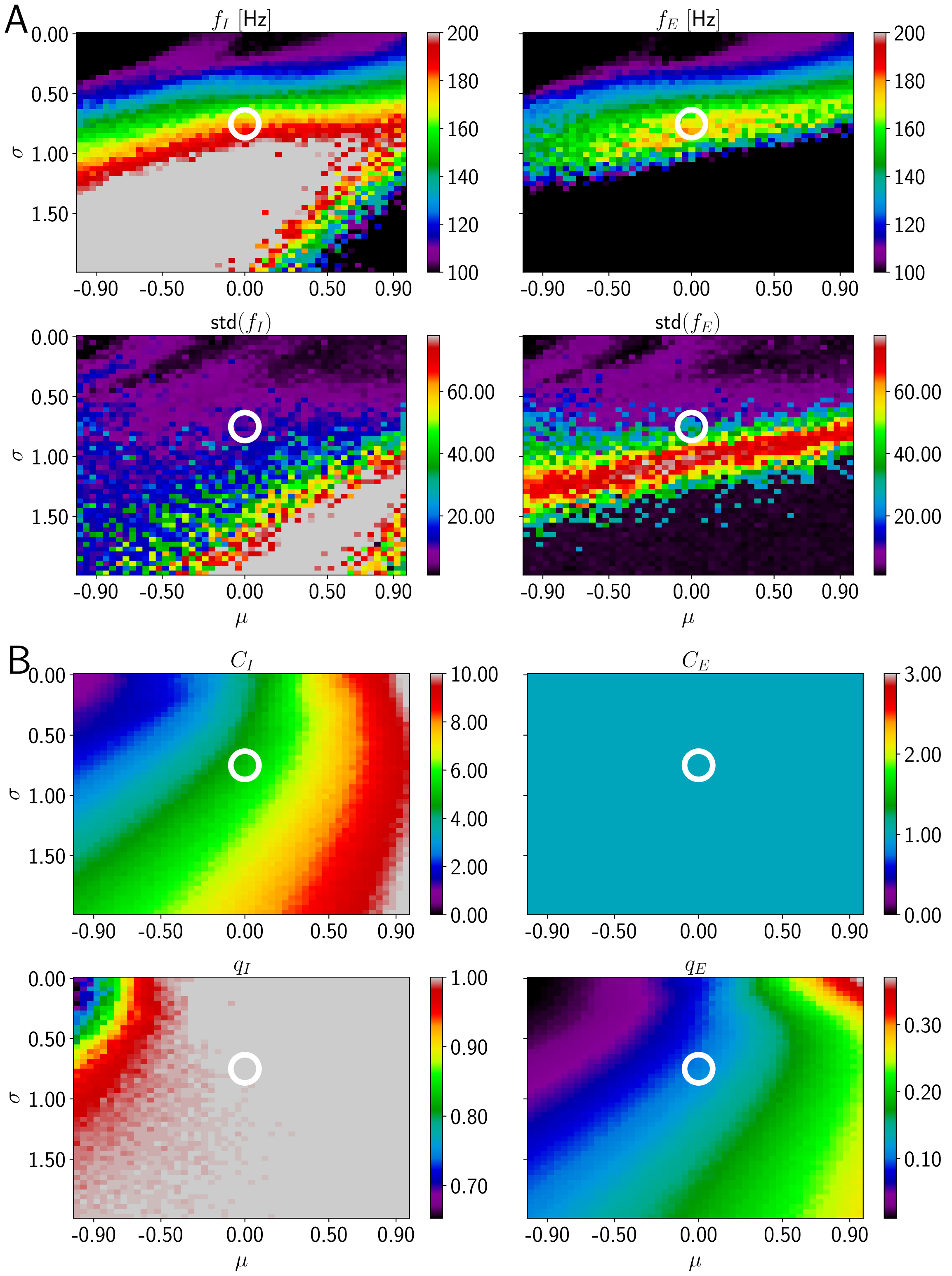

Supplement: S18 Fig — (PDF) [file pcbi.1009891.s021.pdf]
