# Supplementary material for: High-frequency oscillations and sequence generation in two-population models of hippocampal region CA1
Source: PLoS Comput Biol. 2022 Feb 17;18(2):e1009891. doi: 10.1371/journal.pcbi.1009891 (PMC8890743; doi:10.1371/journal.pcbi.1009891)

# S19 Fig

**Further alternative concepts of replay.**
Further connectivity schemes for sequence generation without recurrent excitation. (A) Scheme for sequence generation by pulse coding of both E and I cells. In contrast to Fig 9 A, the $k$th replay step is in the I neurons reflected by an activation of group $I_k$. Each group $E_k$ projects to one group of inhibitory neurons, to $I_k$, as displayed for $E_1$. $I_k$ projects to all $E_l$ with $l \neq k+1$ as displayed for $I_1$. The green dashed line denotes the absent connections from group $I_1$ to group $E_2$. Group $E_0$ is stimulated to fire first and the replay event progresses along the black arrow: $E_0$ activates $I_0$, which inhibits all E cells except those of $E_1$. Thus, in the next step the group $E_1$ becomes active and stimulates $I_1$, which leads to activation of $E_2$ etc. (B) Scheme for sequence generation by gap coding of I cells. In contrast to Fig 9 A and panel A of this figure, the sequence generation relies on the inhibitory population alone, no E-to-I connections are necessary. The $k$th replay step is in the inhibitory neurons reflected by a deactivation of group $I_k$, as in Fig 9 A. There are $K+1$ inhibitory groups $I_0$ to $I_K$. Neurons of group $I_0$ project to all inhibitory neurons except to those of $I_1$ (displayed by dashed green line). Similarly, group $I_1$ does not project to group $I_2$, but to all other inhibitory groups. Group $I_1$ thus receives projections from groups $I_1$ to $I_K$, group $I_2$ receives projections from groups $I_0$ and $I_2$ to $I_K$ and so on. Every inhibitory group gets projections from $K$ inhibitory groups (in the figure: $K = 4$). In the first replay step, group $I_0$ is silent, whereas all other groups fire. In the next step, every group $I_l$, $l > 0$, except $I_1$ receives the same amount of inhibition from only $K-1$ groups, namely from the previously active groups $I_1$ to $I_{l-2}$ and $I_l$ to $I_K$, which project to it. The input from $I_0$ is missing, since it was not active. $I_1$ receives more inhibition than the other groups because all its $K$ inhibitory presynaptic groups were active in the previous step (the silence of $I_0$ does not reduce $I_1$'s inhibitory input, since $I_0$ does not project to $I_1$). Thus, after the zeroth cycle, group $I_1$ is silent while the other groups are active. In the next cycle, group $I_2$ will be silent because it received more inhibition than the other groups and so on. We note that $I_0$ also gets inhibitory input from $K$ groups in each step because it has no predecessor group not inhibiting $I_0$ but all other groups; its neurons should thus receive a compensatory excitatory input throughout sequence generation to be active after the zeroth step. The E cells in group $E_k$ can be entrained in this scheme by disinhibition, via inhibitory connections from group $I_k$ to $E_k$.

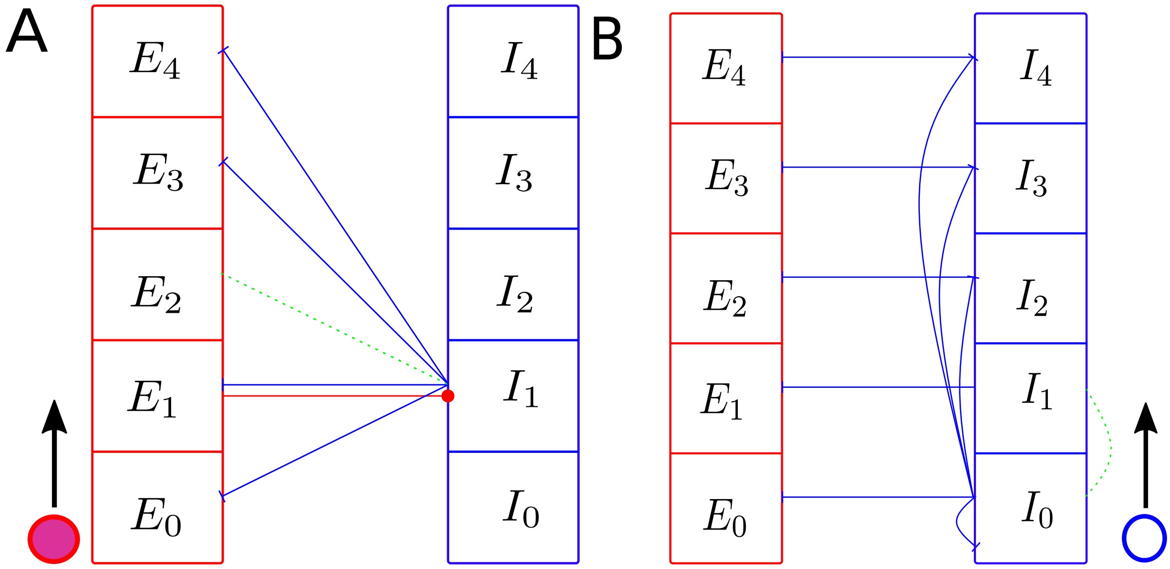

Supplement: S19 Fig — (PDF) [file pcbi.1009891.s022.pdf]
